# Synthetic Design and Biological Evaluation of New p53-MDM2 Interaction Inhibitors Based on Imidazoline Core

**DOI:** 10.3390/ph15040444

**Published:** 2022-04-02

**Authors:** Daniil R. Bazanov, Nikolay V. Pervushin, Egor V. Savin, Michael D. Tsymliakov, Anita I. Maksutova, Victoria Yu. Savitskaya, Sergey E. Sosonyuk, Yulia A. Gracheva, Michael Yu. Seliverstov, Natalia A. Lozinskaya, Gelina S. Kopeina

**Affiliations:** 1 Department of Chemistry, M. V. Lomonosov Moscow State University, 119992 Moscow, Russia; daniil_bazanov@mail.ru (D.R.B.); tsymmd@gmail.com (M.D.T.); anita.maksutova@gmail.com (A.I.M.); svk1896@mail.ru (V.Y.S.); umpolung@yandex.ru (S.E.S.); jullina74@mail.ru (Y.A.G.); timewalker@yandex.ru (M.Y.S.); 2 Department of Medicine, M. V. Lomonosov Moscow State University, 119991 Moscow, Russia; rhododendron.nick@mail.ru (N.V.P.); savineg48@yandex.ru (E.V.S.)

**Keywords:** nutlins, cis-imidazolines, p53-MDM2 inhibitors, anti-cancer

## Abstract

The use of p53-MDM2 inhibitors is a prospective strategy in anti-cancer therapy for tumors expressing wild type p53 protein. In this study, we have applied a simple approach of two-step synthesis of imidazoline-based alkoxyaryl compounds, which are able to efficiently inhibit p53-MDM2 protein–protein interactions, promote accumulation of p53 and p53-inducible proteins in various cancer cell lines. Compounds **2l** and **2k** cause significant upregulation of p53 and p53-inducible proteins in five human cancer cell lines, one of which possesses overexpression of MDM2.

## 1. Introduction

p53 is a “guardian of genome”, which is important for maintenance of genomic stability in normal and pathological conditions. This multidomain protein is a transcription factor, which plays a pivotal role in regulation of programmed cell death (PCD), the cell cycle and cellular responses to various stresses [1]. TP53 is one of the most frequently mutated genes in cancer tissues. The majority of its mutations lead to the loss of oncosuppressive functions of wild-type p53 protein. Moreover, these mutations commonly cause tumor progression and chemoresistance [2,3]. The MDM2 (mouse double-minute 2) protein is a negative regulator of p53, which promotes proteasome-dependent degradation of the last thorough E3 ubiquitin ligase activity. Additionally, MDM2 can inhibit p53 via physical occlusion and activation of its nuclear export [4]. 

The chemical compounds, which are able to block p53-MDM2 protein–protein interaction (PPI) and induce p53 activation, have a high potential for the treatment of different tumors expressing wild-type p53 [5,6]. An accumulation of p53 leads to the cell cycle arrest, the PCD activation and an initiation of DNA repair [7]. Nutlins, *cis*-substituted imidazolines, were first reported as the potent and specific small molecule inhibitors of p53-MDM2 PPI [8,9]. Nowadays, after the optimization of their structure, two compounds, RG7112 and RG7388, are being evaluated in several clinical trials (NCT00623870, NCT02670044, NCT02407080) [10,11,12].

Notably, a specific feature of the p53-MDM2 PPI is the high hydrophobicity of the binding surface. Hydrophobic molecules, which are able to destroy this PPI, are hard to use in clinical applications due to undesirable pharmacological properties [13,14,15]. The core of nutlins is highly hydrophobic so the whole molecules are insoluble in water. In addition, *cis*-imidazoline core is able to oxidize to simple aromatic imidazole that leads to loss of activity, and methoxy groups in the Nutlin-3a are able to hydrolyze during metabolism [10]. These drawbacks were remarkably solved in the design of RG7112 molecule [11]. Nevertheless, the core has remained without significant changes in haloaryl substituents that occur due to the reluctance to change the active center of the molecule, because para-chlorophenyl substituents perfectly occupy the pockets of Leu26 and Trp23 and the synthesis of other vicinal diamines as precursors of imidazoline core is complicated. 

Previously, we have shown that the replacement of halogen in the imidazoline core with a methoxy group significantly improved its water solubility and novel compounds were also able to increase the level of p53 [16]. The most promising cores among alkoxyaryl compounds were the 2,4-dimethoxyphenyl-based derivatives, although the effect of p53 stabilization remained rather weak [17]. Herein, we propose a novel modification of previously found imidazolines in order to increase their biological effect using the 2,4,5-tris(alkoxyphenyl)imidazoline core. The synthesis of 2,4,5-trimethoxyphenylimidazolines in one step from the corresponding arylaldehydes makes this approach attractive for the synthetic strategy and atom economy.

## 2. Results

### 2.1. Chemical Synthesis

**Figure 1:**
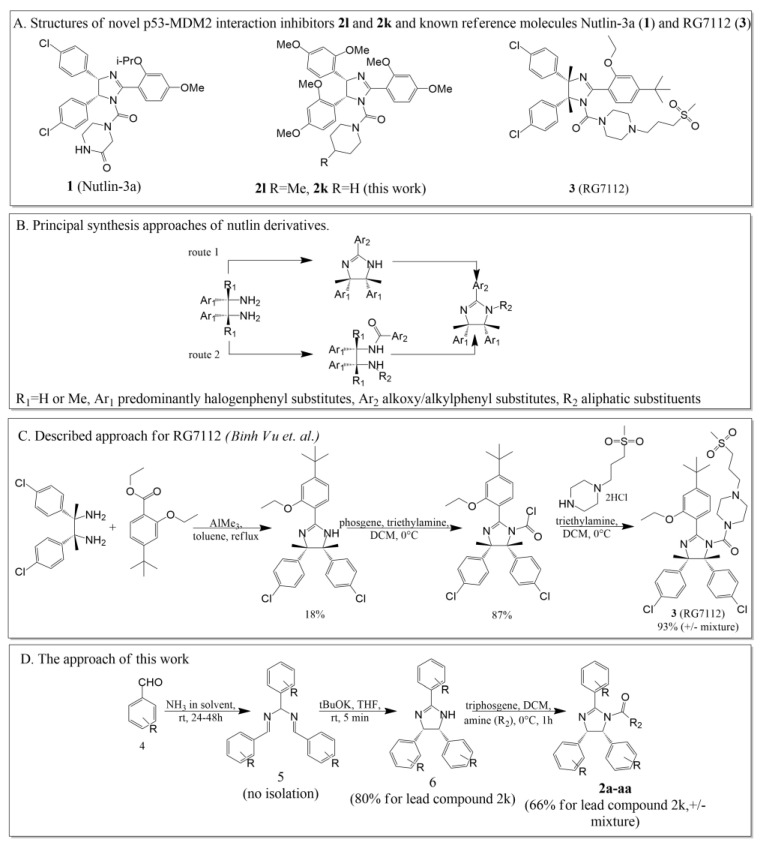
(**A**). The structures of novel p53-MDM2 interaction inhibitors **2l** and **2k** and known reference molecules Nutlin-3a (1) and RG7112 (3). (**B**). The principal synthesis approaches for nutlin derivatives. (**C**). The described approach for the synthesis of RG7112 [10]. (**D**). The developed approach.

There are two principal approaches to synthesize nutlin derivatives. A starting compound in both approaches is a vicinal diamine with the required *erythro*-configuration. In the case of nutlin-3a, it was obtained using the previously described method via the interaction of aldehydes with a source of ammonia [18]. In the case of the analogue RG7112 [10], the starting diamine was a butane derivative (Figure 1). Further, the first approach assumes the ring closure with the formation of an imidazoline ring followed by modification at the nitrogen atom. The second, in turn, is based on the selective modification of amino groups with substituents followed by cyclization of the obtained diamides. Both approaches involve different substituents at the positions of the imidazoline core but possess a low overall yield. For example, the original creation of RG7112 involves the synthesis of the imidazoline core from diamine with 18% yield. Scaling up the synthesis of the imidazoline core from diamines and the corresponding benzoic acid derivative were presented in [19]. 

A fundamentally different synthetic approach, described by us earlier [16,17,20], involved the direct preparation of the *cis*-imidazoline core from aldehydes by their reaction with ammonia in the appropriate solvents. This method allowed us to obtain diazapentadiene **5** (Figure 1D) followed by disrotatory closure in the presence of a base that led to a synthesis of the imidazoline core **6**. The reaction yields at this stage reach up to 95%. The unreacted aldehyde can be regenerated. Despite the fact that this approach makes it possible to obtain only imidazolines with the same substituents, these compounds have been shown to retain the ability of nutlins to inhibit the p53-MDM2 interaction and can serve as their cheap analogs for further modification.

Previously, we synthesized a series of cis-imidazolines and determined the most active of them, the 2,4-dimethoxyphenyl derivative. The modification of the nitrogen atom by sulfonic derivatives has been carried out [16]. Here, we performed the further modification of imidazoline derivatives at the nitrogen atom using triphosgene and appropriate amines with good yields (Figure 1D, Table 1) [21,22]. To compare both synthetic features and biological activity, we synthesized not only 2,4-dimethoxyphenyl derivatives, but also a number of other alkoxy and halogen derivatives.

### 2.2. In Silico Studies

**Figure 2:**
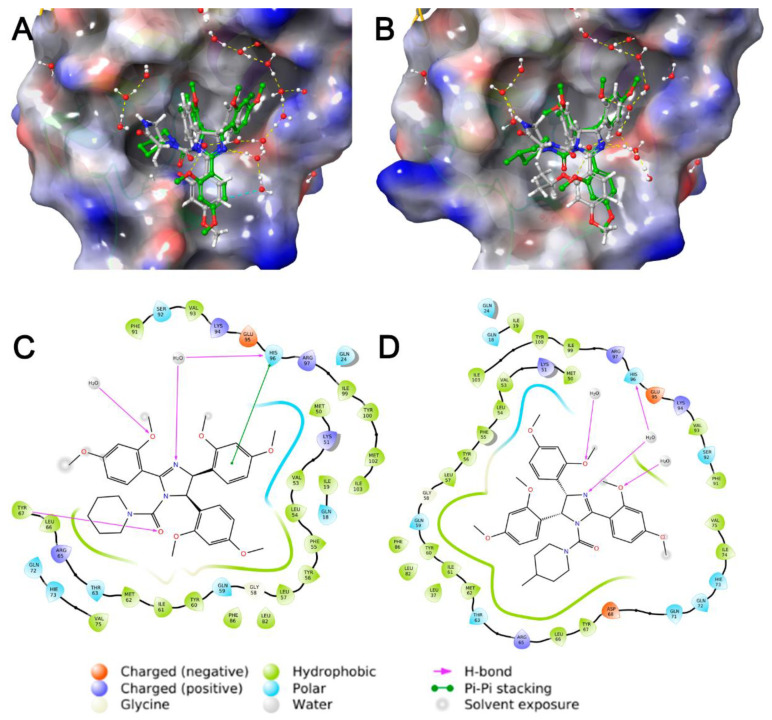
Docked poses for **2k** (**A**) and **2l** (**B**) (green), in p53 binding site of MDM2 protein, in comparison to nutlin-3a (grey). Ligand-protein interaction map for **2k** (**C**) and **2l** (**D**) in p53 binding site of MDM2 protein.

To clarify the binding of cis-imidazolines with amido-substituents to MDM2 protein, molecular docking of its p53 binding site was performed for 2,4-dimethoxyphenyl-based imidazolines **2i**–**m** and 3,4-dimethoxyphenyl-based compound **2n** (Table 1, Figure 2 and Supplementary Figures S1–S3). Molecular dynamics simulations were carried out for active compounds **2k** and **2l** to refine binding modes in a solvating vicinity (water filled box) and to test the stability of the obtained positions. As a result, the selected positions (Figure 2) were assessed as stable for more than 5 ns based on time-dependent convergence of coordinate RMSD (root-mean-square deviation). The binding profile of nutlin-3a to MDM2 protein (PDB 4HG7) was used for all performed computations. 

All selected compounds were able to dispose in the p53 binding region of MDM2 with appropriate estimated energies (Figure 2). They occupied the same binding pockets and had roughly the same docking pose as nutlin-3a, but these slight differences allowed them to interact with other amino acid residues, such as Tyr67 or Phe91. This feature gave us an additional hydrogen bond or π-π stacking for our compounds, whereas for nutlin-3a, this interaction was constrained because of its bulky isopropyl-substituent. On the other hand, bigger substituents in position 1 and in aromatic rings in positions 4, 5 (e.g., -OMe in **2l** compared to -Cl in nutlin-3a) could attenuate an important interaction with His96, increasing the distance between the stacking aromatic systems. Thus, **2l** interacted with His96 through a hydrogen bond with a structural water molecule. Alkoxyaryl groups in the external part of the site allowed solvent interactions, while internal methoxyphenyl groups provided hydrophobic interactions (e.g., with Val53, Phe55 and Tyr56). Moreover, different methoxy groups and the nitrogen atom in position 3 of imidazoline ring participated in numerous hydrogen bonds with structural water that was a significant advantage of the proposed scaffold compared to nutlin-3a.

The docking poses of **2k** and **2l** (Figure 2) are based on an overall great geometric resemblance due to their structural similarity. Compound **2l** demonstrated weaker interactions with His96 and Tyr67 at distances, which could not be considered as a full π-π or hydrogen bond. Probably, the methyl substituent in **2l** was too bulky for the corresponding hydrophobic pocket near Tyr67. This feature might result in some instability of the binding site, altering the interactions of the derivate with MDM2 and slightly changing the biological activity.

### 2.3. Biology

To analyze the biological activity of the synthesized compounds, we have tested the ability of these molecules to stabilize p53 and induce expression of p53-dependent genes -*CDKN1A* and *BBC3* encoding p21 and Puma (p53 upregulated modulator of apoptosis), correspondently [23,24]. p21, a cyclin-dependent kinase inhibitor, plays an important role in the cell cycle arrest and protects cells from different stressful stimuli including DNA damage [25]. Moreover, p21 takes part in regulation of apoptosis, a form of PCD. Puma, a pro-apoptotic member of the Bcl-2 family proteins, is able to inhibit anti-apoptotic proteins of this family that result in apoptosis activation via intrinsic pathway [24,26]. Importantly, Nutlin-3 interrupts p53-MDM2 PPI and leads to stabilization of p53 protein that, in turn, causes an increase in p21 and Puma levels.

We have selected 22 compounds, whose structures were delineated in Table 1, for screening of biological activity in colon carcinoma cell line RKO expressing wild-type p53 [27]. A series of derivatives containing the 2,4-dimethoxyphenyl fragment was selected for screening due to previously shown promising results [17]. Other compounds from this group were chosen to assess the structure-property relationship.

**Figure 3:**
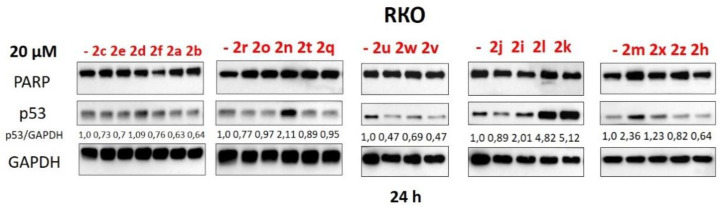
Western Blot analysis of total cellular lysates from RKO cells upon treatment with compounds: A (**2a**–**2f**), B (**2n**, **2o**, **2q**, **2r**, **2t**), C (**2u–2w**), D (**2i–2l**), E (**2h**, **2m**, **2x**, **2z**). GAPDH was used as a loading control. The concentrations of all derivatives—20 μM. PARP—poly (ADP-ribose)-polymerase; GAPDH—glyceraldehyde 3-phosphate dehydrogenase; p53/GAPDH—densitometric analysis of p53 bands normalized to GAPDH, h—hours.

RKO cells were treated with these compounds in the concentrations of 20 μM since earlier we had found that it was the most efficient concentration for the alkoxy-derivates developed by us [17]. After 24 h incubation, p53 levels in the treated cells were estimated using Western blot (WB). Among all the compounds (Figure 3), two derivatives (**2l** and **2k**) have demonstrated promising results. Their administration led to 4.82-(**2l**) and 5.12-fold (**2k**) increase in p53 compared to non-treated cells (Figure 3). These compounds were selected for further investigation. According to the MTT-test, **2l** and **2k** were comparable to Nutlin-3a IC50 [28] (Supplementary Table S1).

Next, we have analyzed the biological activity of **2l** and **2k** (20 μM) in RKO cells compared to Nutlin-3a (10 μM) [29] and RG7388 (5 μM), which were used at micromolar concentrations as the positive controls of p53-MDM2 inhibition. 

**Figure 4:**
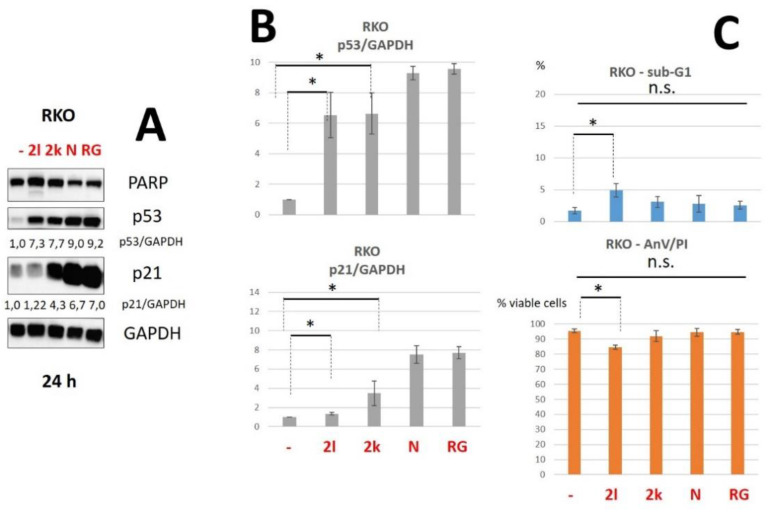
(**A**). Western Blot analysis of total cellular lysates from RKO cells upon the treatment with compounds **2l**, **2k** (both—20 μM), Nutlin-3a (10 μM) and RG7388 (5 μM). (**B**)—Densitometric analysis of p53 bands normalized to GAPDH. Data are presented as mean +/− SD from three independent experiments. (**C**)—The histograms of flow cytometry (FC) analysis data for RKO cells: sub-G1 assay (up), %—percent of Sub-G1 population and Annexin V-FITC/PI staining (below), % viable cells—cells negative for both Annexin V-FITC and propidium iodide (PI). Data from n = 3 biological replicates are shown as mean ± s.d., * *p* < 0.05, n.s.—not significant. PARP—poly (ADP-ribose)-polymerase; GAPDH—glyceraldehyde 3-phosphate dehydrogenase; p53/GAPDH—densitometric analysis of p53 bands normalized to GAPDH, h—hours, N—Nutlin-3a (10 μM), R—RG7388 (5 μM).

We have found that **2l** and **2k** were able to effectively stimulate an increase in p53 and p21 levels in RKO cells. The statistically significant increase in p53 level (up to more than 7) was comparable to Nutlin-3 and RG7388. Moreover, **2k** caused the significant accumulation of p21 (Figure 4A,B).

**Figure 5:**
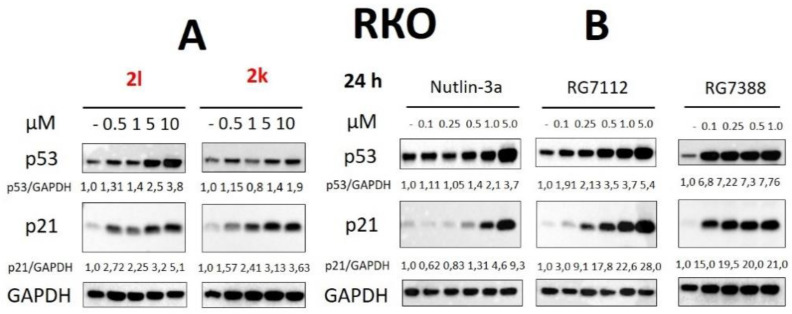
Western Blot analysis of total cellular lysates from RKO cells upon treatment with **2l**, **2k** (**A**); Nutlin-3a, RG7112 and RG7388 (**B**). GAPDH was used as a loading control. Designations: GAPDH—glyceraldehyde 3-phosphate dehydrogenase; p53/GAPDH and p21/GAPDH—densitometric analysis of p53 or p21 bands normalized to GAPDH.

Furthermore, we have tested lower concentrations of **2l** and **2k** in RKO cells. The **2l** derivative was able to stabilize p53 level in concentration range 500 nM–10 μM: from 1.31 (500 nM) to 3.8-fold (10 μM). The treatment with **2k** compound led to effective accumulation of p53 only at 5 and 10 μM. Both compounds caused a growth of p21 level starting from 500 nM (Figure 5A). Additionally, we have compared these molecules with low concentrations of Nutlin-3a, RG7112 and RG7388. Importantly, RG7112 and RG7388 demonstrated high efficacy from 100 nM, while Nutlin-3a—only up to 500 nM (Figure 5B). Moreover, 500 nM of Nutlin-3a and **2l** derivative demonstrated similar efficacy (Figure 5).

To evaluate whether synthesized compounds caused cell death, the cleavage of PARP, which is a well-known apoptotic marker, was estimated using WB analysis [30]. Among all tested compounds, only **2l** caused a slight accumulation of the cleaved PARP fragment (Figure 3 and Figure 4). To confirm the obtained data, we performed flow cytometric analysis using double-staining with Annexin V–FITC and propidium iodide (PI) which allowed us to evaluate the population of apoptotic and necrotic cells [31]. The cell death assay using Annexin V-FITC/PI staining revealed that only **2l** attenuated cell viability to some degree compared to non-treated RKO cells (Figure 4C). Similar results were demonstrated using another flow cytometric analysis, sub-G1 assay (Figure 4C), in which the percentage of sub-G1 population reflects the number of apoptotic cells [32]. 

It should be noted that Nutlin-3a (10 μM) and RG7388 (5 μM) did not decrease cell viability of RKO cells (Figure 4C). However, we decided to assess whether nutlins were able to induce cell death at high concentrations in RKO cells similar to **2l** compound. According to WB analysis, RG7112 and RG7388 (both—at 20 μM) led to the pronounced accumulation of cleaved PARP (Supplementary Figure S4A). These data were confirmed by the flow cytometric analysis using Annexin V-FITC/PI staining: RG7388 and RG7112 caused the decrease of cell viability to 73.3% and 57.5%, respectively (Supplementary Figure S4B).

**Figure 6:**
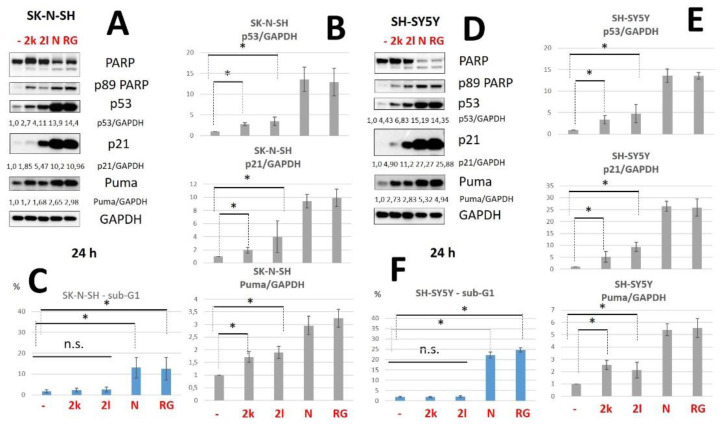
Western Blot analysis of total cellular lysates from SK-N-SH (**A**) and SH-SY5Y (**D**) cells upon the treatment with compounds **2l**, **2k** (both—20 μM), Nutlin-3a (10 μM) and RG7388 (5 μM). (**B**,**E**)—Densitometric analysis of p53, p21 and Puma bands normalized to GAPDH in SK-N-SH (**B**) and SH-SY5Y (**E**) cells. Data are presented as mean +/− SD from three independent experiments. (**C**,**F**)—The histogram of flow cytometry (FC) analysis data for SK-N-SH cells using sub-G1 assay, %—percent of Sub-G1 population. Data from n = 3 biological replicates are shown as mean ± s.d., * *p* < 0.05, n.s.—not significant. PARP—poly (ADP-ribose)-polymerase; GAPDH—glyceraldehyde 3-phosphate dehydrogenase; p53/GAPDH, p21/GAPDH and Puma/GAPDH—densitometric analysis of p53P, p21P and Puma bands normalized to GAPDH, h—hours, N—Nutlin-3a (10 μM), R—RG7388 (5 μM).

Next, we tested our compounds in two neuroblastoma cell lines, SK-N-SH and SH-SY5Y. Besides the analysis of p53 and p21, we have also evaluated the level of another p53-inducible protein, Puma. We have found that both compounds significantly upregulated levels of p53 (up to 4.11—**2l**), p21 (up to 5.47—**2l**) and Puma (up to 1.7—**2k**) (Figure 6A,B) in SK-N-SH cells. Moreover, we have shown that both derivatives also significantly upregulated levels of p53 (up to 6.83—**2l**), p21 (up to 11.2—**2l**) and Puma (up to 2.83—**2l**) (Figure 6D,E) in SH-SY5Y cells. According to the sub-G1 assay, **2l** and **2k** did not decrease cell viability in contrast to Nutlin-3a and RG7388 in both cell lines (Figure 6C,F).

Finally, we have analyzed the biological activity of two compounds **2l** and **2k** in osteosarcoma cell line SJSA-1 and prostate cancer line LNCaP. Notably, SJSA-1 is characterized by amplification of the *MDM2* gene [33]. The treatment of cells with **2l** derivate triggered an increase in p53 level (up to 3.1), p21 (up to 13.7) and Puma (up to 4.22) in both cancer cell lines (Supplementary Figure S5). Despite amplified MDM2 in SJSA-1 cells, **2l** was able to increase p53 and its target levels. Taken together, the compound **2l** was more effective than **2k** and caused the high accumulation of p53, p21 and Puma levels in the studied cancer cell lines expressing wild-type p53.

## 3. Discussion

Thus, we have developed a simple approach for obtaining new p53-MDM2 inhibitors based on 2,4,5-tris(methoxyphenyl)imidazolines. This two-step approach allows us to synthesize the compound **2l** in gram scale with a total yield of 55 percent. The binding poses with the active site of MDM2 were shown using the method of molecular modeling. We have found that the treatment with **2l** caused the significant upregulation of p53 and p53-inducible proteins in 5 human cancer cell lines. Our results have demonstrated that the nutlin’s core could be modified to optimize its water solubility without loss of the biological activity. Moreover, the developed synthetic strategy allowed us to reduce the number of steps and might help to improve the pharmacological properties of nutlins. We have demonstrated that the efficacy of the hit compound **2l** was comparable with Nutlin-3a in nanomolar concentrations in RKO cells. Moreover, this agent was able to stabilize p53 in SJSA-1 cell line characterized by an amplification of *MDM2* gene and subsequent overexpression of the protein. This fact indicated that the compound **2l** possessed essential biological activity. Thus, these agents and the synthetic strategy can be applied to a further design of new water-soluble nutlin family members.

## 4. Materials and Methods 

### 4.1. Chemistry

All solvents were used as received without further purification. The reactions were monitored by thin layer chromatography (TLC) carried out on Merck TLC silica gel plates (60 F254), using UV light for visualization and basic aqueous potassium permanganate or iodine fumes as developing agent. Flash column chromatography purifications were carried out using silica gel 60 (particle size 0.040–0.060 mm).

^1^H and ^13^C NMR spectra were recorded at 298 K on Bruker Avance 300 spectrometer with operating frequencies of 400 and 100 MHz, respectively, and calibrated using residual CHCl_3_ (δH = 7.26 ppm) and CDCl_3_ (δC = 77.16 ppm) or DMSO-d_5_ (δH = 2.50 ppm) and DMSO-d_6_ (δC = 39.52 ppm) as internal references. NMR data were presented as follows: chemical shift (δ ppm), multiplicity (s = singlet, d = doublet, dd = doublet of doublet, t = triplet, q = quartet, m = multiplet, br. = broad), coupling constant (J) in Hertz (Hz), integration.

High-resolution mass spectra (HRMS) were measured on a Thermo Scientific LTQ Orbitrap instrument using nanoelectrospray ionization (nano-ESI). Aromatic aldehydes were provided by Merk. The starting imidazolines were obtained according to the previously published procedure [17]. 

### 4.2. General Procedure for the Synthesis of Compounds ***2a**–**2aa***

An amount of 0.7656 g (2.58 mmol) of triphosgene was dissolved in CH_2_Cl_2_ (10 mL) and slowly added dropwise to a solution of an imidazoline derivative (1 mmol) and triethylamine 0.975 mL (7.01 mmol) in CH_2_Cl_2_ (10 mL) cooled to 0 °C. The reaction mixture was stirred for 30 min, then the solvent was removed under reduced pressure. The solid residue was dissolved in CH_2_Cl_2_ (10 mL), added dropwise to a solution of amine (19.4 mmol) in CH_2_Cl_2_ (10 mL) and stirred for 15 min. The reaction mixture was then washed with NaHCO_3_ solution followed by water and brine. The crude product was purified by column chromatography on silica gel using EtOAc/light petroleum ether (1:1) (or EtOAc/light petroleum ether (1:2) for 3,5-dimethoxy derivatives) as eluent.

***Cis*-*N****,**N*****-diethyl-2**,**4**,**5-*tris*(4-methoxyphenyl)-4**,**5-dihydro-*1H*-imidazole-1-carboxamide (2a).** White solid, 37% yield. Rf EtOAc/Et_3_N (99:1) 0.62. ^1^H NMR (CDCl_3_, 400 MHz, mixture of two conformers, single conformer was described): δ 0.89 (t, *J* = 5.9 Hz, 6H), 3.04–3.13 (m, 2H), 3.22–3.31 (m, 2H), 3.65 (s, 3H), 3.67 (s, 3H), 3.82 (s, 3H), 5.42 (d, *J* = 9.2 Hz, 1H), 5.59 (d, *J* = 9.2 Hz, 1H), 6.57 (d, *J* = 8.8 Hz, 2H), 6.62 (d, *J* = 8.8 Hz, 2H), 6.76 (d, *J* = 8.8 Hz, 2H), 6.85 (d, *J* = 8.6 Hz, 2H), 6.96 (d, *J* = 8.6 Hz, 2H), 7.83 (d, *J* = 8.8 Hz, 2H). ^13^C NMR (CDCl_3_, 100 MHz): 12.60, 41.10, 55.03, 55.07, 55.35, 70.40, 113.13, 113.89, 128.73, 128.80, 130.07, 158.51, 158.81, 162.11, 163.69. ESI-HRMS (*m*/*z*): calcd. for C_29_H_34_N_3_O_4_ [M + H]^+^: 488.2544; found: 488.2545.

**4-{[*Cis*-2**,**4**,**5-*tris*(4-methoxyphenyl)-4**,**5-dihydro-*1H*-imidazol-1-yl]carbonyl}morpholine (2b).** White solid, 40% yield. Rf EtOAc/Et_3_N (99:1) 0.50. ^1^H NMR (CDCl_3_, 400 MHz): δ 3.19–3.24 (m, 4H), 3.30–3.38 (m, 4H), 3.68 (s, 3H), 3.69 (s, 3H), 3.86 (s, 3H), 5.48 (d, *J* = 9.3 Hz, 1H), 5.52 (d, *J* = 9.3 Hz, 1H), 6.58 (d, *J* = 8.8 Hz, 2H), 6.62 (d, *J* = 8.7 Hz, 2H), 6.73 (d, *J* = 8.7 Hz, 2H), 6.83 (d, *J* = 8.8 Hz, 2H), 6.98 (d, *J* = 8.8 Hz, 2H), 7.76 (d, *J* = 8.8 Hz, 2H). ^13^C NMR (CDCl_3_, 100 MHz): 47.08, 54.96, 55.01, 55.32, 65.96, 66.45, 70.31, 113.13, 113.28, 113.92, 127.35, 128.47, 128.66, 129.05, 129.98, 155.40, 158.51, 158.82, 162.16, 162.76. ESI-HRMS (*m*/*z*): calcd. for C_29_H_32_N_3_O_5_ [M + H]^+^: 502.2336; found: 502.2342.

**Piperidin-1-yl(*cis*-2**,**4**,**5-*tris*(4-methoxyphenyl)-4**,**5-dihydro-*1H*-imidazol-1-yl)methanone (2c).** White solid, 42% yield. Rf EtOAc/Et_3_N (99:1) 0.60. ^1^H NMR (CDCl_3_, 400 MHz): δ 1.22–1.30 (m, 4H), 1.39–1.45 (m, 2H), 3.15–3.17 (m, 4H), 3.68 (s, 3H), 3.69 (s, 3H), 3.85 (s, 3H), 5.47 (d, *J* = 9.4 Hz, 1H), 5.52 (d, *J* = 9.4 Hz, 1H), 6.57 (d, *J* = 8.8 Hz, 2H), 6.62 (d, *J* = 8.8 Hz, 2H), 6.74 (d, *J* = 8.6 Hz, 2H), 6.84 (d, *J* = 8.8 Hz, 2H), 6.97 (d, *J* = 9.0 Hz, 2H), 7.79 (d, *J* = 8.8 Hz, 2H). ^13^C NMR (CDCl_3_, 100 MHz): 23.91, 25.21, 46.09, 55.01, 55.07, 55.37, 70.43, 71.38, 113.15, 113.22, 113.87, 127.70, 128.52, 128.74, 129.35, 130.07, 155.34, 158.51, 158.75, 162.08, 163.03. ESI-HRMS (*m*/*z*): calcd. for C_30_H_34_N_3_O_4_ [M + H]^+^: 500.2544; found: 500.2533.

**(4-Methylpiperidin-1-yl) (*cis*-2**,**4**,**5-*tris*(4-methoxyphenyl)-4**,**5-dihydro-*1H*-imidazol-1-yl)methanone (2d).** White solid, 75% yield. Rf EtOAc/Et_3_N (99:1) 0.75. ^1^H NMR (CDCl_3_, 400 MHz): δ 0.80 (d, *J* = 6.3 Hz, 3H), 1.31–1.39 (m, 1H), 1.39–1.49 (m, 2H), 2.38–2.55 (m, 2H), 3.68 (s, 3H), 3.70 (s, 3H), 3.86 (s, 3H), 3.74–3.94 (m, 4H), 5.49 (d, *J* = 9.4 Hz, 1H), 5.53 (d, *J* = 9.4 Hz, 1H), 6.58 (d, *J* = 8.7 Hz, 2H), 6.63 (d, *J* = 8.6 Hz, 2H), 6.74 (d, *J* = 8.6 Hz, 2H), 6.84 (d, *J* = 8.6 Hz, 2H), 6.97 (d, *J* = 8.8 Hz, 2H), 7.80 (d, *J* = 8.7 Hz, 2H). ^13^C NMR (CDCl_3_, 100 MHz): 21.50, 30.37, 33.19, 33.41, 45.23, 45.68, 55.03, 55.08, 55.40, 70.47, 113.20, 113.24, 113.96, 127.29, 128.63, 128.69, 129.02, 130.25, 154.88, 158.58, 158.84, 162.33, 163.23. ESI-HRMS (*m*/*z*): calcd. for C_31_H_36_N_3_O_4_ [M + H]^+^: 514.2700; found: 514.2686.

**Pyrrolidin-1-yl(*cis*-2**,**4**,**5-*tris*(4-methoxyphenyl)-4**,**5-dihydro-*1H*-imidazol-1-yl)methanone (2e).** White solid, 40% yield. Rf EtOAc/Et_3_N (99:1) 0.45. ^1^H NMR (CDCl_3_, 400 MHz, mixture of two conformers, single conformer was described): δ 1.62–1.70 (m, 4H), 3.09–3.24 (m, 4H), 3.68 (s, 3H), 3.69 (s, 3H), 3.86 (s, 3H), 5.47–5.56 (m, 2H), 6.57 (d, *J* = 8.8 Hz, 2H), 6.62 (d, *J* = 8.6 Hz, 2H), 6.74 (d, *J* = 8.2 Hz, 2H), 6.83 (d, *J* = 8.0 Hz, 2H), 6.97 (d, *J* = 9.0 Hz, 2H), 7.82–7.85 (m, 2H). ^13^C NMR (CDCl_3_, 100 MHz): 25.46, 47.83, 55.02, 55.08, 55.38, 69.73, 71.13, 113.18, 113.22, 113.94, 127.53, 128.44, 128.76, 130.07, 154.17, 158.60, 158.74, 162.35, 163.08. ESI-HRMS (*m*/*z*): calcd. for C_29_H_32_N_3_O_4_ [M + H]^+^: 486.2387; found: 486.2379.

**Azepan-1-yl(*cis*-2**,**4**,**5-*tris*(4-methoxyphenyl)-4**,**5-dihydro-*1H*-imidazol-1-yl)methanone (2f).** White solid, 41% yield. Rf EtOAc/Et_3_N (99:1) 0.60. ^1^H NMR (CDCl_3_, 400 MHz): δ 1.34–1.58 (m, 8H), 3.00–3.25 (m, 4H), 3.66 (s, 3H), 3.68 (s, 3H), 3.84 (s, 3H), 5.43 (d, *J* = 9.2 Hz, 1H), 5.51 (d, *J* = 9.2 Hz, 1H), 6.56 (d, *J* = 8.6 Hz, 2H), 6.62 (d, *J* = 8.6 Hz, 2H), 6.74 (d, *J* = 8.6 Hz, 2H), 6.83 (d, *J* = 8.6 Hz, 2H), 6.94 (d, *J* = 8.7, 2H), 7.78 (d, *J* = 8.7 Hz, 2H). ^13^C NMR (CDCl_3_, 100 MHz): 27.34, 27.89, 47.61, 55.01, 55.07, 55.35, 70.25, 71.53, 113.15, 113.20, 113.92, 127.57, 128.76, 128.82, 129.37, 129.95, 158.59, 158.79, 162.16, 163.50. ESI-HRMS (*m*/*z*): calcd. for C_31_H_36_N_3_O_4_ [M + H]^+^: 514.2700; found: 514.2689.

***tert*-Butyl 4-(*cis*-2**,**4**,**5-*tris*(4-methoxyphenyl)-4**,**5-dihydro-*1H*-imidazole-1-carbonyl)piperazine-1-carboxylate (2g).** White solid, 37% yield. Rf EtOAc/Et_3_N (99:1) 0.64. ^1^H NMR (CDCl_3_, 400 MHz): δ 1.40 (s, 9H), 3.07–3.15 (m, 4H), 3.17–3.24 (m, 4H), 3.68 (s, 3H), 3.70 (s, 3H), 3.86 (s, 3H), 5.53 (d, *J* = 9.5 Hz, 1H), 5.62 (d, *J* = 9.3 Hz, 1H), 6.57 (d, *J* = 8.6 Hz, 2H), 6.62 (d, *J* = 8.6 Hz, 2H), 6.73 (d, *J* = 8.4 Hz, 2H), 6.82 (d, *J* = 8.5 Hz, 2H), 6.97 (d, *J* = 8.7 Hz, 2H), 7.81 (d, *J* = 8.6 Hz, 2H). ^13^C NMR (CDCl_3_, 100 MHz): 28.23, 43.21, 44.99, 55.02, 55.06, 55.35, 70.41, 72.27, 80.23, 113.14, 113.34, 113.90, 127.83, 128.37, 128.75, 129.39, 129.86, 154.22, 156.04, 158.49, 158.78, 161.95, 162.40. ESI-HRMS (*m*/*z*): calcd. for C_34_H_41_N_4_O_6_ [M + H]^+^: 601.3021; found: 601.3014.

**Piperidin-1-yl(*cis*-2**,**4**,**5-*tris*(2-methoxyphenyl)-4**,**5-dihydro-*1H*-imidazol-1-yl)methanone** (**2h).** White solid, 37% yield. Rf EtOAc/Et_3_N (99:1) 0.68. ^1^H NMR (CDCl_3_, 400 MHz): δ 1.01–1.16 (m, 4H), 1.29–1.39 (m, 2H), 3.01–3.09 (m, 4H), 3.47 (s, 3H), 3.71 (s, 3H), 3.86 (s, 3H), 5.89 (d, *J* = 10.6 Hz, 1H), 6.22 (br. s, 1H), 6.44 (d, *J* = 8.1 Hz, 1H), 6.5 (br. s, 1H), 6.59 (br. s, 1H), 6.73 (br. s, 1H), 6.79 (t, *J* = 7.5 Hz, 1H), 6.93–6.98 (m, 2H), 7.01–7.07 (m, 2H), 7.31 (br. s, 1H), 7.44 (t, *J* = 8.2 Hz, 1H), 7.75 (d, *J* = 7.3 Hz, 1H). ^13^C NMR (CDCl_3_, 100 MHz): 23.95, 25.05, 46.41, 54.48, 55.02, 55.40, 64.89, 108.90, 109.35, 110.64, 119.01, 119.44, 120.53, 127.80, 128.12, 130.71, 158.92, 157.34. ESI-HRMS (*m*/*z*): calcd. for C_30_H_34_N_3_O_4_ [M + H]^+^: 500.2544; found: 500.2530.

***Cis*-*N****,**N*****-diethyl-2**,**4**,**5-*tris*(2**,**4-dimethoxyphenyl)-4**,**5-dihydro-*1H*-imidazole-1-carboxamide (2i).** White solid, 37% yield. Rf EtOAc/Et_3_N (99:1) 0.50. ^1^H NMR (CDCl_3_, 400 MHz): δ 0.75–0.86 (m, 6H), 2.91–3.00 (m, 2H), 3.26–3.35 (m, 4H), 3.54 (s, 3H), 3.59 (s, 3H), 3.65 (s, 3H), 3.69 (s, 3H), 3.81–3.83 (m, 6H), 5.76 (d, *J* = 10.0 Hz, 1H), 5.91 (d, *J* = 8.9 Hz, 1H), 6.08–6.14 (m, 3H), 6.24 (dd, *J* = 8.4 Hz, *J* = 2.0, 1H), 6.40–6.44 (m, 2H), 6.53 (dd, *J* = 8.5 Hz, *J* = 2.1 Hz, 1H), 6.83 (d, *J* = 8.7 Hz, 1H), 7.09 (br. s, 1H), 7.70 (d, *J* = 8.4 Hz, 1H). ^13^C NMR (CDCl_3_, 100 MHz): 0.94, 12.40, 40.73, 54.75, 54.91, 55.10, 55.17, 55.41, 55.47, 97.09, 97.20, 98.48, 103.16, 103.18, 104.76, 128.80, 131.95, 158.39, 159.66. ESI-HRMS (*m*/*z*): calcd. for C_32_H_40_N_3_O_7_ [M + H]^+^: 578.2861; found: 578.2867.

**4-{[*Cis*-2**,**4**,**5-*tris*(2**,**4-dimethoxyphenyl)-4**,**5-dihydro-*1H*-imidazol-1-yl]carbonyl}morpholine (2j).** White solid, 39% yield. Rf EtOAc/Et_3_N (99:1) 0.34. ^1^H NMR (CDCl_3_, 400 MHz): δ 3.07–3.17 (m, 4H), 3.22–3.29 (m, 4H), 3.48 (s, 3H), 3.64–3.68 (m, 6H), 3.48 (s, 3H), 3.66 (s, 3H), 3.70 (s, 3H), 3.81 (s, 3H), 3.84 (s, 3H), 5.73 (d, *J* = 10.1 Hz, 1H), 6.00 (br. s, 1H), 6.05–6.13 (m, 2H), 6.17 (br. s, 1H), 6.29 (dd, *J* = 8.4 Hz, *J* = 2.0 Hz, 1H), 6.47 (d, *J* = 2.1 Hz, 1H), 6.58 (dd, *J* = 8.5 Hz, *J* = 2.1 Hz, 1H), 6.68 (br. s, 1H), 7.11 (br. s, 1H), 7.72 (d, *J* = 8.4 Hz, 1H). ^13^C NMR (CDCl_3_, 100 MHz): 45.75, 54.65, 55.06, 55.15, 55.45, 66.08, 66.14, 97.18, 97.30, 98.40, 103.17, 103.22, 103.98, 104.22, 104.76, 128.47, 131.93, 158.59, 159.70. ESI-HRMS (*m*/*z*): calcd. for C_32_H_38_N_3_O_8_ [M + H]^+^: 592.2653; found: 592.2664.

**Piperidin-1-yl(*cis*-2**,**4**,**5-*tris*(2**,**4-dimethoxyphenyl)-4**,**5-dihydro-*1H*-imidazol-1-yl)methanone (2k).** White solid, 66% yield. Rf EtOAc/Et_3_N (99:1) 0.60. ^1^H NMR (CDCl_3_, 400 MHz): δ 1.08–1.20 (m, 4H), 1.29–1.37 (m, 2H), 2.99–3.08 (m, 4H), 3.46 (s, 3H), 3.64 (s, 6H), 3.68 (s, 3H), 3.79 (s, 3H), 3.82 (s, 3H), 5.70 (d, *J* = 10.2 Hz, 1H), 5.97 (br. s, 1H), 6.05 (d, *J* = 1.8 Hz, 1H), 6.14 (br. s, 1H), 6.27 (dd, *J* = 8.4 Hz, *J* = 2.0 Hz, 1H), 6.40–6.45 (m, 2H), 6.53 (dd, *J* = 8.4, *J* = 2.0, 1H), 6.67 (br. s, 1H), 7.10 (br. s, 1H), 7.67 (d, *J* = 8.2 Hz, 1H). ^13^C NMR (CDCl_3_, 100 MHz): 24.10, 25.21, 46.37, 45.80, 54.65, 55.06, 55.15, 55.33, 55.41, 65.09, 97.11, 97.21, 103.13, 104.49, 118.26, 120.04, 128.66, 131.69, 157.31, 157.75, 158.58, 159.51, 160.53, 162.48. ESI-HRMS (*m*/*z*): calcd. for C_33_H_40_N_3_O_7_ [M + H]^+^: 590.2861; found: 590.2875.

**(4-Methylpiperidin-1-yl)(*cis*-2**,**4**,**5-*tris*(2**,**4-methoxyphenyl)-4**,**5-dihydro-*1H*-imidazol-1-yl)methanone (2l).** White solid, 55% yield. Rf EtOAc/Et_3_N (99:1) 0.50. ^1^H NMR (CDCl_3_, 400 MHz): δ 0.50–0.66 (m, 2H), 0.75 (d, *J* = 6.4 Hz, 3H), 1.22–1.39 (m, 5H), 2.22–2.44 (m, 2H), 3.47 (s, 3H), 3.66 (s, 6H), 3.70 (s, 3H), 3.81 (s, 3H), 3.83 (s, 3H), 5.71 (d, *J* = 10.1 Hz, 1H), 6.00 (br. s, 1H), 6.07 (d, *J* = 2.2 Hz, 1H), 6.16 (br. s, 1H), 6.29 (dd, *J* = 8.4 Hz, *J* = 2.3 Hz, 1H), 6.36–6.46 (m, 3H), 6.55 (dd, *J* = 8.4, *J* = 2.3, 1H), 6.68 (br. s, 1H), 7.11 (br. s, 1H), 7.67 (d, *J* = 8.5 Hz, 1H). ^13^C NMR (CDCl_3_, 100 MHz): 21.69, 30.45, 33.10, 33.63, 45.66, 45.80, 54.65, 55.16, 55.24, 55.35, 55.43, 97.15, 97.22, 98.23, 103.15, 103.95, 104.07, 104.61, 128.62, 129.12, 131.78, 131.90, 158.64, 159.58. ESI-HRMS (*m*/*z*): calcd. for C_34_H_42_N_3_O_7_ [M + H]^+^: 604.3017; found: 604.3038.

***tert*-Butyl 4-(*cis*-2**,**4**,**5-*tris*(2**,**4-dimethoxyphenyl)-4**,**5-dihydro-*1H*-imidazole-1-carbonyl)piperazine-1-carboxylate (2m).** White solid, 20% yield. Rf EtOAc/Et_3_N (99:1) 0.45. ^1^H NMR (CDCl_3_, 400 MHz): δ 1.41 (s, 9H), 2.98–3.14 (m, 8H), 3.49 (s, 3H), 3.66 (s, 3H), 3.70 (s, 3H), 3.81 (s, 3H), 3.84 (s, 3H), 5.74 (d, *J* = 10.1 Hz, 1H), 6.08 (d, *J* = 1.8 Hz, 1H), 6.30 (d, *J* = 7.3 Hz, 1H), 6.45 (d, *J* = 2.1 Hz, 1H), 6.58 (d, *J* = 2.1 Hz, *J* = 8.4 Hz, 1H), 7.44–7.48 (m, 2H), 7.57–7.62 (m, 1H), 7.63–7.67 (m, 2H), 7.72–7.77 (m, 1H). ^13^C NMR (CDCl_3_, 100 MHz): 28.24, 42.92, 45.40, 54.67, 55.07, 55.16, 55.45, 80.08, 97.20, 98.39, 103.15, 103.23, 104.79, 112.32, 118.76, 129.03, 132.05, 132.69, 154.26, 158.54, 159.73. ESI-HRMS (*m*/*z*): calcd. for C_37_H_47_N_4_O_9_ [M + H]^+^: 691.3338; found: 691.3335.

***Cis*-*N****,**N*****-diethyl-2**,**4**,**5-*tris*(3**,**4-dimethoxyphenyl)-4**,**5-dihydro-*1H*-imidazole-1-carboxamide (2n).** White solid, 15% yield. Rf EtOAc/Et_3_N (99:1) 0.20. ^1^H NMR (CDCl_3_, 400 MHz): 0.92 (t, *J* = 7.1 Hz, 6H), 3.08–3.18 (m, 2H), 3.21–3.31 (m, 2H), 3.50 (s, 3H), 3.55 (s, 3H), 3.75 (s, 3H), 3.77 (s, 3H), 3.92 (s, 3H), 3.93 (s, 3H), 5.30 (d, *J* = 9.0 Hz, 1H), 5.57 (d, *J* = 9.1 Hz, 1H), 6.22 (d, *J* = 1.5 Hz, 1H), 6.32 (s, 1H), 6.56 (dd, *J* = 8.3 Hz, *J* = 1.7 Hz, 1H), 6.57–6.59 (m, 2H), 6.63–6.64 (m, 1H), 6.91 (d, *J* = 8.5 Hz, 1H), 7.38 (dd, *J* = 8.4 Hz, *J* = 2.0 Hz, 1H), 7.52 (d, *J* = 1.9, 1H). ^13^C NMR (CDCl_3_, 100 MHz): 12.70, 41.09, 55.44, 55.47, 55.60, 55.66, 55.84, 55.99, 70.41, 110.12, 110.19, 110.46, 110.75, 111.08, 119.96, 120.02, 121.37, 128.00, 129.82, 148.04, 148.19, 148.31, 148.83, 151.68, 164.05. ESI-HRMS (*m*/*z*): calcd. for C_32_H_40_N_3_O_7_ 578.2861; found: 578.2863.

**4-{[*Cis*-2**,**4**,**5-*tris*(3**,**4-dimethoxyphenyl)-4**,**5-dihydro-*1H*-imidazol-1-yl]carbonyl}morpholine (2o).** White solid, 33% yield. Rf EtOAc/Et_3_N (99:1) 0.15. ^1^H NMR (CDCl_3_, 400 MHz): 3.24–3.29 (m, 4H), 3.36–3.41 (m, 4H), 3.51 (s, 3H), 3.56 (s, 3H), 3.76 (s, 3H), 3.77 (s, 3H), 3.94 (s, 6H), 5.42 (d, *J* = 9.1 Hz, 1H), 5.54 (d, *J* = 9.1 Hz, 1H), 6.20 (d, *J* = 1.7 Hz, 1H), 6.31 (d, *J* = 1.6 Hz, 1H), 6.55 (dd, *J* = 8.3 Hz, *J* = 1.7 Hz, 1H), 6.60–6.67 (m, 3H), 6.93 (d, *J* = 8.4 Hz, 1H), 7.35 (dd, *J* = 8.4 Hz, *J* = 1.9 Hz, 1H), 7.49 (d, *J* = 1.9 Hz, 1H). ^13^C NMR (CDCl_3_, 100 MHz): 45.47, 55.53, 55.65, 55.72, 55.92, 56.08, 66.15, 70.54, 110.30, 110.48, 110.53, 111.04, 111.16, 119.85, 119.91, 121.33, 127.94, 129.65, 148.16, 148.41, 148.43, 148.48, 148.97, 151.81, 163.19. ESI-HRMS (*m*/*z*): calcd. for C_32_H_38_N_3_O_8_ 592.2653; found: 592.2657.

**Piperidin-1-yl(*cis*-2**,**4**,**5-*tris*(3**,**4-dimethoxyphenyl)-4**,**5-dihydro-*1H*-imidazol-1-yl)methanone (2p).** White solid, 14% yield. Rf EtOAc/Et_3_N (99:1) 0.20. ^1^H NMR (CDCl_3_, 400 MHz): 1.30 (br. s, 4H), 1.39–1.48 (m, 2H), 3.20 (br. s, 4H), 3.51 (s, 3H), 3.56 (s, 3H), 3.76 (s, 3H), 3.78 (s, 3H), 3.94 (s, 3H), 3.97 (s, 3H), 5.60 (br. s, 2H), 6.23 (s, 1H), 6.33 (d, *J* = 1.2 Hz, 1H), 6.57–6.61 (m, 2H), 6.64–6.67 (m, 2H), 6.94 (d, *J* = 8.5 Hz, 1H), 7.42 (dd, *J* = 8.5 Hz, *J* = 1.9 Hz, 1H), 7.57 (s, 1H). ^13^C NMR (CDCl_3_, 100 MHz): 23.76, 25.25, 46.01, 55.45, 55.48, 55.58, 55.66, 55.89, 56.09, 70.60, 110.17, 110.31, 110.55, 110.70, 111.05, 111.24, 119.73, 120.23, 121.81, 129.23, 148.13, 148.23, 148.34, 148.42, 148.84, 152.08, 163.78. ESI-HRMS (*m*/*z*): calcd. for C_33_H_40_N_3_O_7_ 590.2861; found: 590.2862.

**(4-Methylpiperidin-1-yl)(*cis*-2**,**4**,**5-*tris*(3**,**4-dimethoxyphenyl)-4**,**5-dihydro-*1H*-imidazol-1-yl)methanone (2q).** White solid, 30% yield. Rf EtOAc/Et_3_N (99:1) 0.25. ^1^H NMR (CDCl_3_, 400 MHz): 0.78 (d, *J* = 6.4 Hz, 3H), 1.31–1.39 (m, 1H), 1.39–1.49 (m, 2H), 2.40–2.54 (m, 2H), 3.49 (s, 3H), 3.56 (s, 3H), 3.74 (s, 3H), 3.75 (s, 3H), 3.78–3.90 (m, 4H), 3.91 (s, 6H), 5.38 (d, *J* = 9.1 Hz, 1H), 5.49 (d, *J* = 9.1 Hz, 1H), 6.19 (d, *J* = 1.5 Hz, 1H), 6.33 (d, *J* = 1.5 Hz, 1H), 6.54 (dd, *J* = 8.3 Hz, *J* = 1.7 Hz, 1H), 6.58 (s, 1H), 6.58–6.60 (m, 2H), 6.64 (d, *J* = 8.3 Hz, 1H), 6.91 (d, *J* = 8.4 Hz, 1H), 7.35 (dd, *J* = 8.3 Hz, *J* = 1.8 Hz, 1H), 7.43 (d, *J* = 1.7 Hz, 1H). ^13^C NMR (CDCl_3_, 100 MHz): 21.50, 30.36, 33.36, 33.48, 45.16, 45.57, 55.43, 55.45, 55.58, 55.66, 55.86, 55.97, 70.54, 72.58, 110.21, 110.42, 110.47, 110.93, 111.09, 119.86, 119.95, 121.26, 122.69, 128.26, 129.94, 147.98, 148.24, 148.28, 148.79, 151.47, 155.92, 163.26. ESI-HRMS (*m*/*z*): calcd. for C_34_H_42_N_3_O_7_ [M + H]^+^: 604.3017; found 604.3010.

**Pyrrolidin-1-yl(*cis*-2**,**4**,**5-*tris*(3**,**4-dimethoxyphenyl)-4**,**5-dihydro-*1H*-imidazol-1-yl)methanone (2r).** White solid, 40% yield. Rf EtOAc/Et_3_N (99:1) 0.20. ^1^H NMR (CDCl_3_, 400 MHz): 1.68 (br. s, 4H), 3.20 (br. s, 4H), 3.51 (s, 3H), 3.54 (s, 3H), 3.75 (s, 3H), 3.77 (s, 3H), 3.93 (s, 3H), 3.95 (s, 3H), 5.50 (d, *J* = 8.8 Hz, 1H), 5.57 (d, *J* = 8.9 Hz, 1H), 6.23 (d, *J* = 1.2 Hz, 1H), 6.29 (d, *J* = 1.1 Hz, 1H), 6.55–6.69 (m, 4H), 6.92 (d, *J* = 8.4 Hz, 1H), 6.96 (s, 1H), 7.41 (dd, *J* = 8.3 Hz, *J* = 1.8 Hz, 1H), 7.57 (s, 1H). ^13^C NMR (CDCl_3_, 100 MHz): 30.20, 34.10, 47.40, 55.51, 55.64, 55.73, 55.91, 56.13, 56.14, 69.80, 110.30, 110.52, 111.13, 111.21, 119.76, 119.95, 121.41, 125.38, 128.11, 135.66, 148.15, 148.26, 148.28, 148.41, 148.91, 151.38, 163.23. ESI-HRMS (*m*/*z*): calcd. for C_32_H_38_N_3_O_7_ [M + H]^+^: 576.2704; found 576.2701.

**Azepan-1-yl(*cis*-2**,**4**,**5-*tris*(3**,**4-dimethoxyphenyl)-4**,**5-dihydro-*1H*-imidazol-1-yl)methanone (2s).** White solid, 35% yield. Rf EtOAc/Et_3_N (99:1) 0.25. ^1^H NMR (CDCl_3_, 400 MHz): 1.35–1.62 (m, 8H), 3.07–3.34 (m, 4H), 3.49 (s, 3H), 3.56 (s, 3H), 3.75 (s, 3H), 3.76 (s, 3H), 3.92 (s, 3H), 3.93 (s, 3H), 5.43 (d, *J* = 9.1 Hz, 1H), 5.55 (d, *J* = 9.1 Hz, 1H), 6.21 (s, 1H), 6.34 (d, *J* = 1.4 Hz, 1H), 6.55–6.67 (m, 4H), 6.90 (d, *J* = 8.4 Hz, 1H), 7.39 (dd, *J* = 8.3 Hz, *J* = 1.9 Hz, 1H), 7.50 (d, *J* = 1.5 Hz, 1H). ^13^C NMR (CDCl_3_, 100 MHz): 27.36, 27.92, 47.64, 55.48, 55.61, 55.70, 55.89, 56.05, 70.32, 110.12, 110.27, 110.48, 110.73, 110.98, 111.09, 120.02, 121.34, 129.86, 148.07, 148.16, 148.26, 148.35, 148.85, 151.71, 163.79. ESI-HRMS (*m*/*z*): calcd. for C_34_H_42_N_3_O_7_ [M + H]^+^: 604.3017; found 604.3023.

**(4-Methylpiperazin-1-yl)(*cis*-2**,**4**,**5-*tris*(3**,**4-dimethoxyphenyl)-4**,**5-dihydro-*1H*-imidazol-1-yl)methanone (2t).** White solid, 29% yield. Rf EtOAc/Et_3_N (99:1) 0.03. ^1^H NMR (CDCl_3_, 400 MHz): 2.10–2.16 (br. s, 3H), 2.52–2.60 (m, 4H), 3.24–3.32 (m, 4H), 3.50 (s, 3H), 3.56 (s, 3H), 3.75 (s, 3H), 3.76 (s, 3H), 3.91 (s, 3H), 3.92 (s, 3H), 5.30 (d, *J* = 8.8 Hz, 1H), 5.50 (d, *J* = 8.8 Hz, 1H), 6.18 (d, *J* = 2.0 Hz, 1H), 6.34 (d, *J* = 1.8 Hz, 1H), 6.54 (dd, *J* = 8.4 Hz, *J* = 2.0 Hz, 1H), 6.58–6.62 (m, 2H), 6.64 (d, *J* = 8.2 Hz, 1H), 6.91 (d, *J* = 8.4, 1H), 7.34 (dd, *J* = 8.2 Hz, *J* = 2.0 Hz, 1H), 7.43 (d, *J* = 2.0 Hz, 1H). ^13^C NMR (CDCl_3_, 100 MHz): 44.52, 45.53, 54.10, 55.55, 55.53, 55.64, 55.71, 55.88, 55.99, 70.51, 73.91, 110.27, 110.36, 110.38, 110.52, 110.86, 111.22, 119.60, 120.07, 120.96, 123.36, 128.62, 130.14, 148.02, 148.31, 148.35, 148.40, 148.91, 151.40, 156.70, 162.77. ESI-HRMS (*m*/*z*): calcd. for C_33_H_41_N_4_O_7_ [M + H]^+^: 605.2970; found 605.2983.

***Cis*-*N****,**N*****-diethyl-2**,**4**,**5-*tris*(3**,**5-dimethoxyphenyl)-4**,**5-dihydro-*1H*-imidazole-1-carboxamide (2u).** White solid, 47% yield. Rf EtOAc/Et_3_N (99:1) 0.75. ^1^H NMR (CDCl_3_, 400 MHz): δ 0.94 (t, *J* = 7.0 Hz, 6H), 3.10–3.20 (m, 2H), 3.26–3.37 (m, 2H), 3.56 (s, 6H), 3.58 (s, 6H), 3.84 (s, 6H), 5.44 (br. s, 1H), 5.65 (d, *J* = 8.8 Hz, 1H), 6.09 (s, 2H), 6.13–6.15 (m, 2H), 6.17–6.20 (m, 2H), 6.61 (d, *J* = 2.0 Hz, 1H), 7.05 (s, 2H). ^13^C NMR (CDCl_3_, 100 MHz): 12.70, 14.13, 21.00, 41.22, 55.15, 55.57, 60.33, 70.61, 99.81, 100.04, 104.13, 105.58, 105.84, 106.23, 139.21, 155.57, 160.20, 160.21, 160.70, 164.88. ESI-HRMS (*m*/*z*): calcd. for C_32_H_40_N_3_O_7_ [M + H]^+^: 578.2861; found 578.2863.

**4-{[*Cis*-2**,**4**,**5-*tris*(3**,**5-dimethoxyphenyl)-4**,**5-dihydro-*1H*-imidazol-1-yl]carbonyl}morpholine (2v).** White solid, 40% yield. Rf EtOAc/Et_3_N (99:1) 0.55. ^1^H NMR (CDCl_3_, 400 MHz): δ 3.27–3.33 (m, 4H), 3.37–3.44 (m, 4H), 3.56 (s, 6H), 3.59 (s, 6H), 3.83 (s, 6H), 5.42 (d, *J* = 9.4 Hz, 1H), 5.55 (d, *J* = 9.4 Hz, 1H), 6.04 (d, *J* = 2.2 Hz, 2H), 6.12 (d, *J* = 2.2 Hz, 2H), 6.18–6.22 (m, 2H), 6.61–6.63 (m, 1H), 6.97–6.99 (m, 2H). ^13^C NMR (CDCl_3_, 100 MHz): 45.55, 55.10, 55.14, 55.53, 66.11, 70.59, 99.56, 99.86, 103.42, 105.27, 105.89, 106.17, 138.15, 139.39, 156.11, 160.18, 160.36, 160.75, 163.29. ESI-HRMS (*m*/*z*): calcd. for C_32_H_38_N_3_O_8_ [M + H]^+^: 592.2653; found 592.2665.

**(4-Methylpiperazin-1-yl)(*cis*-2**,**4**,**5-*tris*(3**,**5-dimethoxyphenyl)-4**,**5-dihydro-*1H*-imidazol-1-yl)methanone (2w).** White solid, 26% yield. Rf EtOAc/Et_3_N (99:1) 0.60. ^1^H NMR (CDCl_3_, 400 MHz): δ 2.16–2.28 (m, 7H), 3.31–3.42 (m, 4H), 3.56 (s, 6H), 3.58 (s, 6H), 3.82 (s, 6H), 5.33 (d, *J* = 9.1 Hz, 1H), 5.53 (d, *J* = 9.1 Hz, 1H), 6.04 (d, *J* = 2.3 Hz, 2H), 6.12 (d, *J* = 2.3 Hz, 2H), 6.17 (t, *J* = 2.3 Hz, 1H), 6.19 (t, *J* = 2.3 Hz, 1H), 6.59 (t, *J* = 2.3 Hz, 1H), 6.96 (d, *J* = 2.3 Hz, 2H). ^13^C NMR (CDCl_3_, 100 MHz): 44.56, 45.53, 54.02, 55.14, 55.52, 70.58, 74.32, 99.53, 99.74, 103.16, 105.12, 105.91, 106.03, 132.64, 138.60, 139.70, 156.49, 160.15, 160.36, 160.73, 163.09. ESI-HRMS (*m*/*z*): calcd. for C_33_H_41_N_4_O_7_ [M + H]^+^: 605.2970; found 605.2982.

**Piperidin-1-yl(*cis*-2**,**4**,**5-*tris*(4-chlorophenyl)-4**,**5-dihydro-*1H*-imidazol-1-yl)methanone (2x).** White solid, 37% yield. Rf EtOAc/Et_3_N (99:1) 0.82. ^1^H NMR (DMSO-d_6_, 400 MHz): δ 1.04–1.33 (m, 4H), 1.35–1.52 (m, 2H), 3.12–3.43 (m, 4H), 6.10 (d, *J* = 11.4 Hz, 1H), 6.31 (d, *J* = 11.2 Hz, 1H), 7.09 (d, *J* = 7.9 Hz, 2H), 7.22 (d, *J* = 7.8 Hz, 2H), 7.24–7.34 (m, 4H), 7.80 (d, *J* = 7.8 Hz, 2H), 7.94 (d, *J* = 8.0 Hz, 2H). ^13^C NMR (DMSO-d_6_, 400 MHz): 13.05, 23.14, 24.94, 45.79, 69.29, 68.91, 127.99, 129.15, 129.48, 130.20, 131.12, 130.80, 132.55, 133.15, 133.44, 138.90, 149.74, 165.98. ESI-HRMS (*m*/*z*): calcd. for C_27_H_25_Cl_3_N_3_O [M + H]^+^: 512.1058; found 512.1043. 

***Cis*-*N****,**N*****-diethyl-2**,**4**,**5-*tris*(2**,**4-dichlorophenyl)-4**,**5-dihydro-*1H*-imidazole-1-carboxamide (2y).** White solid, 56% yield. Rf EtOAc/Et_3_N (99:1) 0.90. ^1^H NMR (CDCl_3_, 400 MHz): 0.84 (t, *J* = 7.0 Hz, 6H), 2.94–3.07 (m, 2H), 3.37–3.50 (m, 2H), 6.11 (d, *J* = 10.6 Hz, 1H), 6.27 (d, *J* = 10.6 Hz, 1H), 6.99 (dd, *J* = 8.4 Hz, *J* = 1.9 Hz, 1H), 7.06 (d, *J* = 8.4 Hz, 1H), 7.10 (dd, *J* = 8.4 Hz, *J* = 1.7 Hz, 1H), 7.19 (d, *J* = 1.7 Hz, 2H), 7.28–7.35 (m, 2H), 7.48 (d, *J* = 1.7Hz, 1H), 7.64 (d, *J* = 8.3 Hz, 1H). 13C NMR (CDCl_3_, 100 MHz): 12.48, 41.14, 63.98, 69.81, 126.56, 126.66, 127.27, 128.68, 129.19, 129.89, 130.30, 130.71, 131.20, 132.68, 133.97, 134.32, 136.62, 162.01. ESI-HRMS (*m*/*z*): calcd. for C_26_H_22_Cl_6_N_3_O [M + H]^+^: 601.9889; found 601.9878.

**4-{[*Cis*-2**,**4**,**5-*tris*(2**,**4-dichlorophenyl)-4**,**5-dihydro-*1H*-imidazol-1-yl]carbonyl}morpholine (2z).** White solid, 40% yield. Rf EtOAc/Et_3_N (99:1) 0.90. ^1^H NMR (CDCl_3_, 400 MHz): 3.13–3.34 (m, 4H), 3.36–3.51 (m, 4H), 6.11 (d, *J* = 10.6 Hz, 1H), 6.31 (d, *J* = 10.6 Hz, 1H), 6.93–6.99 (m, 2H), 7.14 (d, *J* = 8.0 Hz, 1H), 7.19 (d, *J* = 1.8 Hz, 1H), 7.24 (d, *J* = 1.8 Hz, 1H), 7.28–7.39 (m, 2H), 7.51 (d, *J* = 1.2 Hz, 1H), 7.74 (d, *J* = 8.0 Hz, 1H). ^13^C NMR (CDCl_3_, 100 MHz):, 45.78, 47.57, 63.85, 66.23, 66.64, 126.70, 127.49, 128.89, 129.46, 130.07, 130.44, 133.89, 134.35. ESI-HRMS (*m*/*z*): calcd. for C_26_H_20_Cl_6_N_3_O_2_ [M + H]^+^: 615.9681; found 615.9669.

***tert*-Butyl 4-(*cis*-2**,**4**,**5-*tris*(2**,**4-dichlorophenyl)-4**,**5-dihydro-*1H*-imidazole-1-carbonyl)piperazine-1-carboxylate (2aa).** White solid, 70% yield. Rf EtOAc/Et_3_N (99:1) 0.90. ^1^H NMR (CDCl_3_, 400 MHz): 1.43 (s, 9H), 3.08–3.28 (m, 8H), 6.10 (d, *J* = 10.6 Hz, 1H), 6.26 (d, *J* = 10.6 Hz, 1H), 6.93–6.96 (m, 1H), 7.10 (m, 1H), 7.18 (d, *J* = 1.8 Hz, 1H), 7.21 (d, *J* = 1.0 Hz, 1H), 7.24–7.29 (m, 1H), 7.46–7.50 (m, 2H), 7.58–7.68 (m, 1H), 7.69 (d, J = 8.4, 1H). ^13^C NMR (CDCl_3_, 100 MHz): 30.89, 45.78, 47.57 63.85, 66.23, 66.64, 80.06, 126.70, 126.83, 127.49, 128.89, 129.46, 130.07, 130.44, 133.89, 134.35. ESI-HRMS (*m*/*z*): calcd. for C_31_H_29_Cl_6_N_4_O_3_ [M + H]^+^: 715.0365; found 715.0353.

### 4.3. In Silico Studies

The calculation was conducted using Schrodinger software suite. The presented results were obtained using «Induced Fit» (Glide + PrimeX modules) scoring method, which allowed us to refine docking poses previously obtained by screening in the Glide module alone. Molecular dynamics (MD) solvation/stability testing was conducted using the Desmond module. In all in silico studies, OPLS3e force field was used for docking, and in MD simulations, TIP4PD potential was used for interaction with water molecules. The trajectory in MD simulations was 5 ns. Maximum distance, where direct water (MD) and aminoacid (PrimeX) interactions with the ligand were considered significant, was set to 10 Å.

### 4.4. Cell Culture and Experimental Procedures

The osteosarcoma cell line SJSA-1, prostate cell line LNCaP, colon carcinoma cell lines RKO and HCT116, and neuroblastoma cell lines SH-SY5Y and SK-N-SH were used in this study. Cells were grown in a CO_2_ incubator (5% CO_2_) in DMEM high glucose or RPMI1640 medium containing 4.5 g/L glucose (Gibco), with 10% FBS (Gibco) at 37 °C, in the presence of a mixture of antibiotics and antimycotics (Gibco). The cells in the logarithmic growth phase were used for experiments. Before the treatment with synthesized derivatives or Nutlin-3a (Sigma) and RG7388 (Roche), the culture medium was removed, and cells were washed with PBS. Fresh medium containing the above-noted agents in the selected concentration was added to the cells. 

### 4.5. Gel Electrophoresis and Western Blot (WB) Analysis

After the indicated time of treatment, cells were removed from culture dishes using a cell scraper. Next, cells were centrifuged (500 rcf, 5 min, 4 °C) and washed with ice-cold PBS (Paneco). Then, the cell pellet was lysed in RIPA buffer (25 mM Tris-HCl (pH 7.4), 150 mM NaCl, 0.1% SDS, 0.5% sodium deoxycholate, 1% NP-40, cOmplete™ Protease Inhibitor Cocktail (Roche)) for 20 min on ice. Lysates were centrifuged (16,000 rcf, 20 min, 4 °C), and a part of the supernatant was taken for the protein concentration assay, and another part was used for Western Blot analysis, as previously described [14]. The densitometric analysis was performed using Image Lab Software or ImageJ 4.1.

The following primary antibodies were used for WB: anti-GAPDH (#2118), anti-p21 (#2947), anti-Puma (#4976) (all from Cell Signaling Technology), anti-PARP (#137653) (Abcam) and anti-p53 (012M4795) (Sigma) antibodies. HRP-linked goat anti-mouse and anti-rabbit antibodies (#97046 and #97200, respectively; both from Abcam) were used as secondary antibodies.

### 4.6. Fluorescence-Activated Cell Sorting Analysis (FACS-Analysis)

After 24 h incubation with all agents, cells were removed from the culture dishes using 0.05% trypsin-EDTA (Gibco) and transferred to a conditioned medium. A total of 10^5^ cells were taken for analysis, centrifuged (1000 rcf, 4 min, +4 °C), washed with PBS solution, and centrifuged again (1000 rcf, 4 min, +4 °C). The cell pellet was resuspended in 200 µL Annexin-binding buffer (BD Biosciences) and 2 μL of Annexin V-FITC (Invitrogen) were added. Then samples were incubated in the dark at room temperature for 15 min. Immediately before the measurement, 5 μL of propidium iodide (50 μg/mL) (BD Biosciences) were added to each sample, and the samples were analyzed by the BD FACSCanto II cell analyzer (BD Biosciences). Flow cytometry data were processed using BD FACSDiva software 7.0 (BD Biosciences).

### 4.7. Sub-G1 Test

After the indicated time of treatment, cells were collected as described above and fixed in 70% ethanol for 1 h at −20 °C. Then, cells were washed of ethanol and re-suspended in PBS, supplemented with 1% RNase A and stained with 20 μg/mL propidium iodide for 15 min at 37 °C. After staining, cells were examined using the FACSCanto II cell analyzer (BD Biosciences). Flow cytometry data were processed using BD FACSDiva software 7.0 (BD Biosciences).

### 4.8. Cytotoxic Assay

The half maximal inhibitory concentration (IC50) of the synthesized compounds against several human cell lines was determined by the MTT method. Cells were cultured at 1.0 × 10^4^ cells/200 μL in 96-well plates in DMEM or RPMI1640 medium. After 24 h, the various concentrations of the tested compounds (1.56–100 μM) were added to each well, and cells were incubated for 72 h. All compounds were dissolved in DMSO. The final DMSO concentration in each well did not exceed 0.1% and was not toxic for the cells. After incubation, 20 μL MTT reagent [3-(4,5-dimethylthiazol-2-yl)-2,5-diphenyltetrazolium bromide, 5 mg/mL] was added to each well, and the cells were incubated for 2 h. The medium was removed, and 100 μL DMSO was added to each well. The optical density was measured at 530 nm using the Victor3 (PerkinElmer) microplate reader. Concentrations (IC50) were calculated according to the dose-dependent inhibition curves using OriginPro 9.0 software. The experiments were carried out in triplicate.

### 4.9. Statistical Analysis

The data were presented as mean ± SD values from at least three independent experiments. For the statistical analysis, all data were tested for homogeneity of variance and normality using Levene’s and Shapiro–Wilk tests, respectively. For normally distributed data, a Student’s t-test was performed to analyze statistically significant differences between groups. The value representing of *p* < 0.05 was considered statistically significant. The graphs were performed using Microsoft Excel software.

## 5. Conclusions

In this study we have performed a two-step synthetic approach to obtain imidazoline-based alkoxyaryl derivatives. These compounds are able to block p53-MDM2 PPIs. The treatment of several cancer cell lines with them led to accumulation of p53 and p53-inducible proteins. Suggested synthetic strategy can be used for design of new anti-cancer agents in future.

## Figures and Tables

**Table 1:** Synthesized 2,4,5-triaryl cis-imidazoline derivatives.

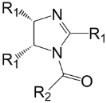							
ID	R1	R2	Yield, %	ID	R1	R2	Yield, %
**2a**	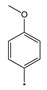	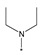	37	**2o**	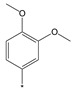	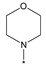	33
**2b**	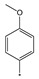	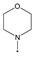	40	**2p**	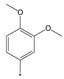	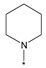	14
**2c**	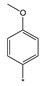	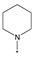	42	**2q**	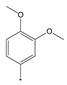	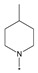	30
**2d**	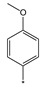	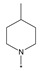	75	**2r**	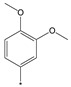	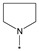	40
**2e**	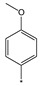	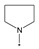	40	**2s**	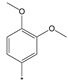	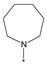	35
**2f**	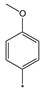	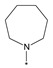	41	**2t**	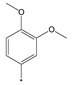	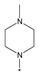	29
**2g**	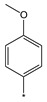	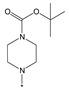	38	**2u**	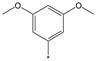	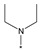	47
**2h**	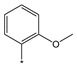	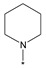	37	**2v**	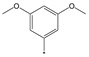	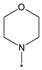	40
**2i**	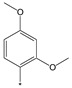	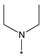	37	**2w**	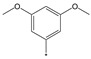	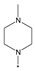	26
**2j**	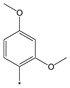	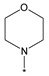	39	**2x**	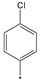	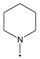	37
**2k**	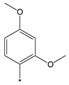	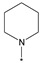	66	**2y**	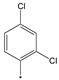	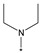	56
**2l**	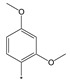	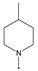	55	**2z**	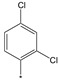	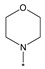	40
**2m**	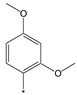	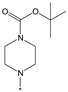	20	**2aa**	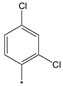	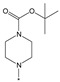	70
**2n**	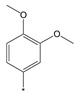	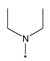	15				

## Data Availability

Data is contained within the article and Supplementary Material.

## Supplementary Materials

The following supporting information can be downloaded at: https://www.mdpi.com/article/10.3390/ph15040444/s1. Figure S1: The docked pose (on the left) for **2m** (green) in p53 binding site of MDM2 protein, in comparison to nutlin-3a (grey), as well as its ligand-protein interaction map (on the right). Figure S2: The docked pose (on the left) for **2n** (green) in p53 binding site of MDM2 protein, in comparison to nutlin-3a (grey), as well as its ligand-protein interaction map (on the right). Figure S3: The docked pose (on the left) for **2i** (green) in p53 binding site of MDM2 protein, in comparison to nutlin-3a (grey), as well as its ligand-protein interaction map (on the right). Figure S4: A. Western Blot analysis of total cellular lysates from RKO cells upon treatment with RG7388 and RG7112. B. The histograms of flow cytometry (FC) analysis data for RKO cells using Annexin V-FITC/PI staining. Figure S5: A, C. Western Blot analysis of total cellular lysates from SJSA-1 (A) and LNCaP (C) cells upon treatment with compounds **2l**, **2k**, Nutlin-3a and RG7388. B, D—Densitometric analysis of p53, p21 and Puma bands normalized to GAPDH in SJSA-1 (B) and LNCaP (D) cells. Table S1: Cytotoxicity of synthesized 2,4,5-triaryl cis-imidazoline derivatives to HCT-116 cell line.
