# Supplementary material for: Synthetic Design and Biological Evaluation of New p53-MDM2 Interaction Inhibitors Based on Imidazoline Core
Source: Pharmaceuticals (Basel). 2022 Apr 2;15(4):444. doi: 10.3390/ph15040444 (PMC9027661; doi:10.3390/ph15040444)

Supplementary Materials

Daniil R. Bazanov [1,†], Nikolay V. Pervushin [2,†], Egor V. Savin [2], Michael D. Tsymliakov [1], Anita I. Maksutova [1], Victoria Yu. Savitskaya [1], Sergey E. Sosonyuk [1], Yulia A. Gracheva [1], Michael Yu. Seliverstov [1], Natalia A. Lozinskaya[† 1*] and Gelina S. Kopeina[† 2*].

[1] Department of Chemistry, M. V. Lomonosov Moscow State University, 1, Leninskie Gory, 119992 Moscow, Russian Federation
[2] Department of Medicine, M. V. Lomonosov Moscow State University, 1, Leninskie Gory, 119991 Moscow, Russian Federation
[†] and [‡] these authors contributed equally to this work
🖂 Gelina S. Kopeina lirroster@gmail.com Natalia A. Lozinskaya natalylozinskaya@mail.ru

Synthetic design and biological evaluation of new p53-MDM2 interaction inhibitors based on imidazoline core.

Pharmaceuticals 2022, 15, 444. https://doi.org/10.3390/ph15040444

Molecular docking

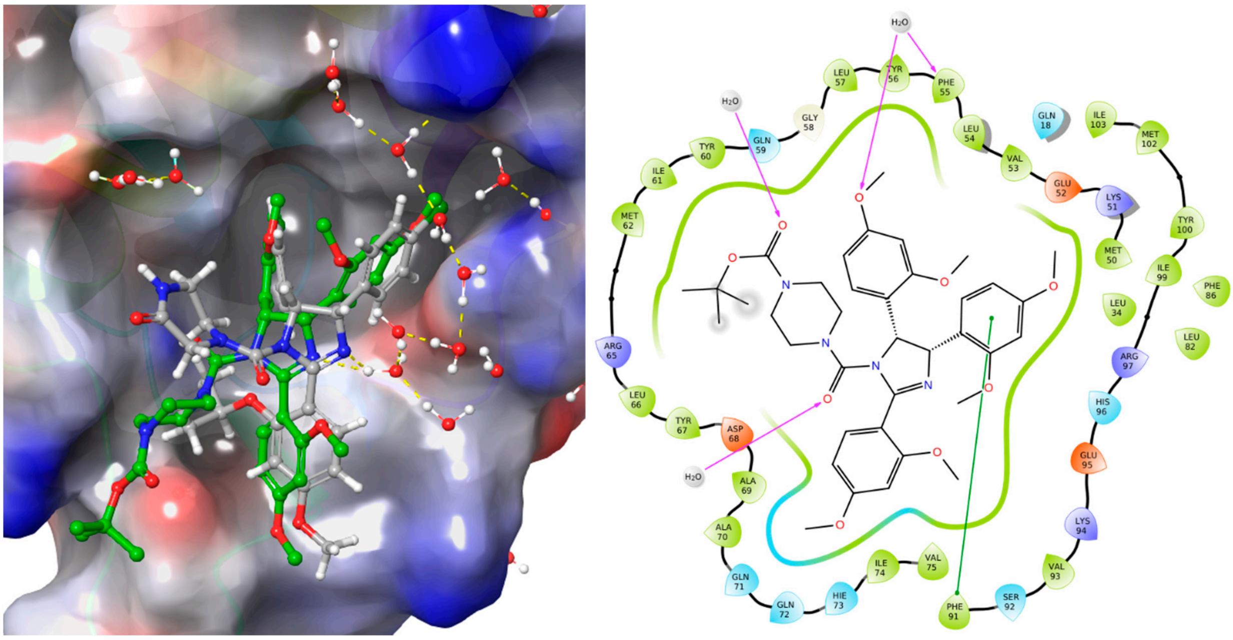

Supplementary Figure S1 The docked pose (on the left) for **2m** (green) in p53 binding site of MDM2 protein in comparison to nutlin-3a (grey), as well as its ligand-protein interaction map (on the right)

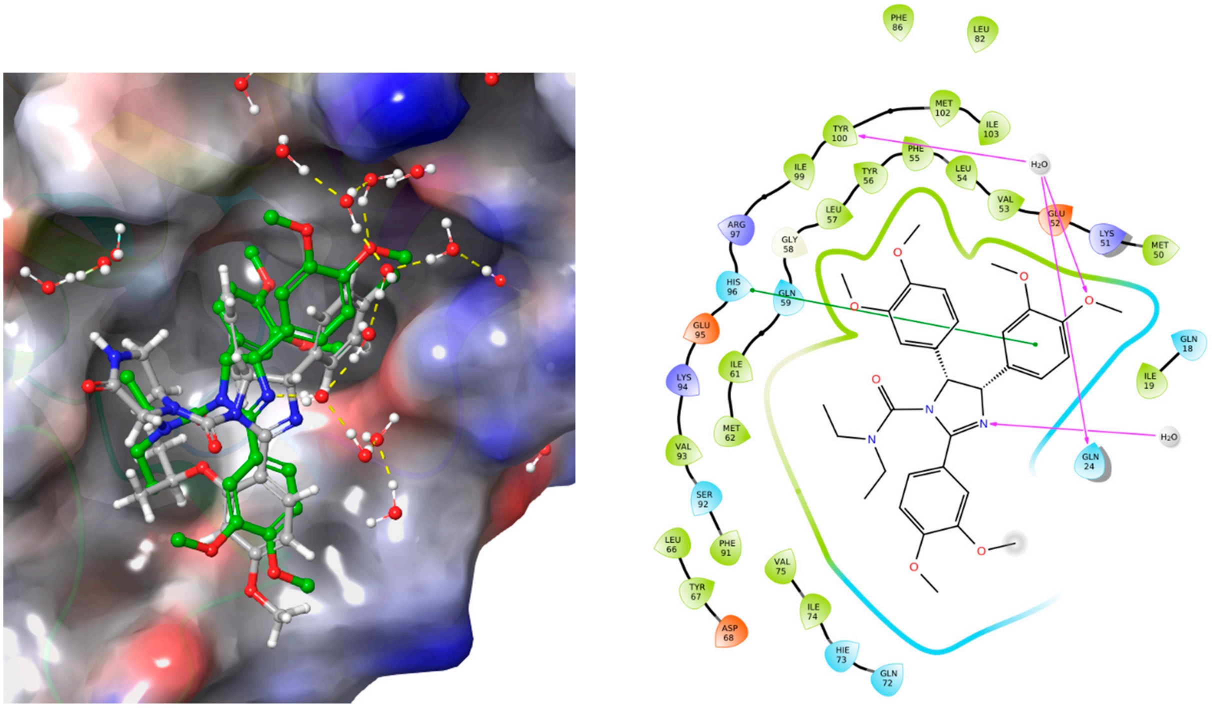

Supplementary Figure S2 The docked pose (on the left) for **2n** (green) in p53 binding site of MDM2 protein in comparison to nutlin-3a (grey), as well as its ligand-protein interaction map (on the right).

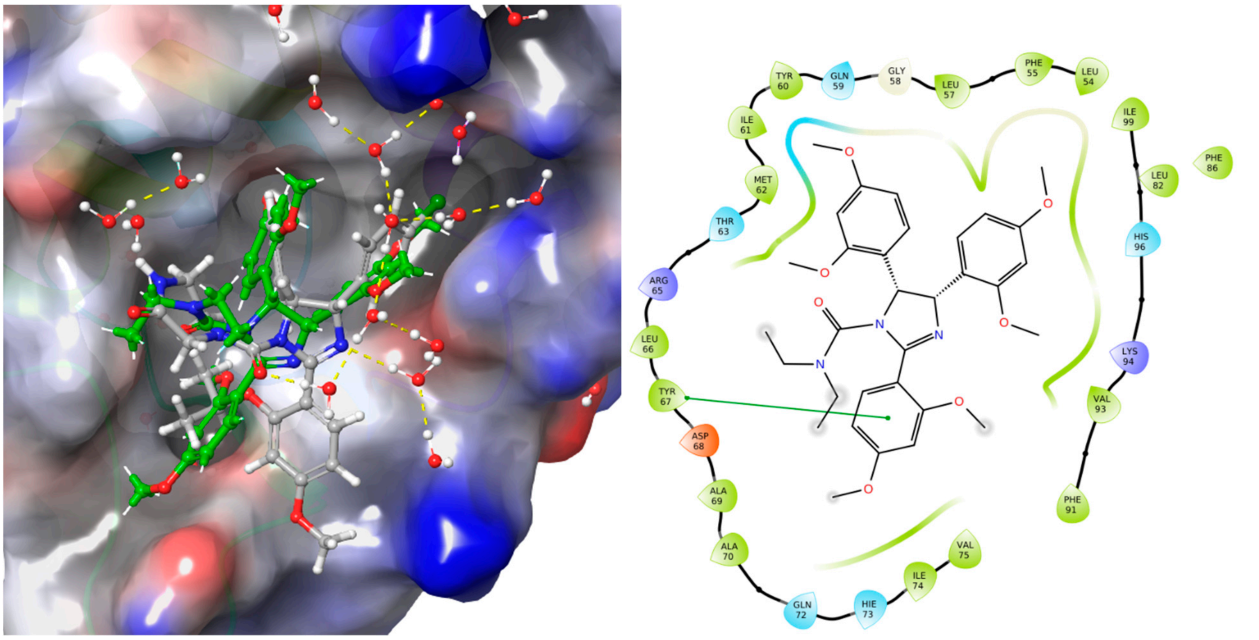

Supplementary Figure S3 The docked pose (on the left) for **2i** (green) in p53 binding site of MDM2 protein in comparison to nutlin-3a (grey), as well as its ligand-protein interaction map (on the right).

Biology

Supplementary Table S1. Cytotoxicity of synthesized 2,4,5-triaryl cis-imidazoline derivatives to HCT-116 cell line.

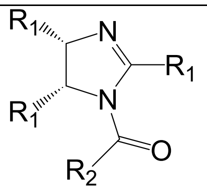

ID	R1	R2	IC50, µm	ID	R1	R2	IC50, µM
**2a**	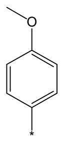	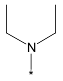	54	**2o**	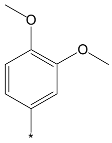	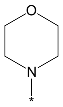	131
**2b**	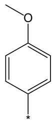	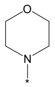	>200	**2p**	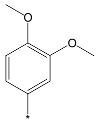	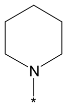	102
**2c**	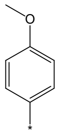	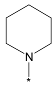	54	**2q**	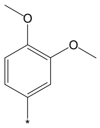	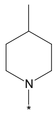	112
**2d**	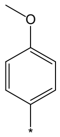	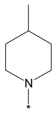	38	**2r**	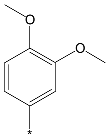	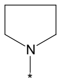	>200

**2e**	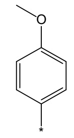	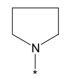	115	**2s**	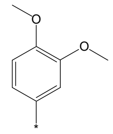	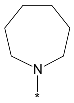	77
**2f**	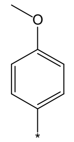	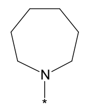	27	**2t**	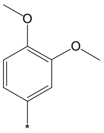	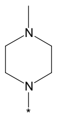	>200
**2g**	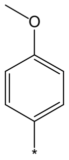	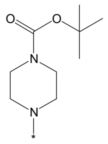	41	**2u**	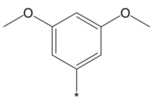	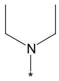	9
**2h**	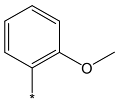	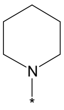	59	**2v**	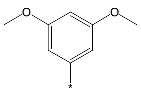	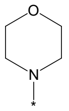	98
**2i**	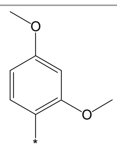	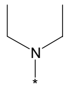	30	**2w**	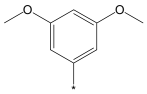	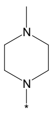	65
**2j**	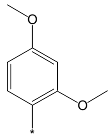	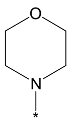	126	**2x**	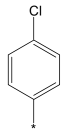	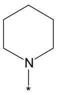	11
**2k**	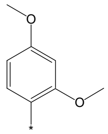	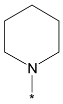	23	**2y**	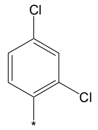	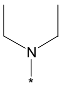	24
**2l**	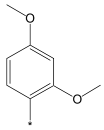	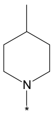	16	**2z**	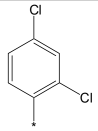	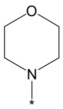	17
**2m**	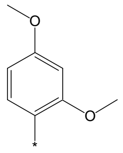	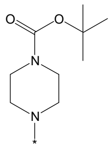	13	**2aa**	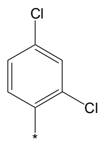	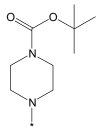	21
**2n**	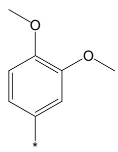	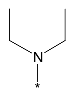	51				

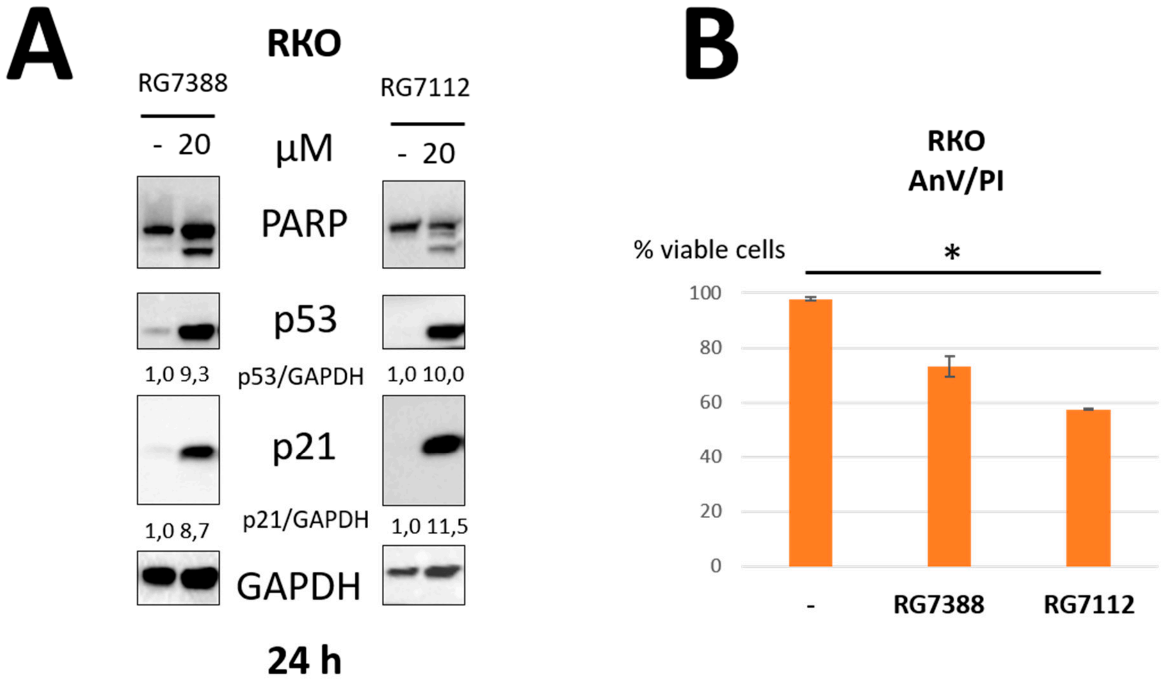

Supplementary Figure S4 A. Western Blot analysis of total cellular lysates from RKO cells upon treatment with RG7388 and RG7112. B. The histograms of flow cytometry (FC) analysis data for RKO cells using Annexin V-FITC / PI staining, % viable cells – cells negative for both Annexin V-FITC and propidium iodide (PI). Data from n = 3 biological replicates were shown as mean ± s.d., * $p < 0.05$, n.s. – not significant. PARP – poly (ADP-ribose)-polymerase; GAPDH – glyceraldehyde 3-phosphate dehydrogenase; p53/GAPDH and p21/GAPDH – densitometric analysis of p53 or p21 bands normalized to GAPDH, h – hours.

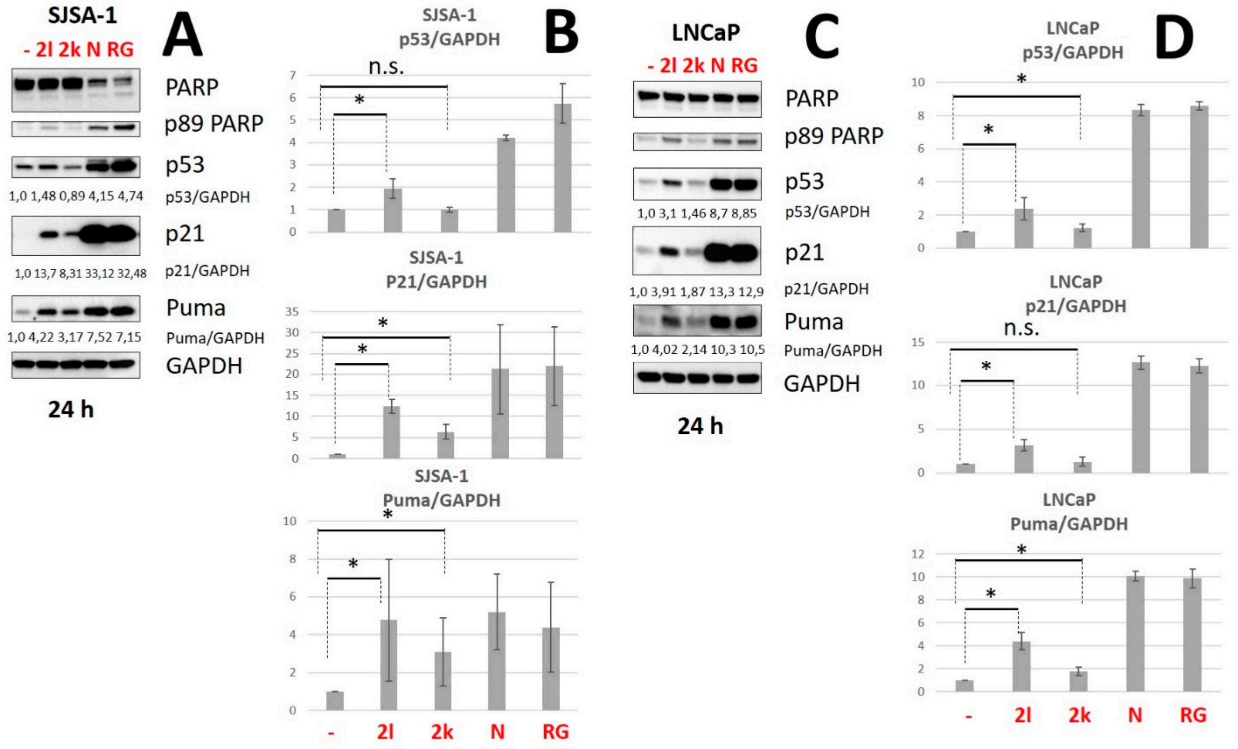

Supplementary Figure S5 A, C. Western Blot analysis of total cellular lysates from SJSA-1 (A) and LNCaP (C) cells upon treatment with compounds **2l, 2k**, Nutlin-3a and RG7388. B, D - Densitometric analysis of p53, p21 and Puma bands normalized to GAPDH in SJSA-1 (B) and LNCaP (D) cells. Data from n = 3 biological replicates were shown as mean ± s.d., * $p < 0.05$, n.s. – not significant. PARP – poly (ADP-ribose)-polymerase; GAPDH – glyceraldehyde 3-phosphate dehydrogenase; p53/GAPDH, p21/GAPDH and Puma/GAPDH – densitometric analysis of p53, p21 and Puma bands normalized to GAPDH, h – hours, N - Nutlin-3a (10 μM), R – RG7388 (5 μM).

Chemistry (1H and 13C NMR data for compounds 2a-2aa)

***1H NMR Cis-N,N*-diethyl-2,4,5-*tris*(4-methoxyphenyl)-4,5-dihydro-1H-imidazole-1-carboxamide (2a)**

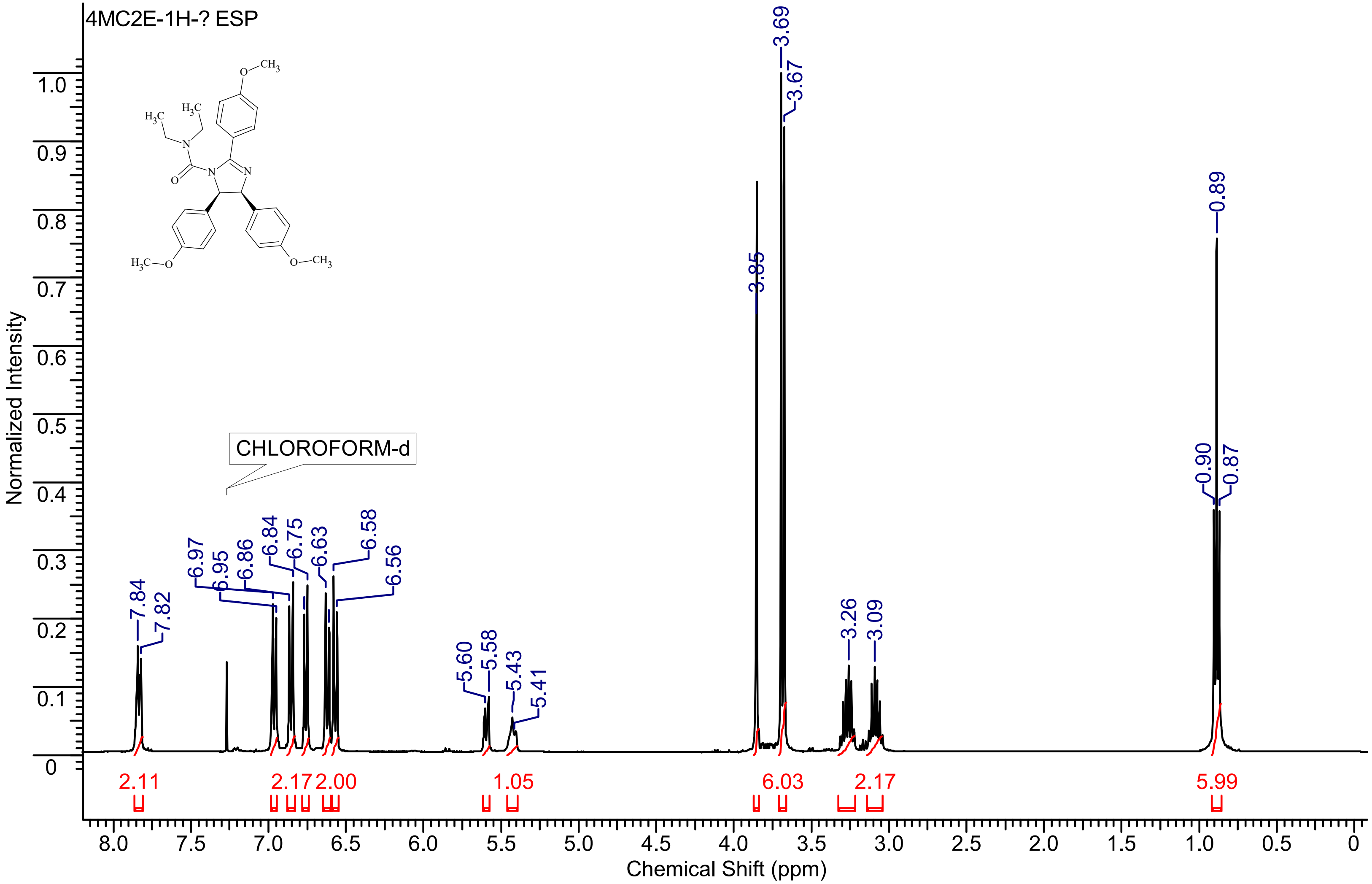

***13C NMR Cis-N,N*-diethyl-2,4,5-*tris*(4-methoxyphenyl)-4,5-dihydro-1H-imidazole-1-carboxamide (2a)**

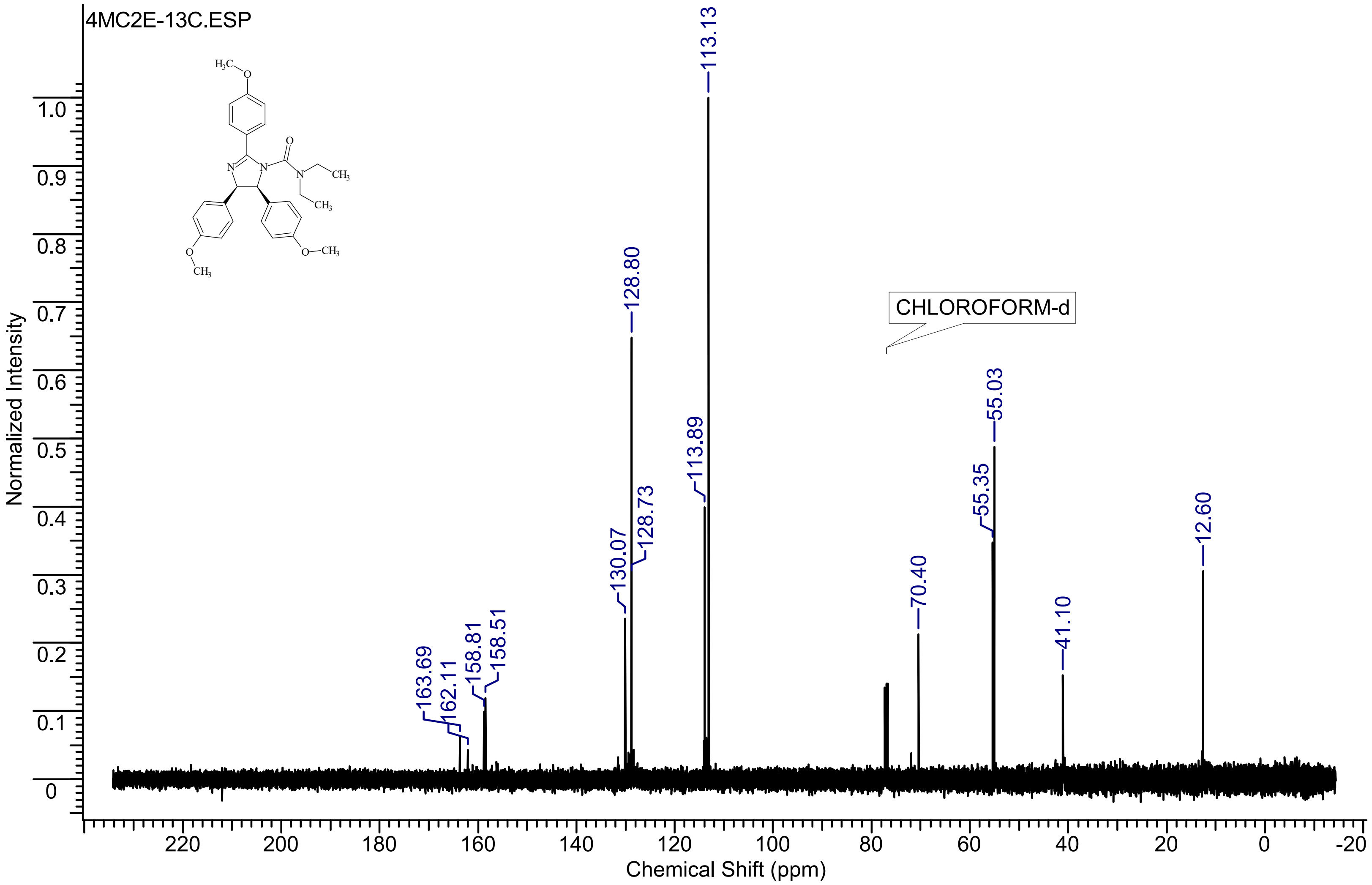

1H NMR 4-{[*Cis*-2,4,5-*tris*(4-methoxyphenyl)-4,5-dihydro-1H-imidazol-1-yl]carbonyl}morpholine (2b).

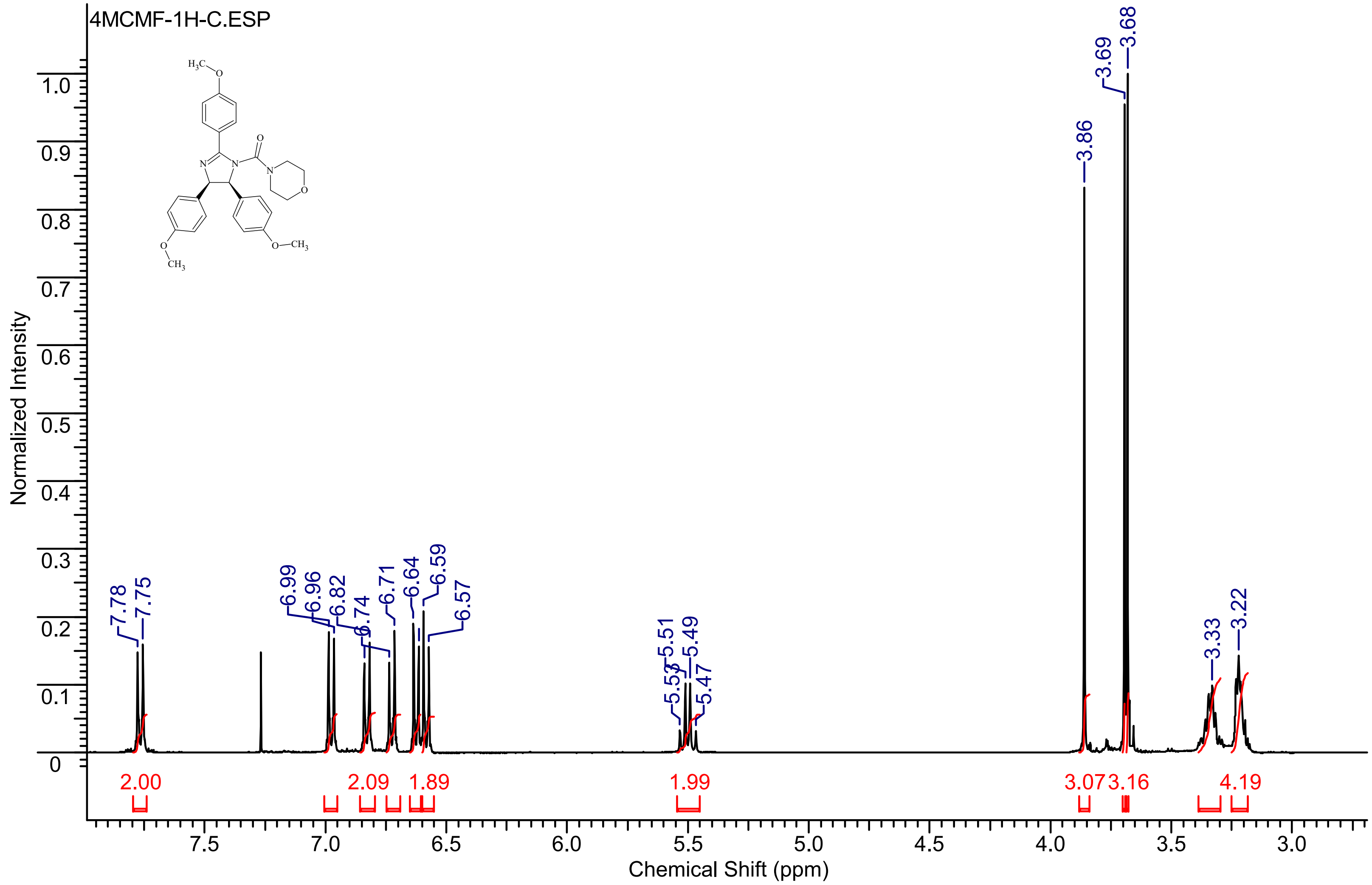

13C NMR 4-{[*Cis*-2,4,5-*tris*(4-methoxyphenyl)-4,5-dihydro-1H-imidazol-1-yl]carbonyl}morpholine (2b).

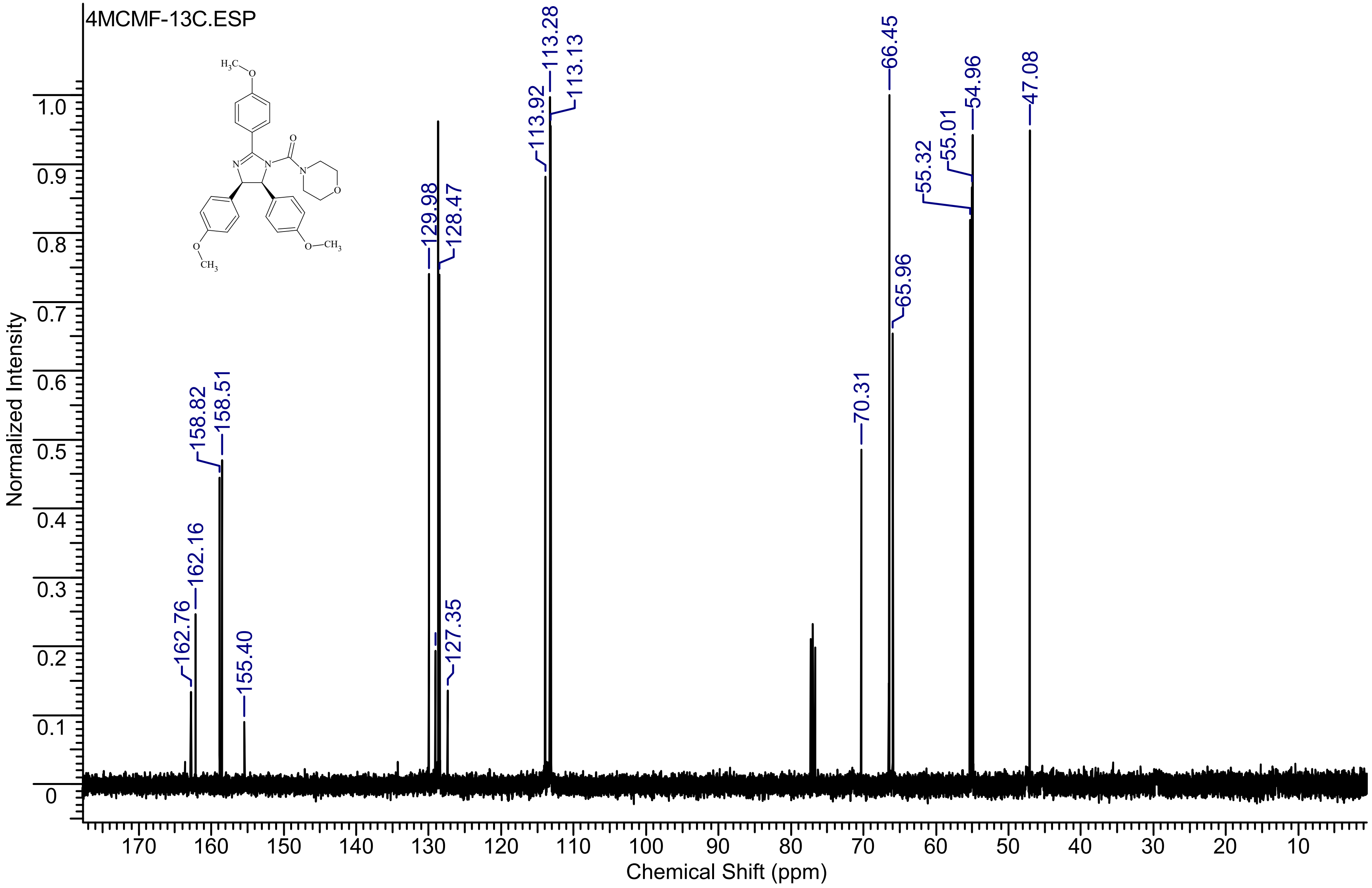

1H NMR Piperidin-1-yl(*cis*-2,4,5-*tris*(4-methoxyphenyl)-4,5-dihydro-*1H*-imidazol-1-yl)methanone (2c).

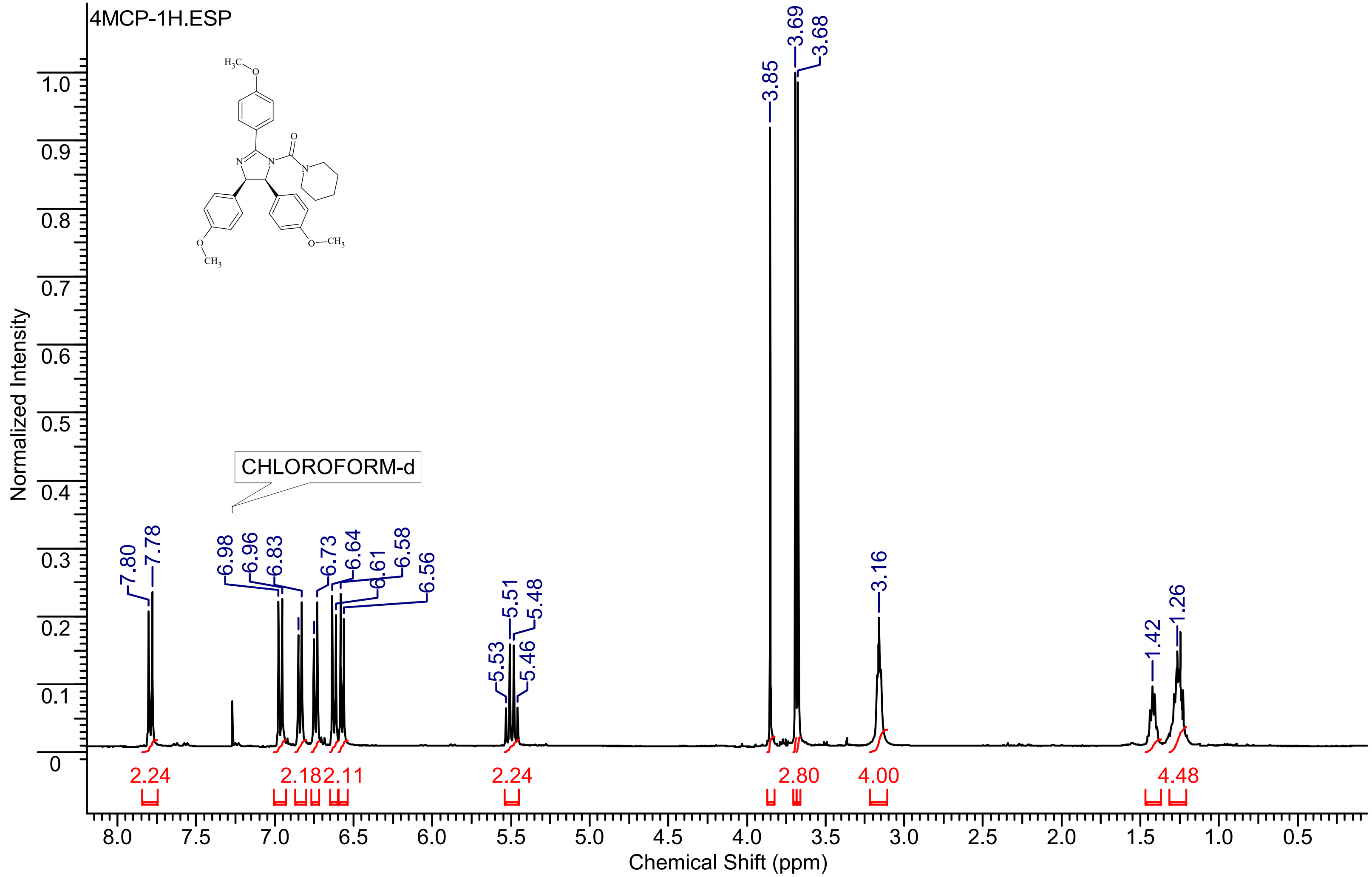

13C NMR Piperidin-1-yl(*cis*-2,4,5-*tris*(4-methoxyphenyl)-4,5-dihydro-*1H*-imidazol-1-yl)methanone (2c).

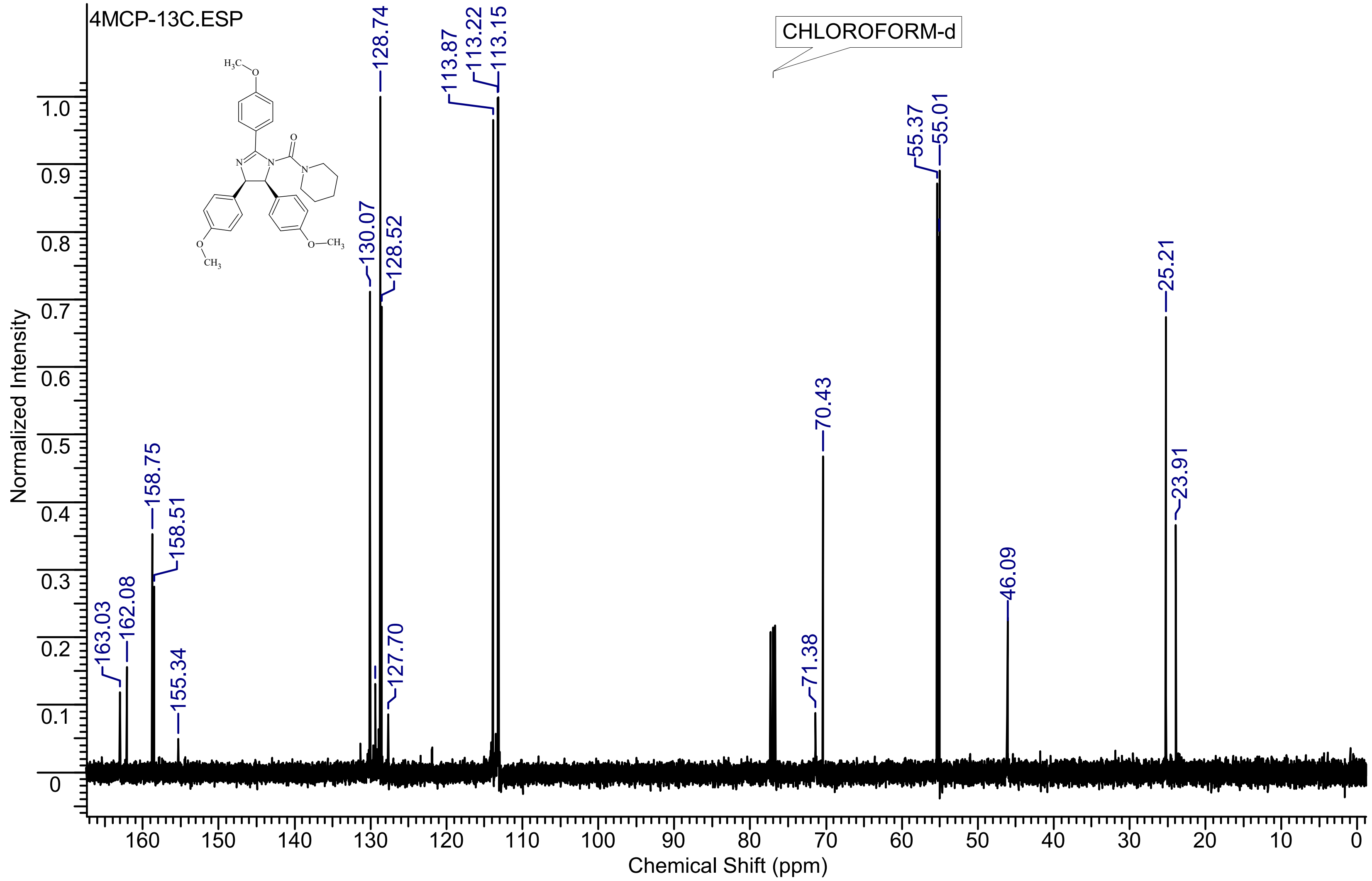

1H NMR (4-Methylpiperidin-1-yl) (*cis*-2,4,5-*tris*(4-methoxyphenyl)-4,5-dihydro-*1H*-imidazol-1-yl)methanone (2d).

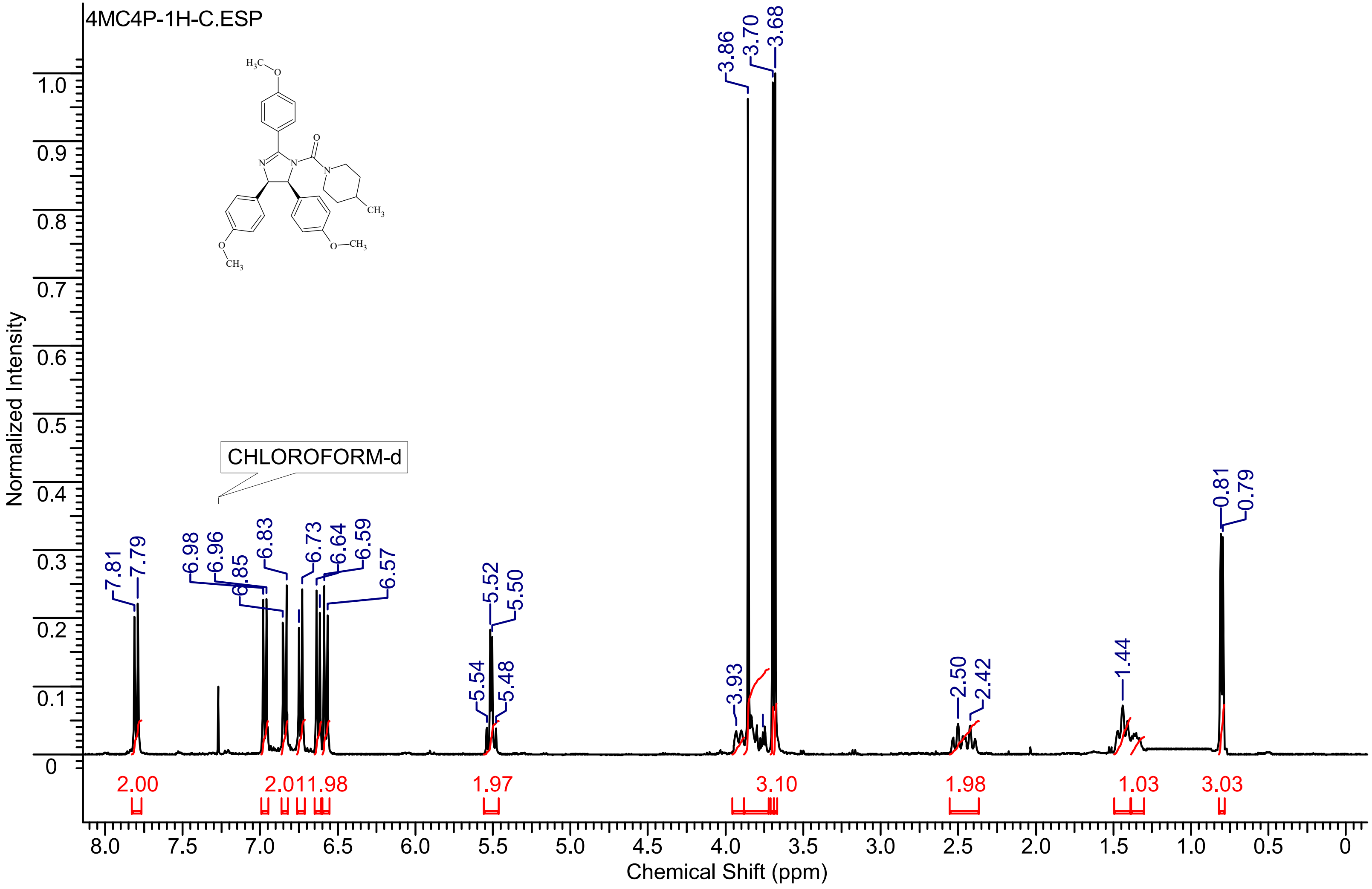

13C NMR (4-Methylpiperidin-1-yl) (*cis*-2,4,5-*tris*(4-methoxyphenyl)-4,5-dihydro-*1H*-imidazol-1-yl)methanone (2d).

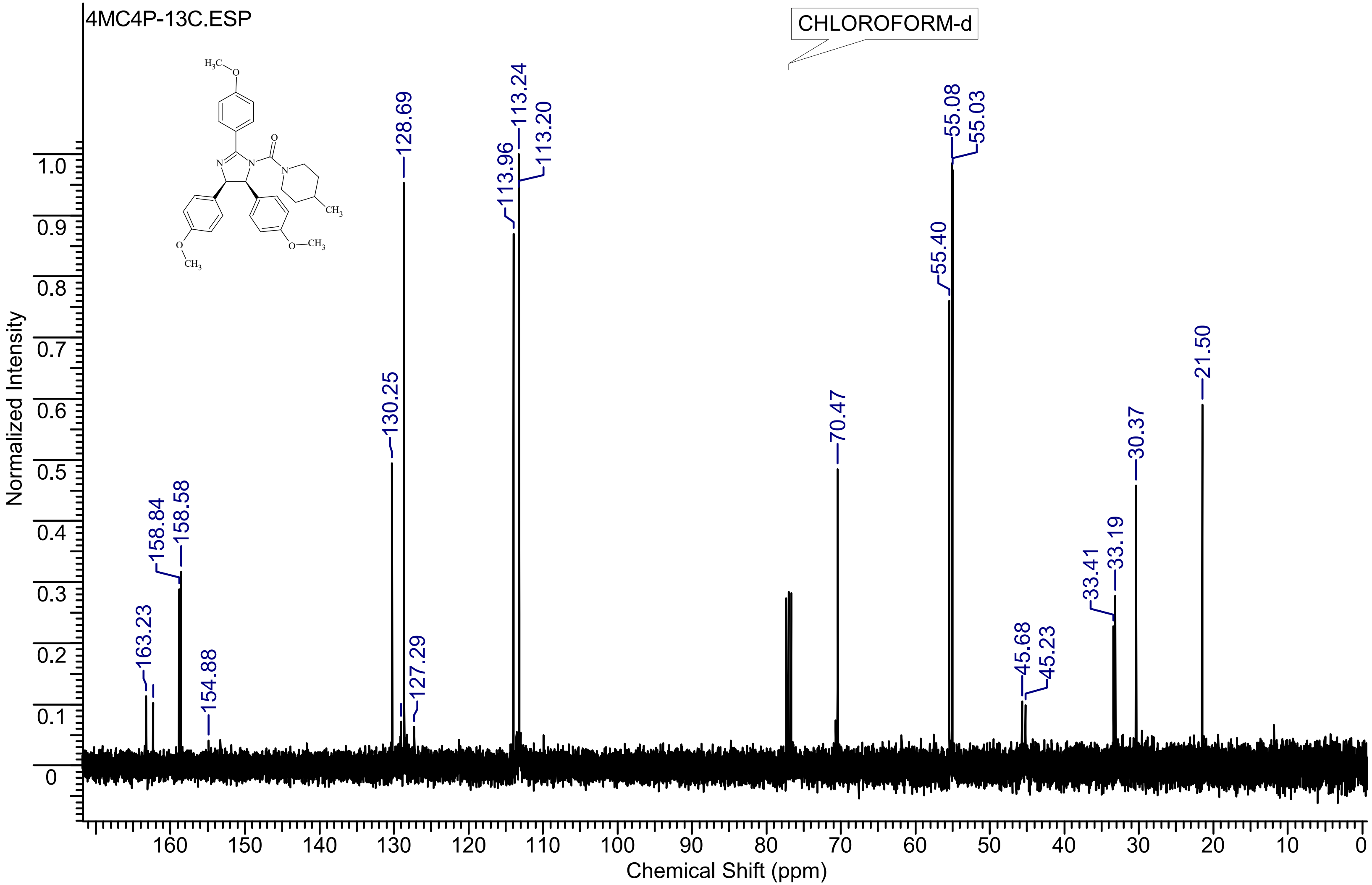

1H NMR Pyrrolidin-1-yl(*cis*-2,4,5-*tris*(4-methoxyphenyl)-4,5-dihydro-*1H*-imidazol-1-yl)methanone (2e).

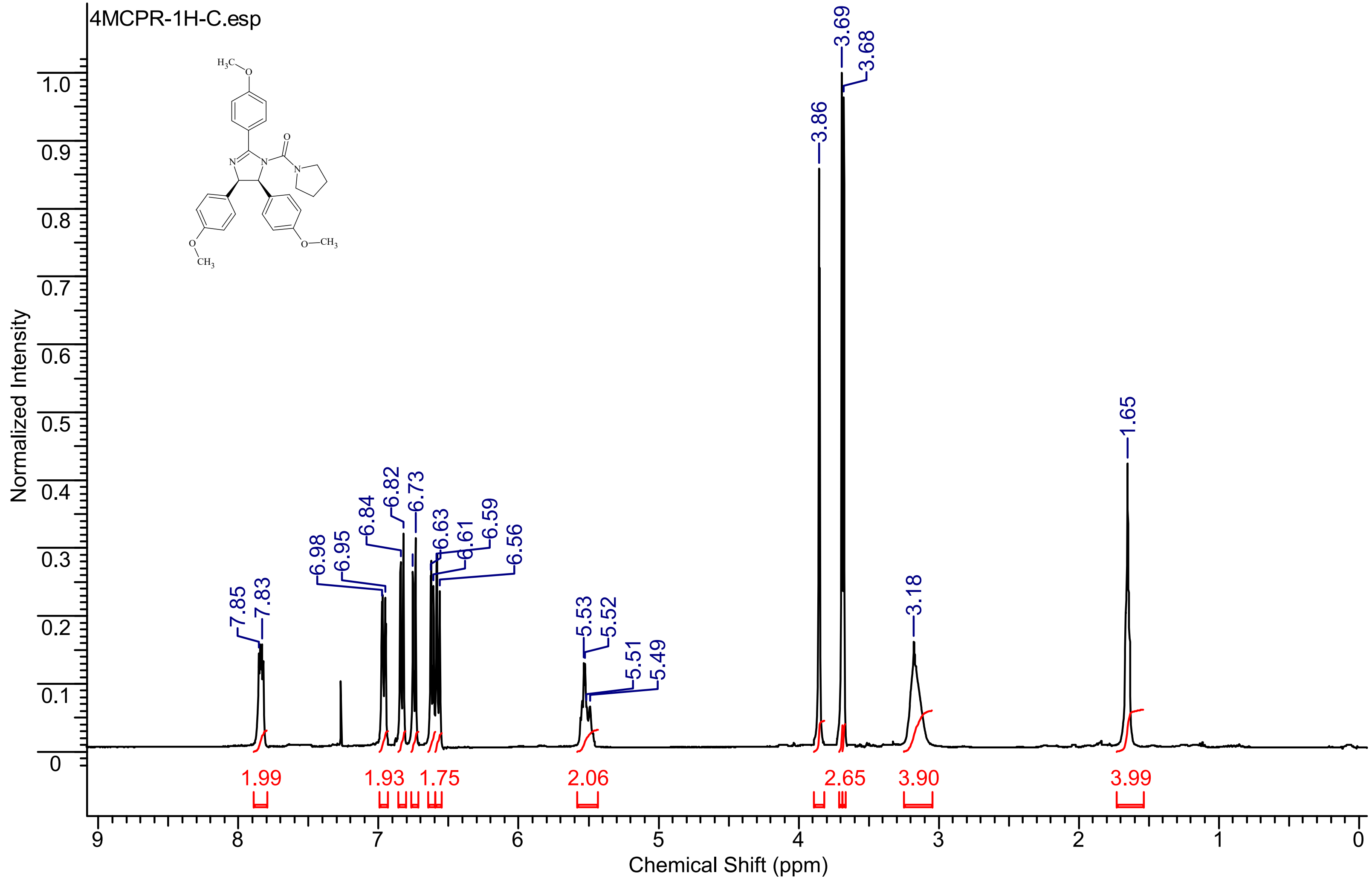

13C NMR Pyrrolidin-1-yl(*cis*-2,4,5-*tris*(4-methoxyphenyl)-4,5-dihydro-*1H*-imidazol-1-yl)methanone (2e).

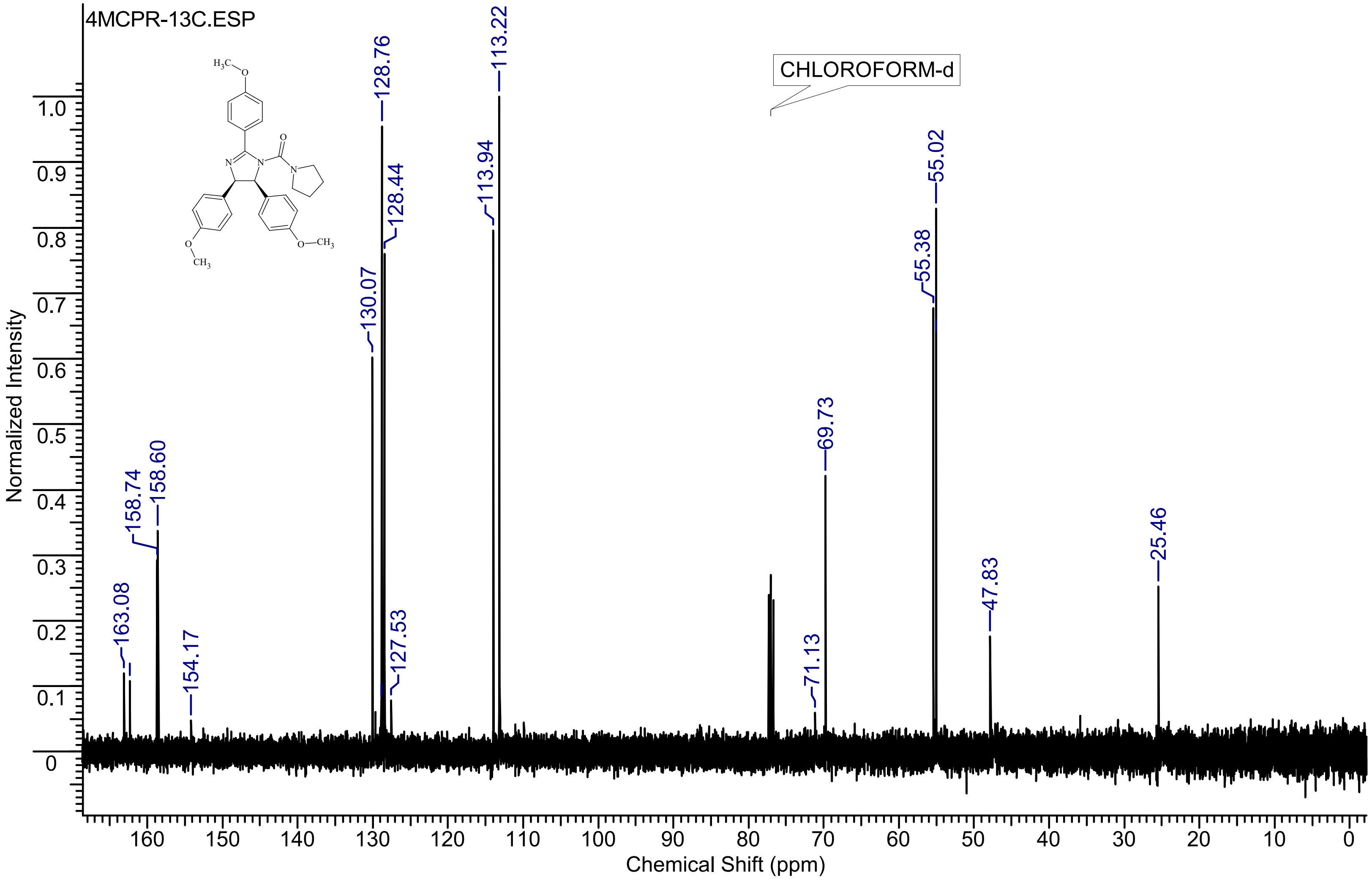

1H NMR Azepan-1-yl(*cis*-2,4,5-*tris*(4-methoxyphenyl)-4,5-dihydro-*1H*-imidazol-1-yl)methanone (2f).

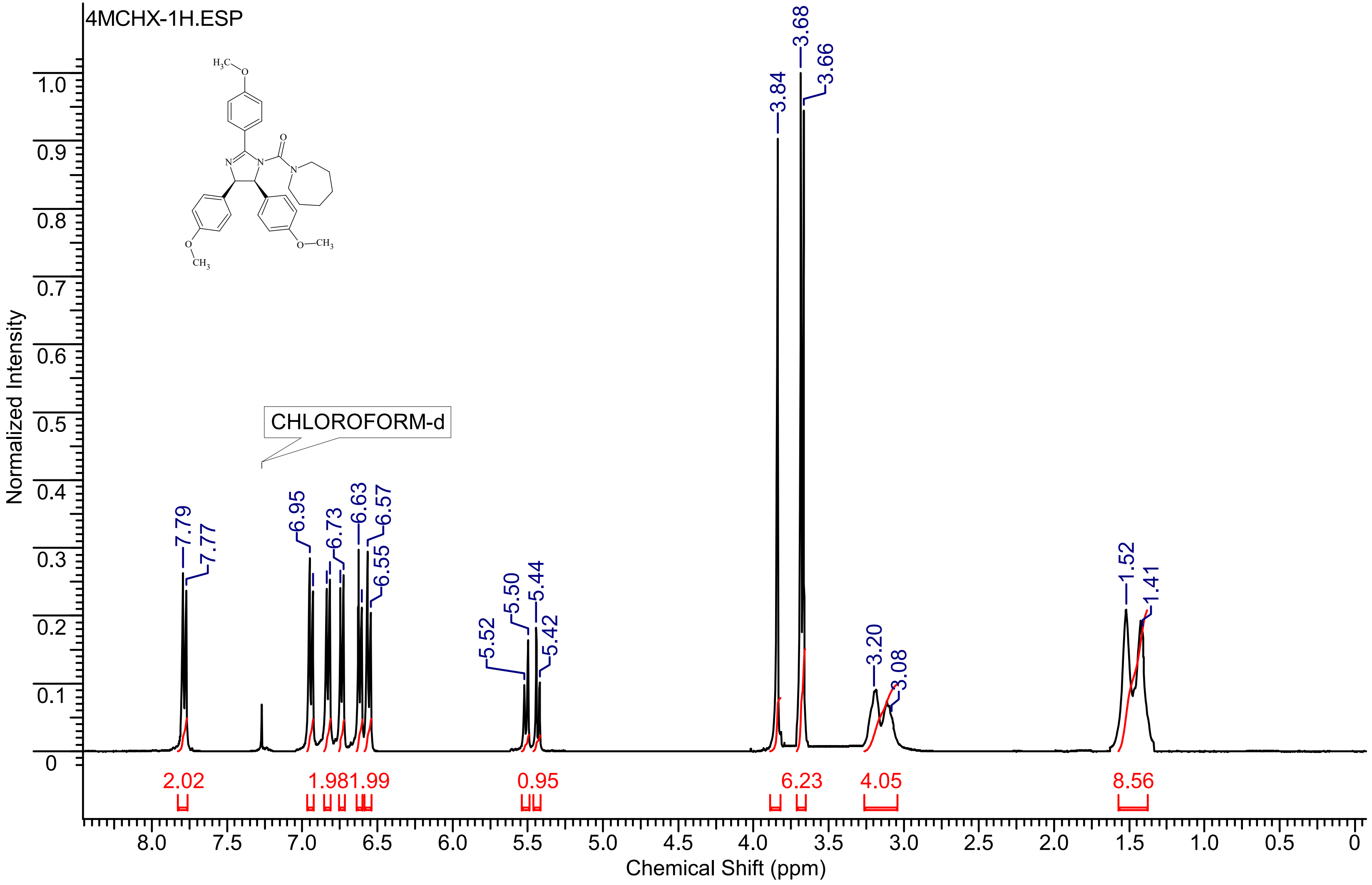

13C NMR Azepan-1-yl(*cis*-2,4,5-*tris*(4-methoxyphenyl)-4,5-dihydro-*1H*-imidazol-1-yl)methanone (2f).

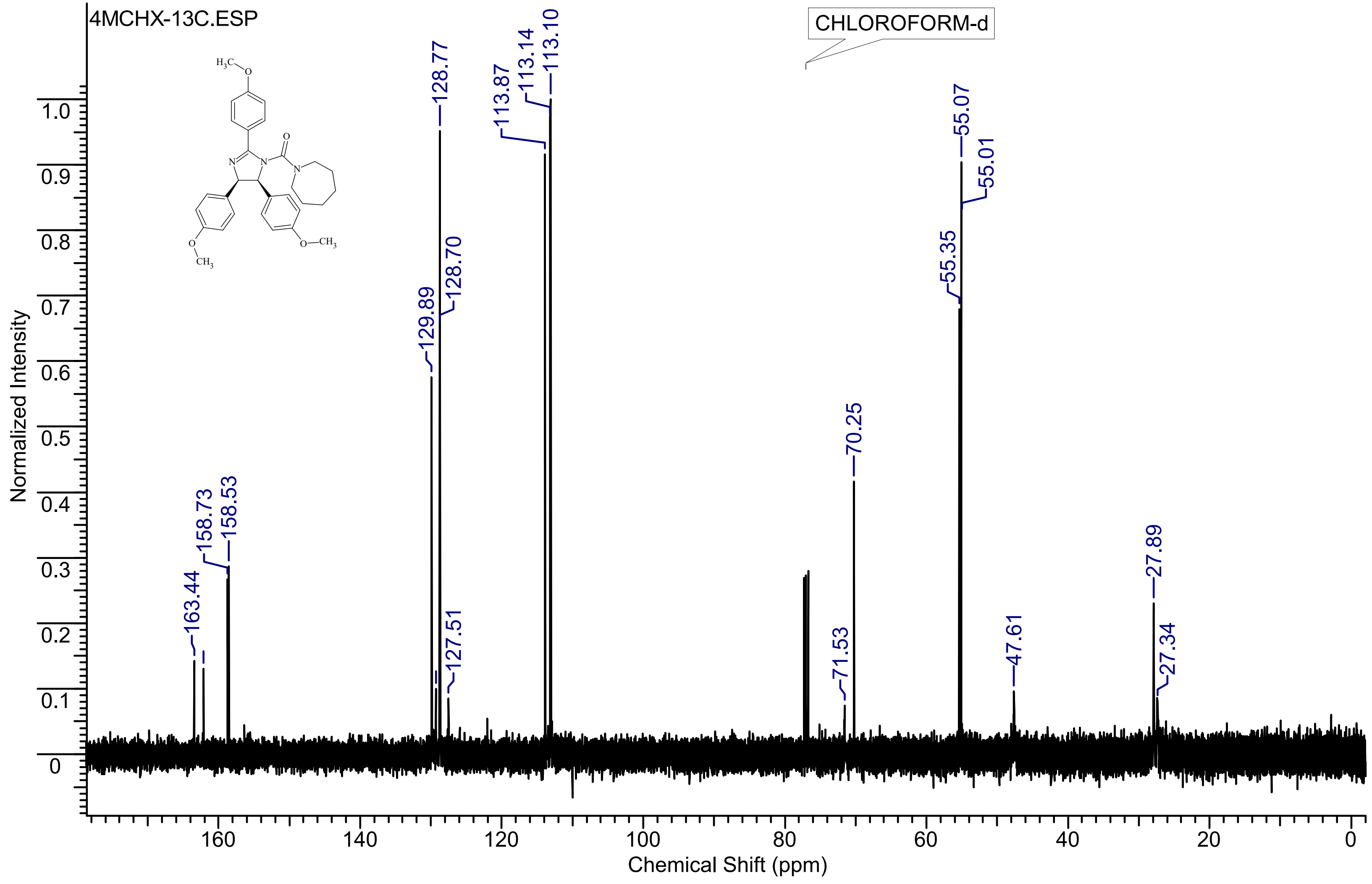

1H NMR *tert*-Butyl 4-(*cis*-2,4,5-*tris*(4-methoxyphenyl)-4,5-dihydro-*1H*-imidazole-1-carbonyl)piperazine-1-carboxylate (2g).

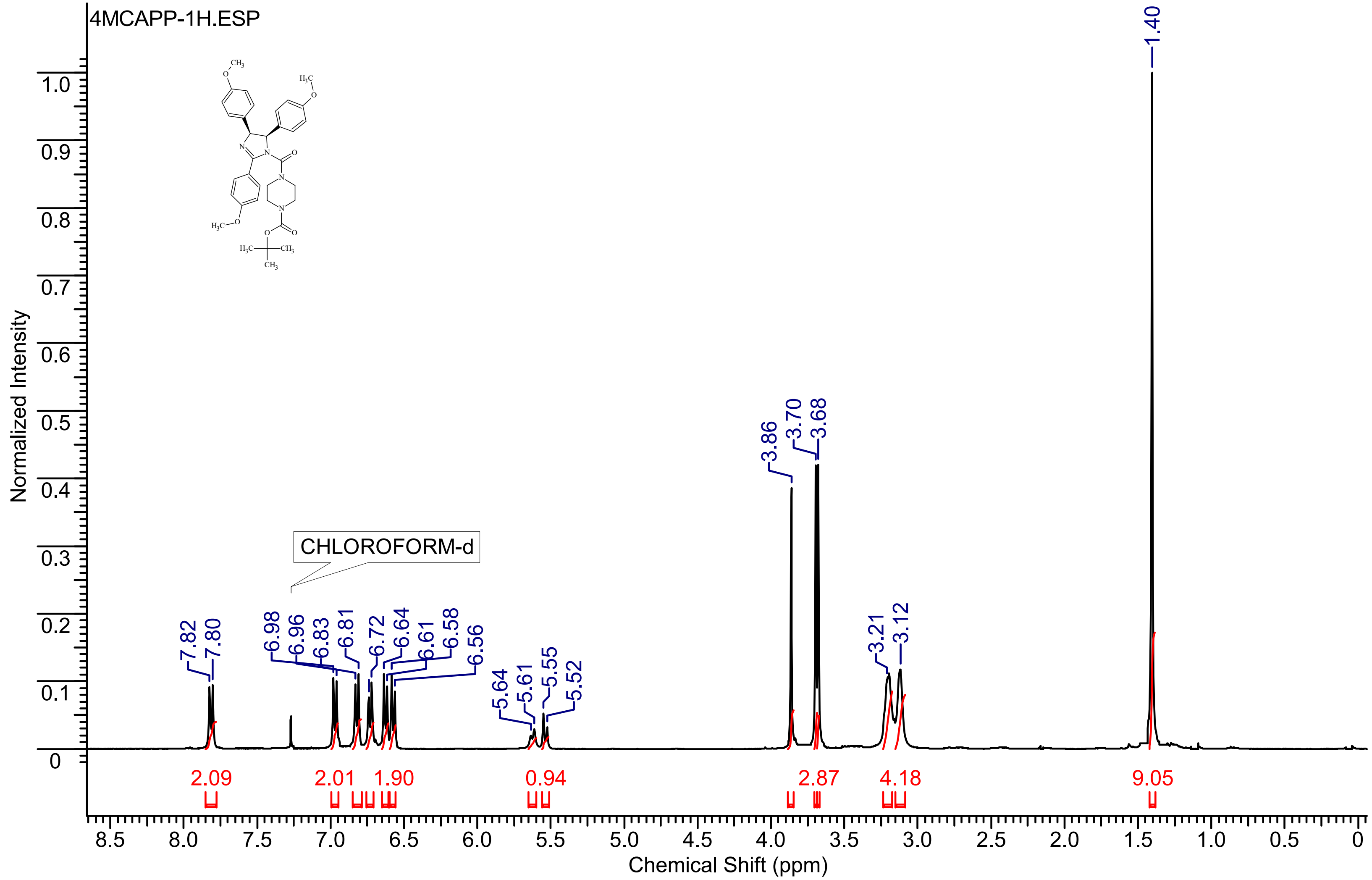

13C NMR *tert*-Butyl 4-(*cis*-2,4,5-*tris*(4-methoxyphenyl)-4,5-dihydro-*1H*-imidazole-1-carbonyl)piperazine-1-carboxylate (2g).

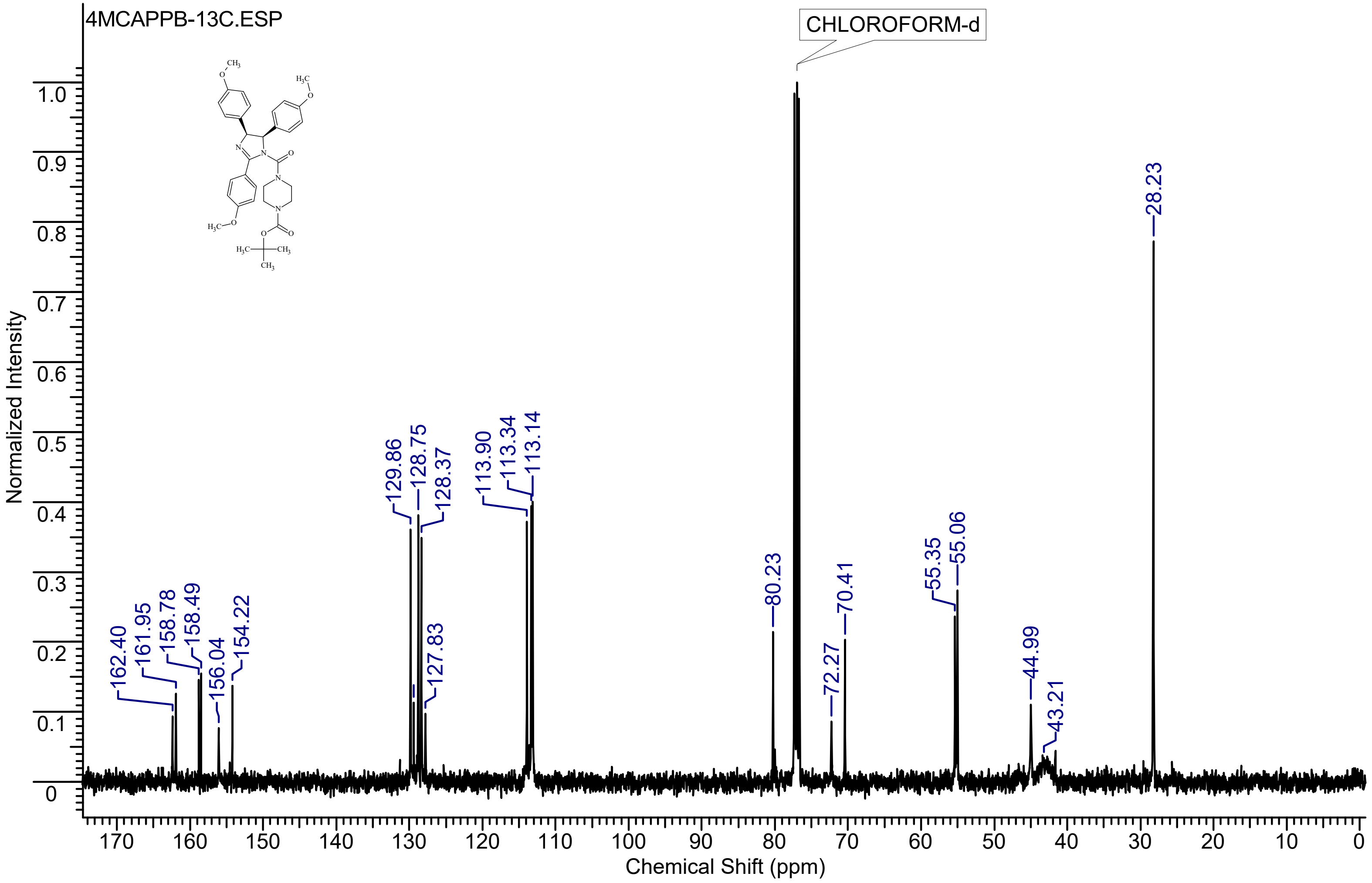

1H NMR Piperidin-1-yl(*cis*-2,4,5-*tris*(2-methoxyphenyl)-4,5-dihydro-*1H*-imidazol-1-yl)methanone (2h).

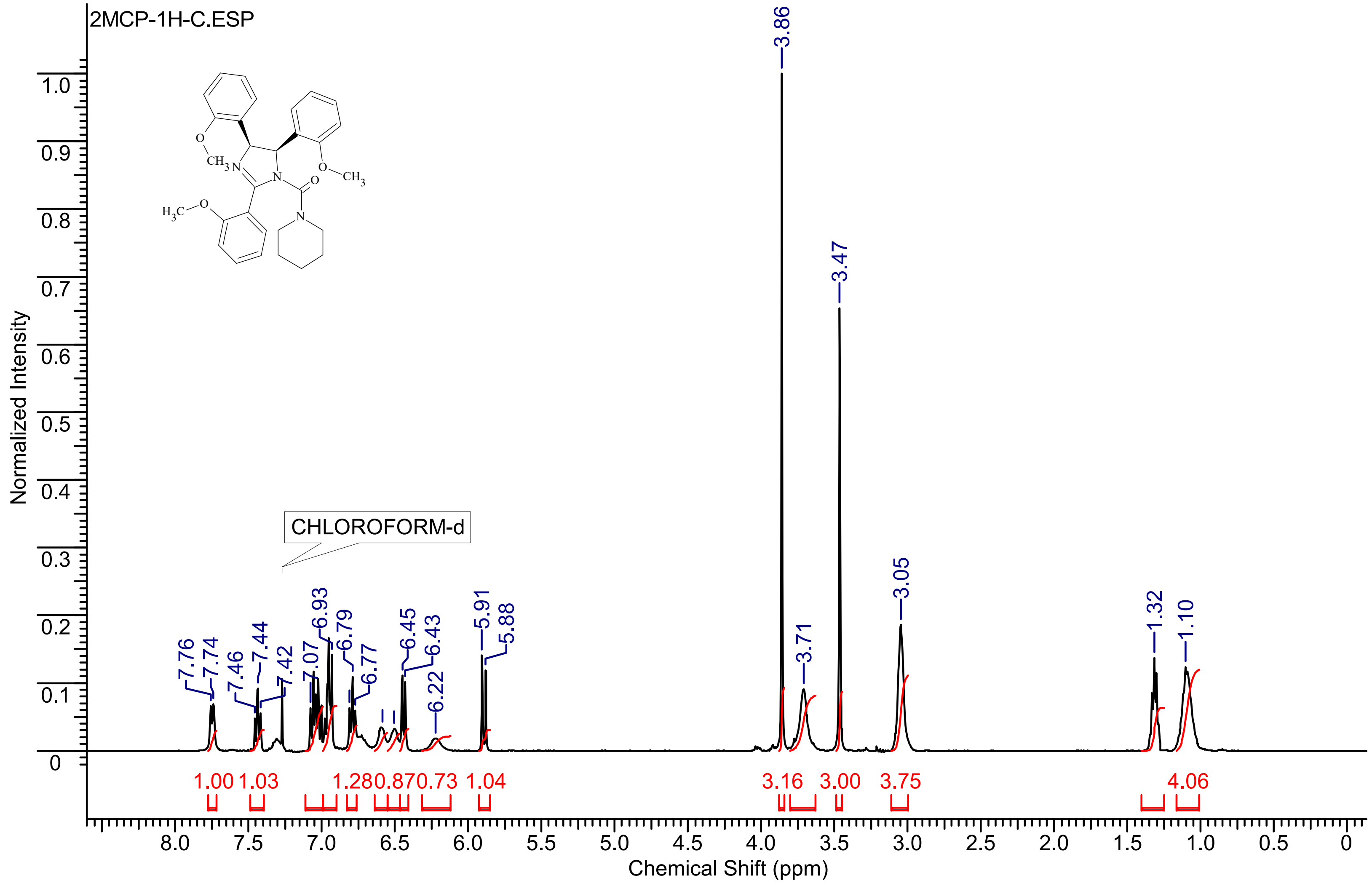

13C NMR Piperidin-1-yl(*cis*-2,4,5-*tris*(2-methoxyphenyl)-4,5-dihydro-*1H*-imidazol-1-yl)methanone (2h).

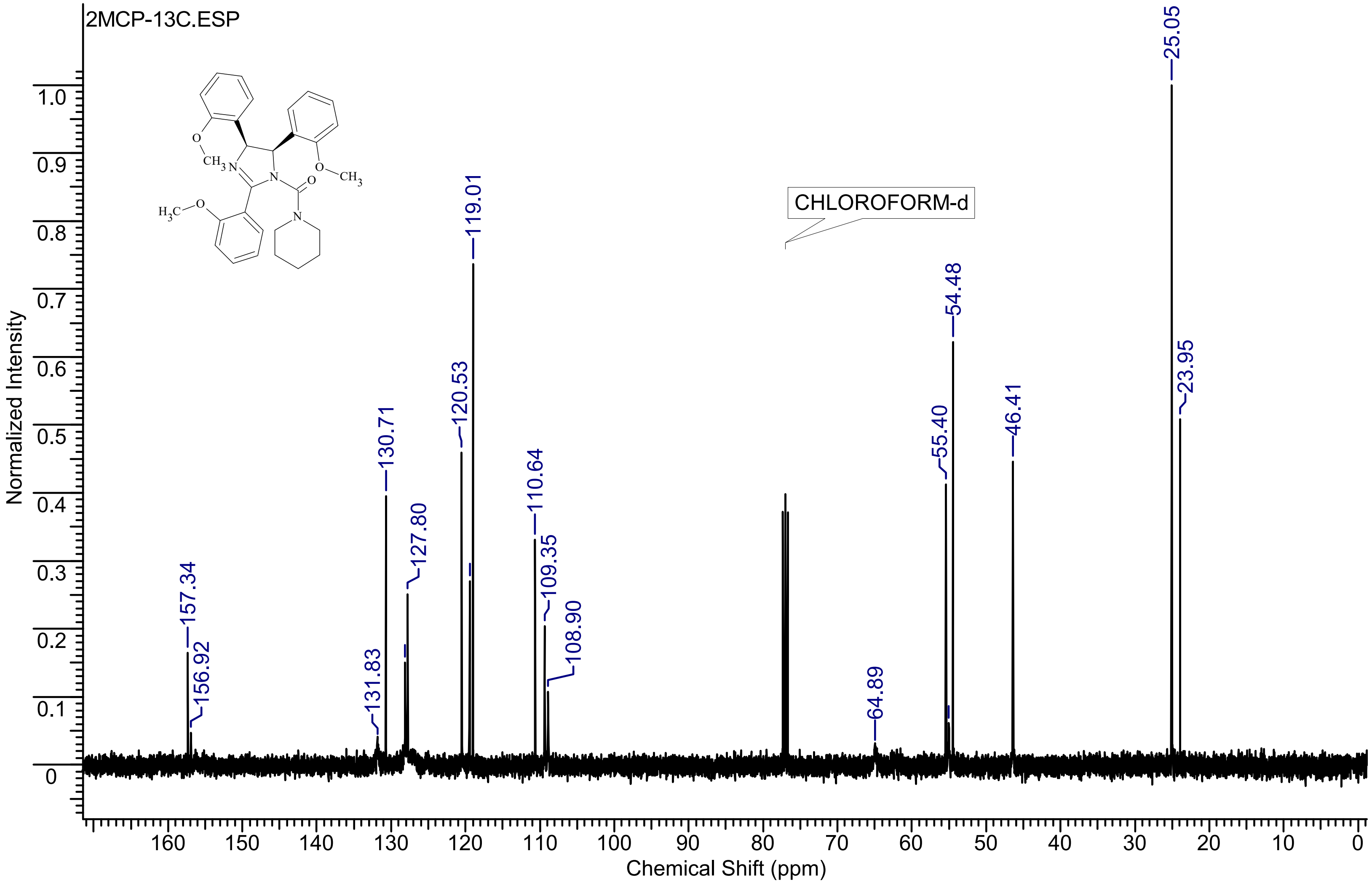

1H NMR *Cis-N,N*-diethyl-2,4,5-*tris*(2,4-dimethoxyphenyl)-4,5-dihydro-*1H*-imidazole-1-carboxamide (2i).

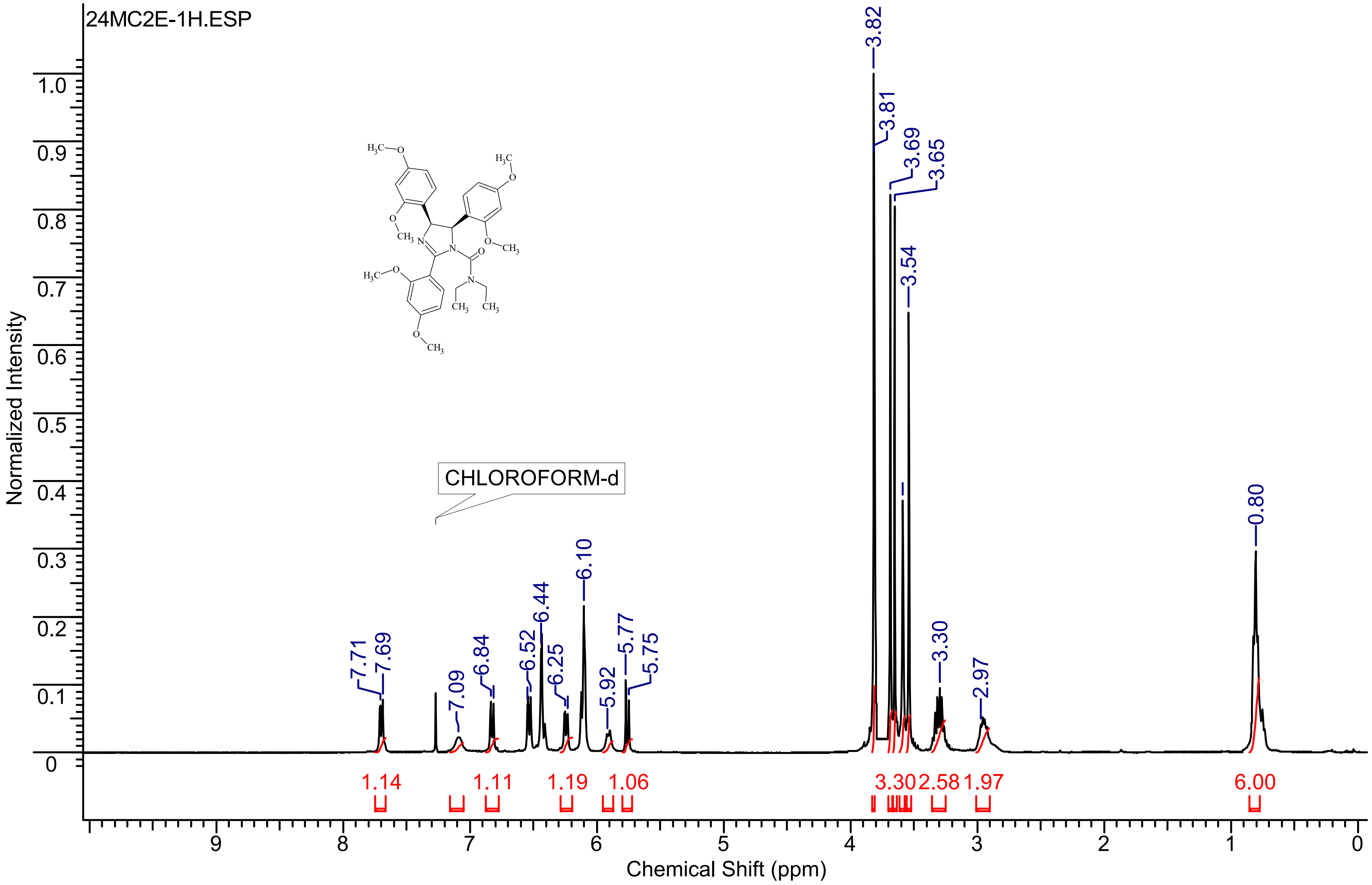

13C NMR *Cis-N,N*-diethyl-2,4,5-*tris*(2,4-dimethoxyphenyl)-4,5-dihydro-*1H*-imidazole-1-carboxamide (2i).

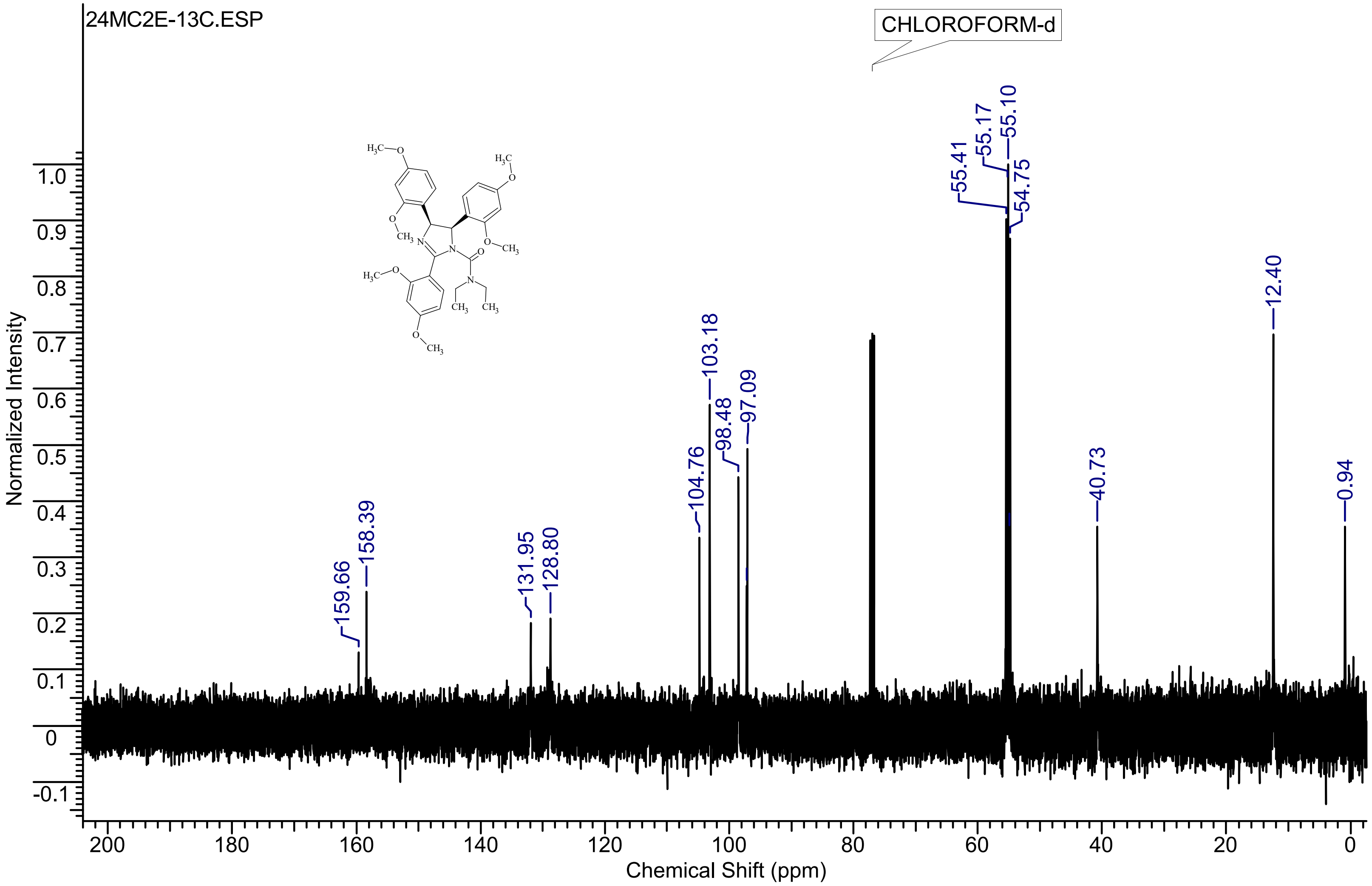

1H NMR 4-{[*Cis*-2,4,5-*tris*(2,4-dimethoxyphenyl)-4,5-dihydro-*1H*-imidazol-1-yl]carbonyl}morpholine (2j).

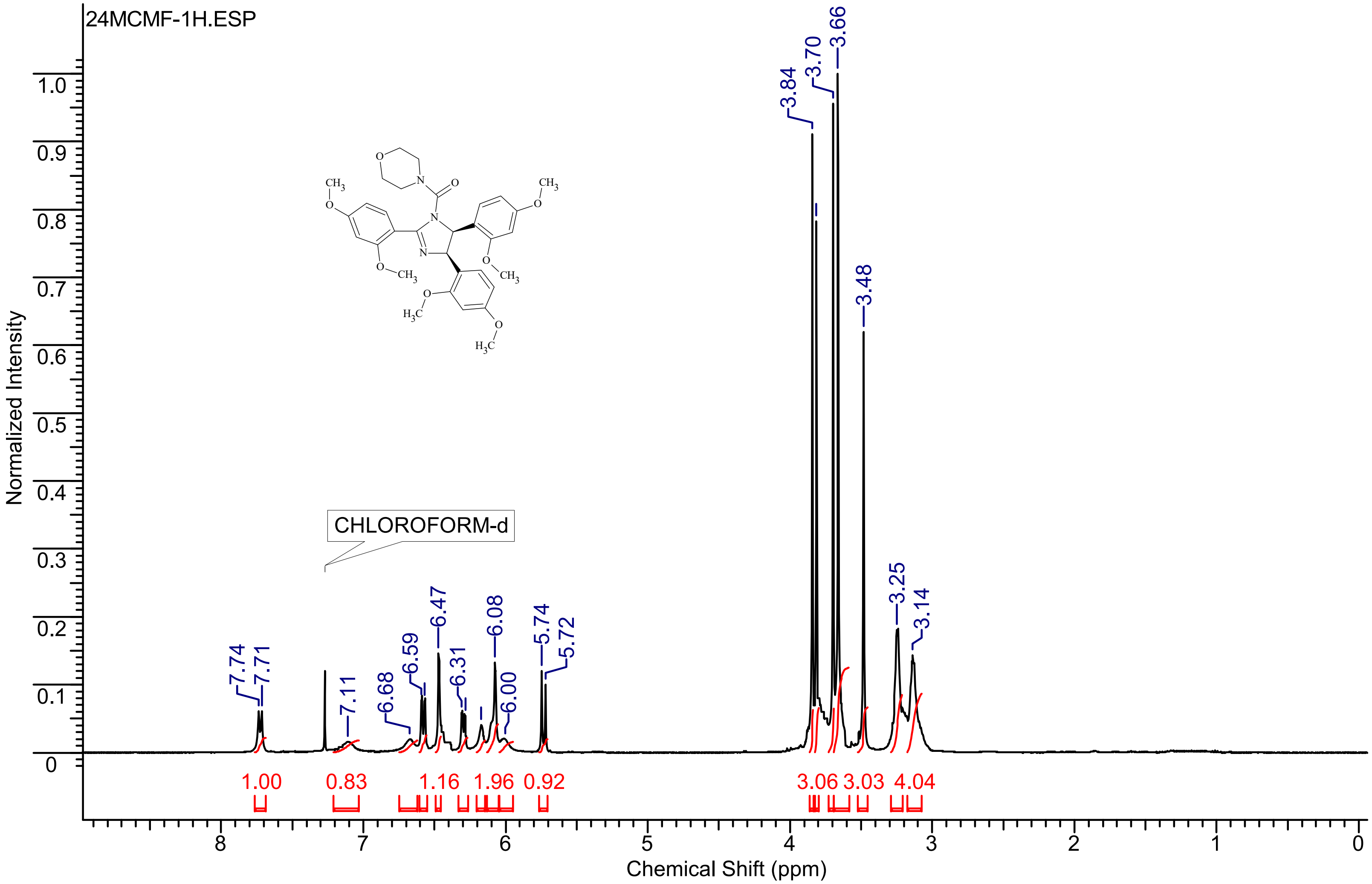

13C NMR 4-{[*Cis*-2,4,5-*tris*(2,4-dimethoxyphenyl)-4,5-dihydro-*1H*-imidazol-1-yl]carbonyl}morpholine (2j).

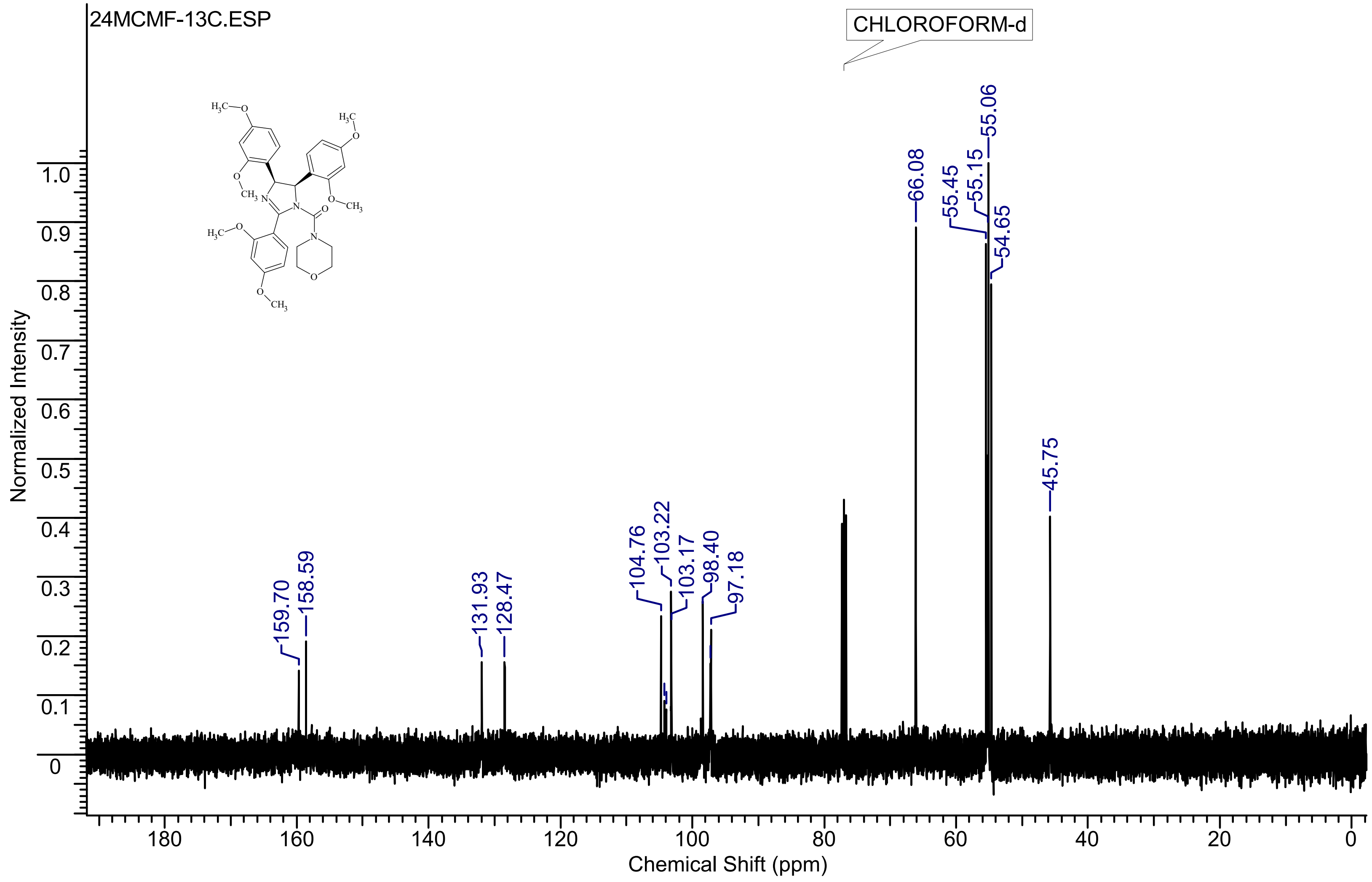

1H NMR Piperidin-1-yl(*cis*-2,4,5-*tris*(2,4-dimethoxyphenyl)-4,5-dihydro-*1H*-imidazol-1-yl)methanone (2k).

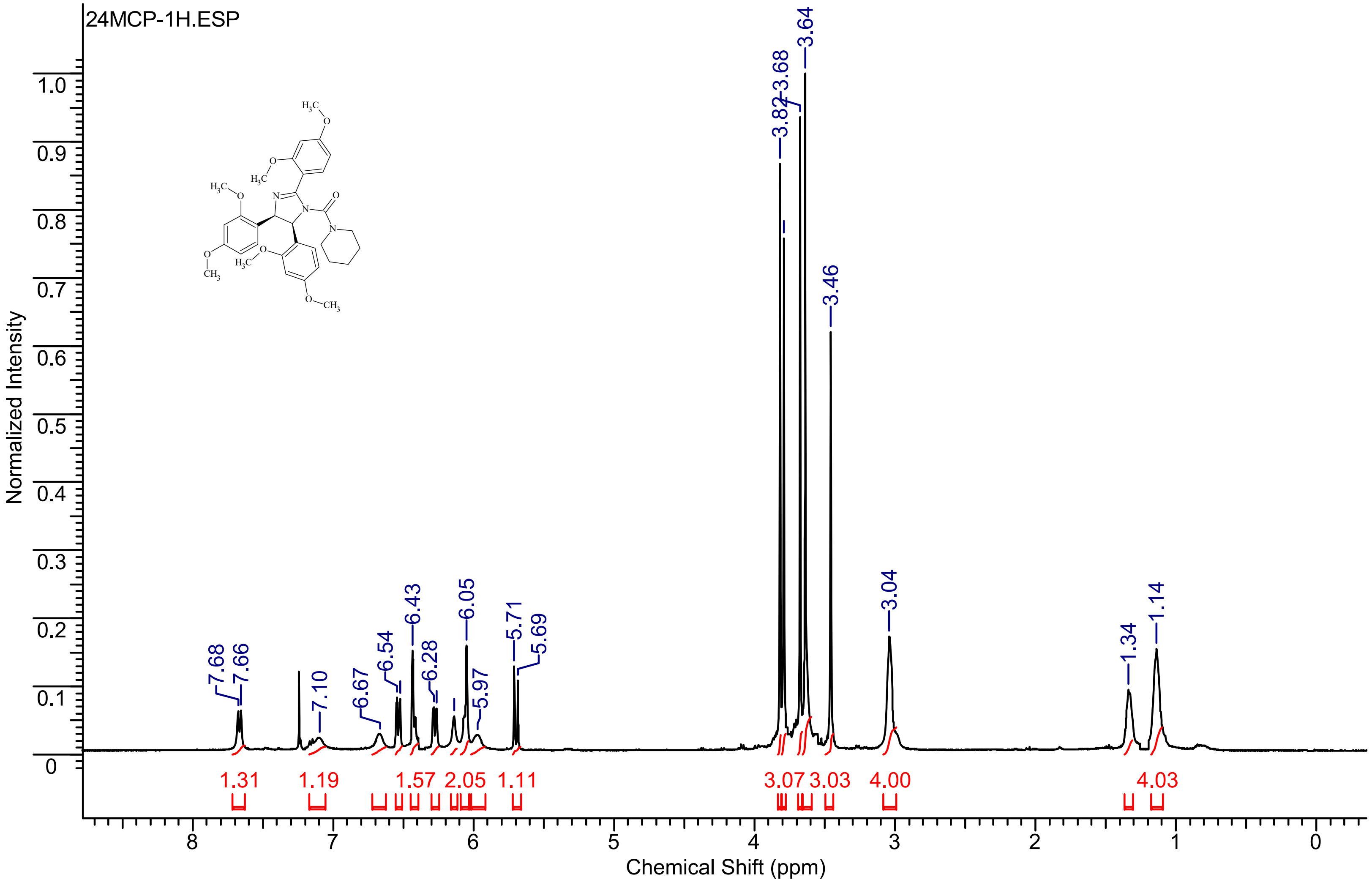

13C NMR Piperidin-1-yl(*cis*-2,4,5-*tris*(2,4-dimethoxyphenyl)-4,5-dihydro-*1H*-imidazol-1-yl)methanone (2k).

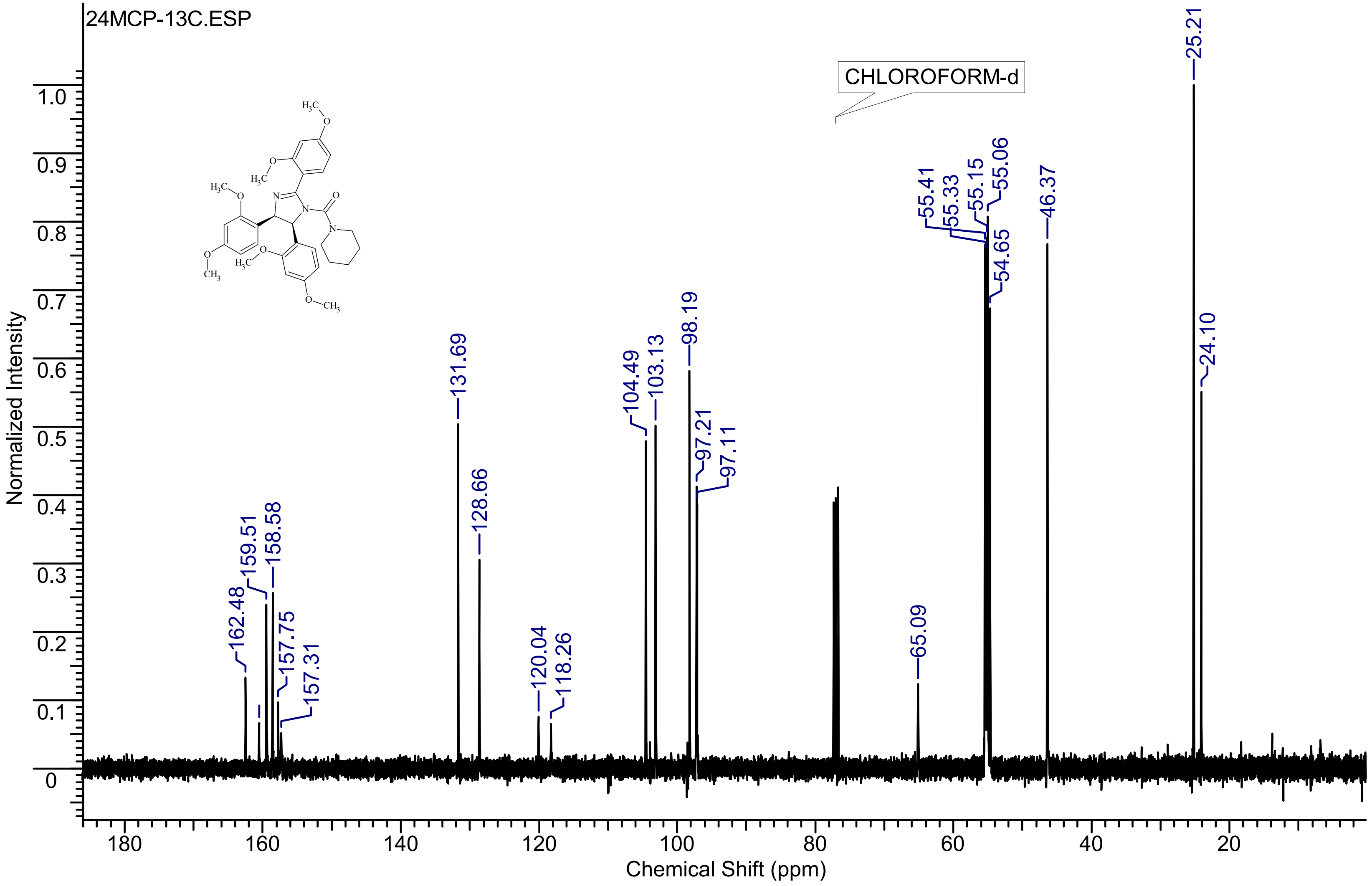

1H NMR (4-Methylpiperidin-1-yl)(*cis*-2,4,5-*tris*(2,4-methoxyphenyl)-4,5-dihydro-*1H*-imidazol-1-yl)methanone (2l).

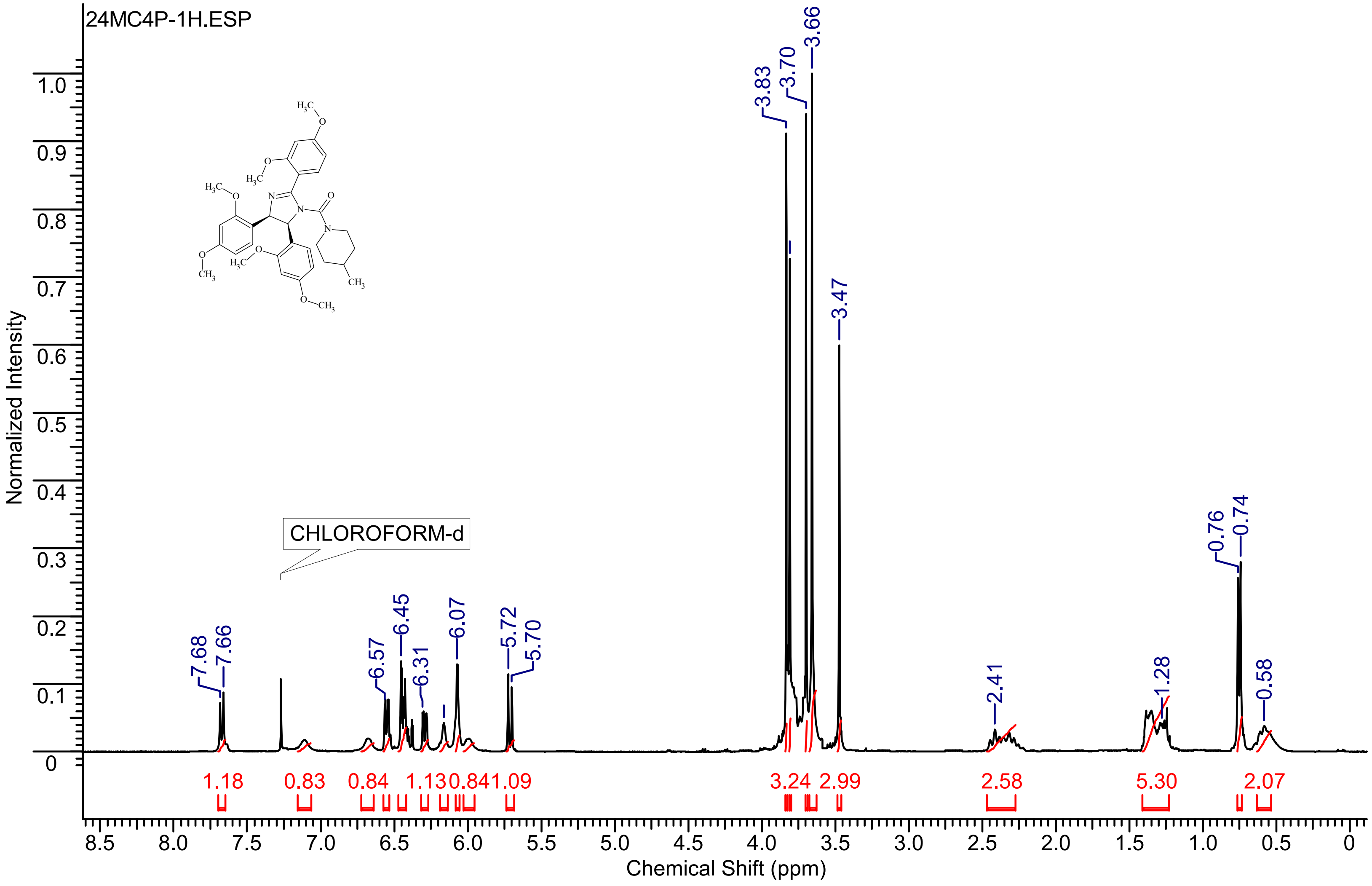

13C NMR (4-Methylpiperidin-1-yl)(*cis*-2,4,5-*tris*(2,4-methoxyphenyl)-4,5-dihydro-*1H*-imidazol-1-yl)methanone (2l).

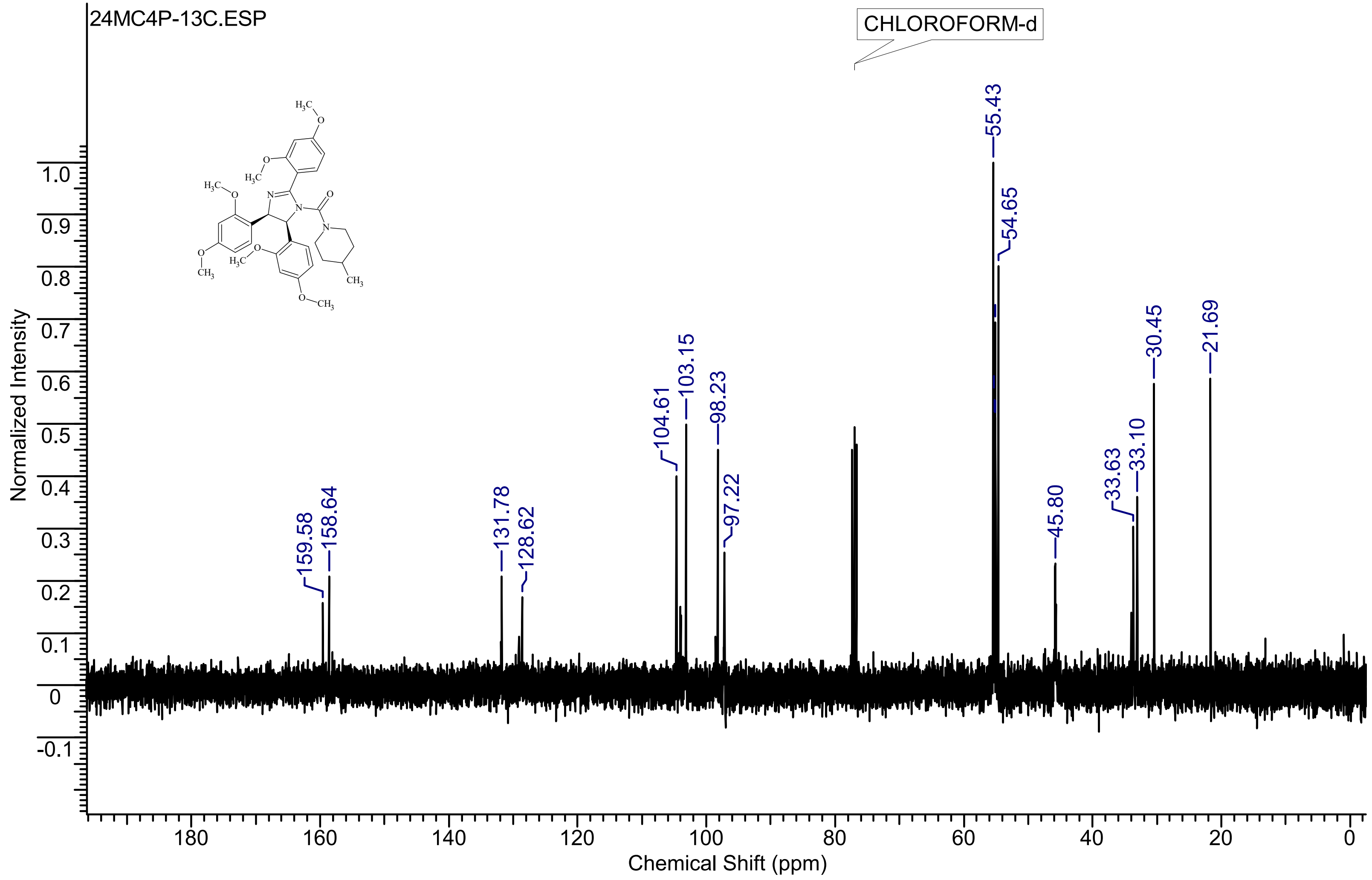

1H NMR *tert*-Butyl 4-(*cis*-2,4,5-*tris*(2,4-dimethoxyphenyl)-4,5-dihydro-*1H*-imidazole-1-carbonyl)piperazine-1-carboxylate (2m).

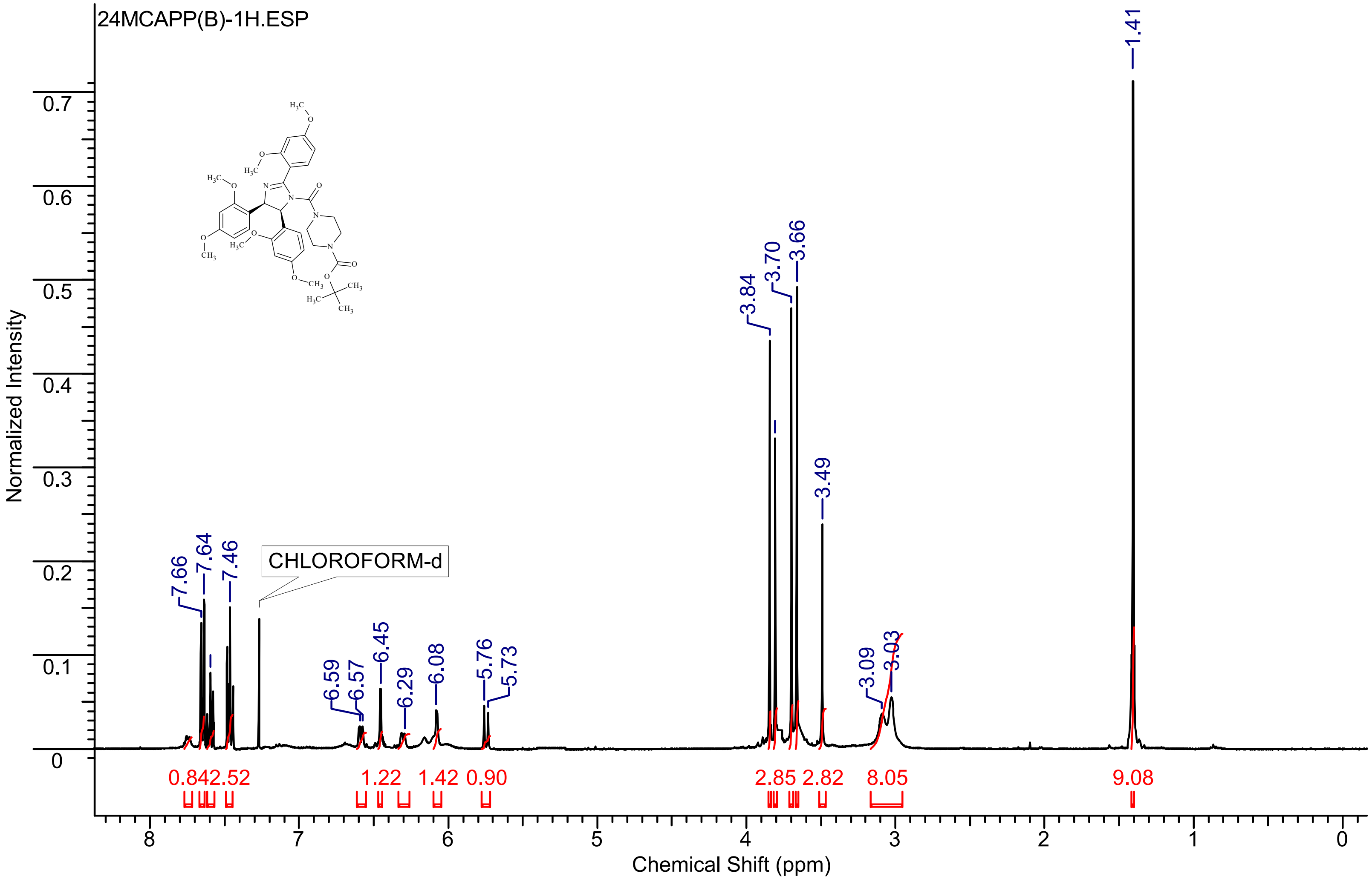

13C NMR *tert*-Butyl 4-(*cis*-2,4,5-*tris*(2,4-dimethoxyphenyl)-4,5-dihydro-*1H*-imidazole-1-carbonyl)piperazine-1-carboxylate (2m).

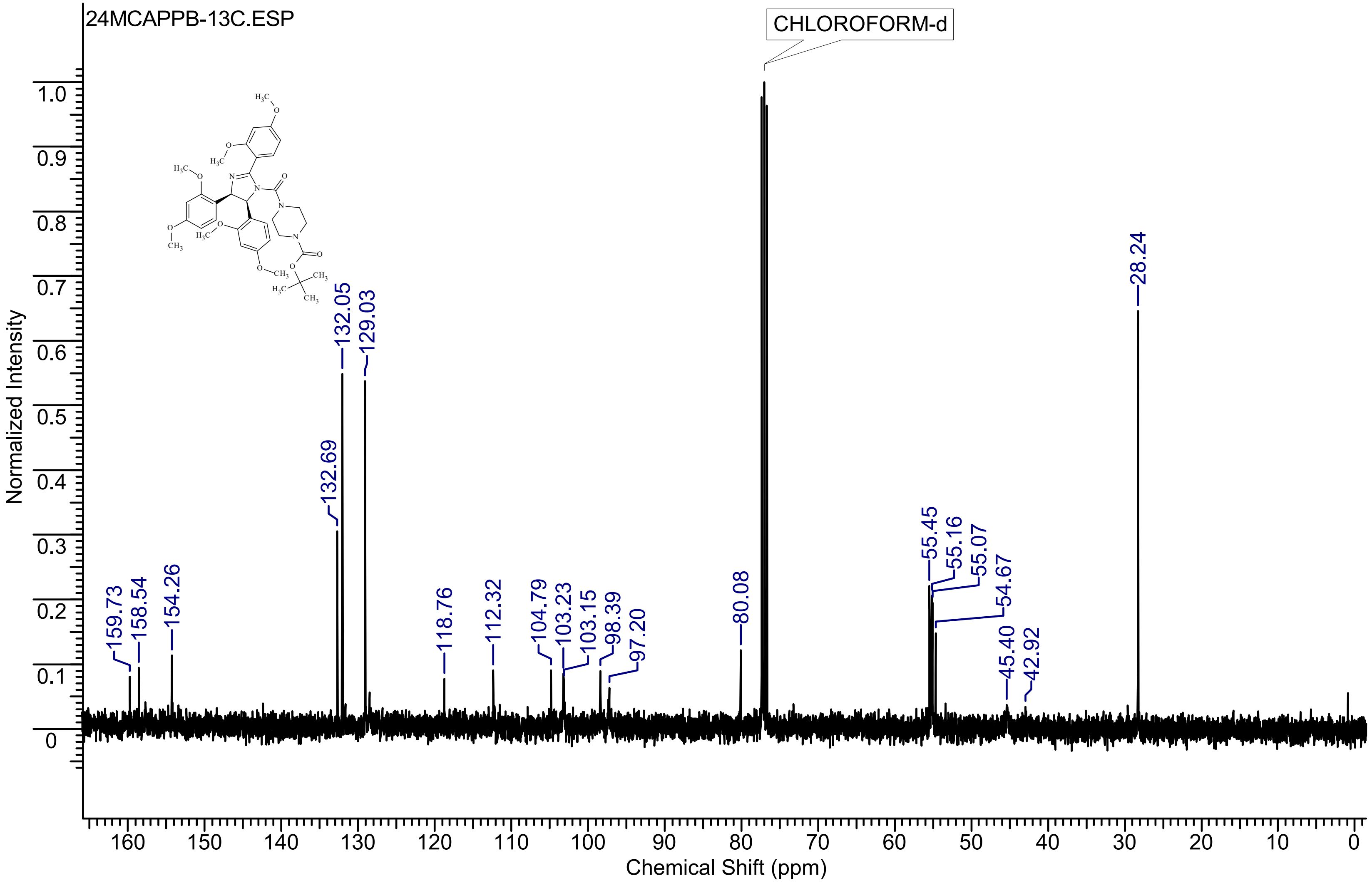

1H NMR *Cis-N,N*-diethyl-2,4,5-*tris*(3,4-dimethoxyphenyl)-4,5-dihydro-*1H*-imidazole-1-carboxamide (2n).

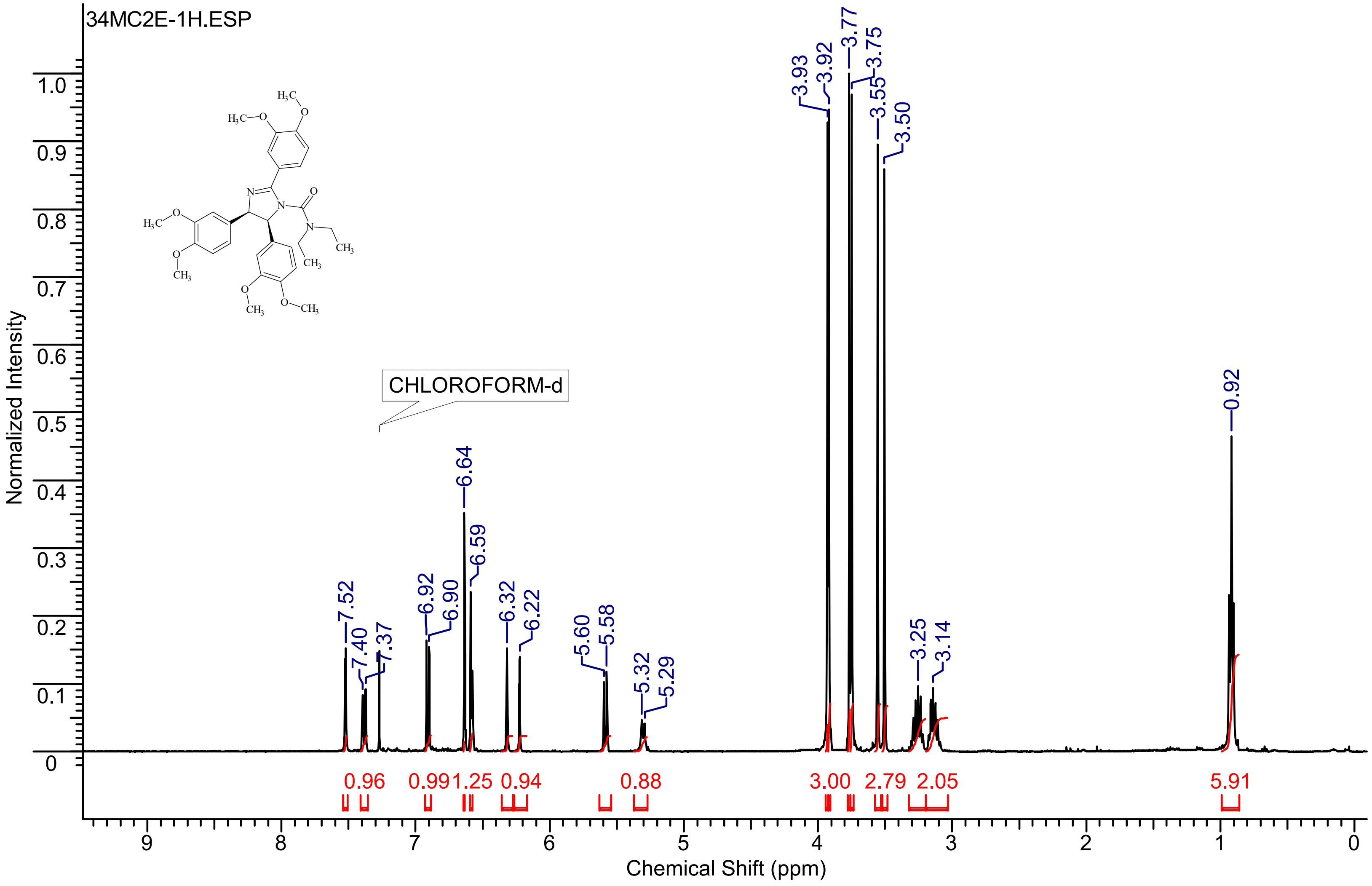

13C NMR *Cis-N,N*-diethyl-2,4,5-*tris*(3,4-dimethoxyphenyl)-4,5-dihydro-*1H*-imidazole-1-carboxamide (2n).

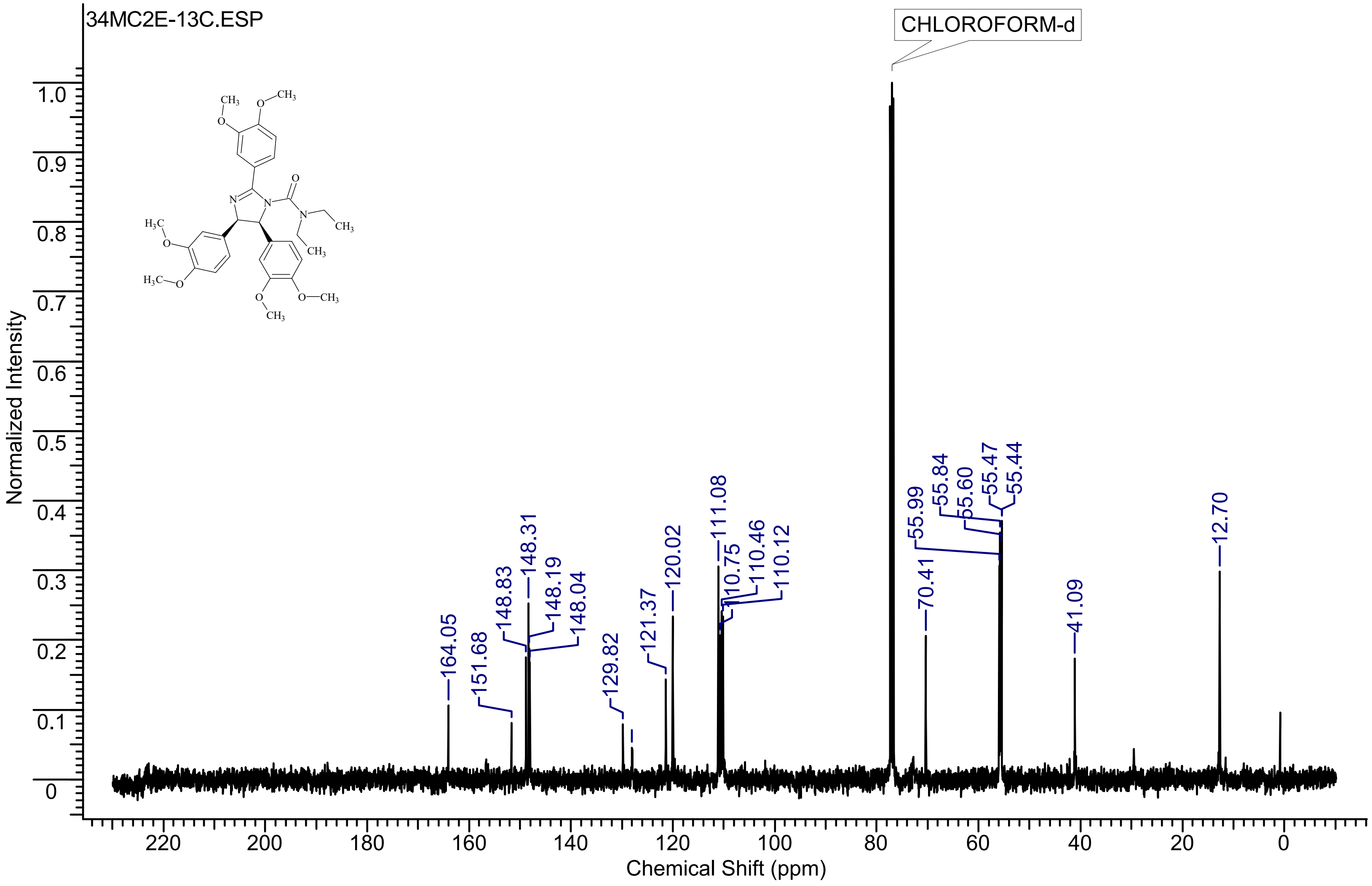

1H NMR 4-{[*Cis*-2,4,5-*tris*(3,4-dimethoxyphenyl)-4,5-dihydro-*1H*-imidazol-1-yl]carbonyl}morpholine (2o).

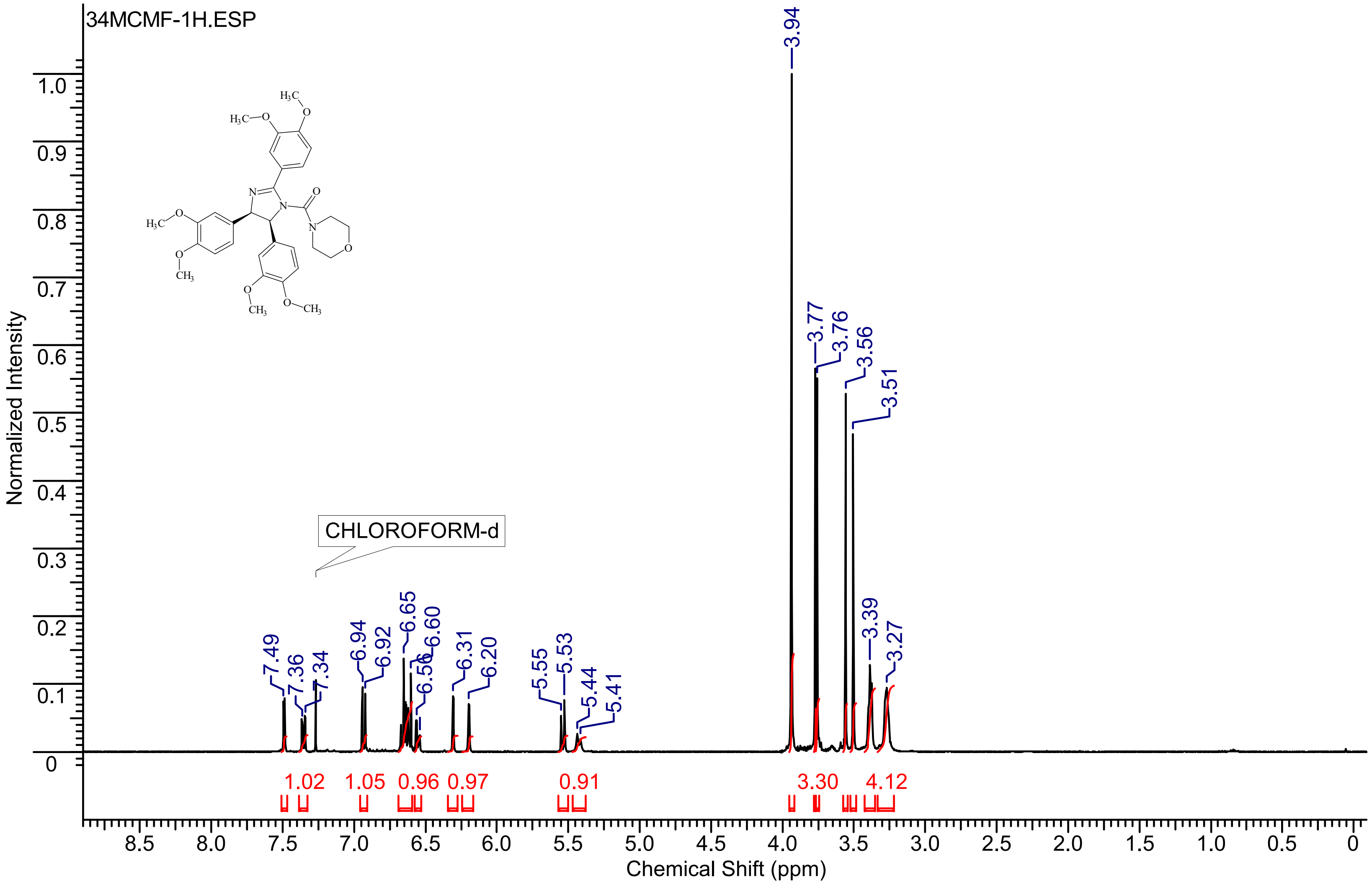

13C NMR 4-{[*Cis*-2,4,5-*tris*(3,4-dimethoxyphenyl)-4,5-dihydro-*1H*-imidazol-1-yl]carbonyl}morpholine (2o).

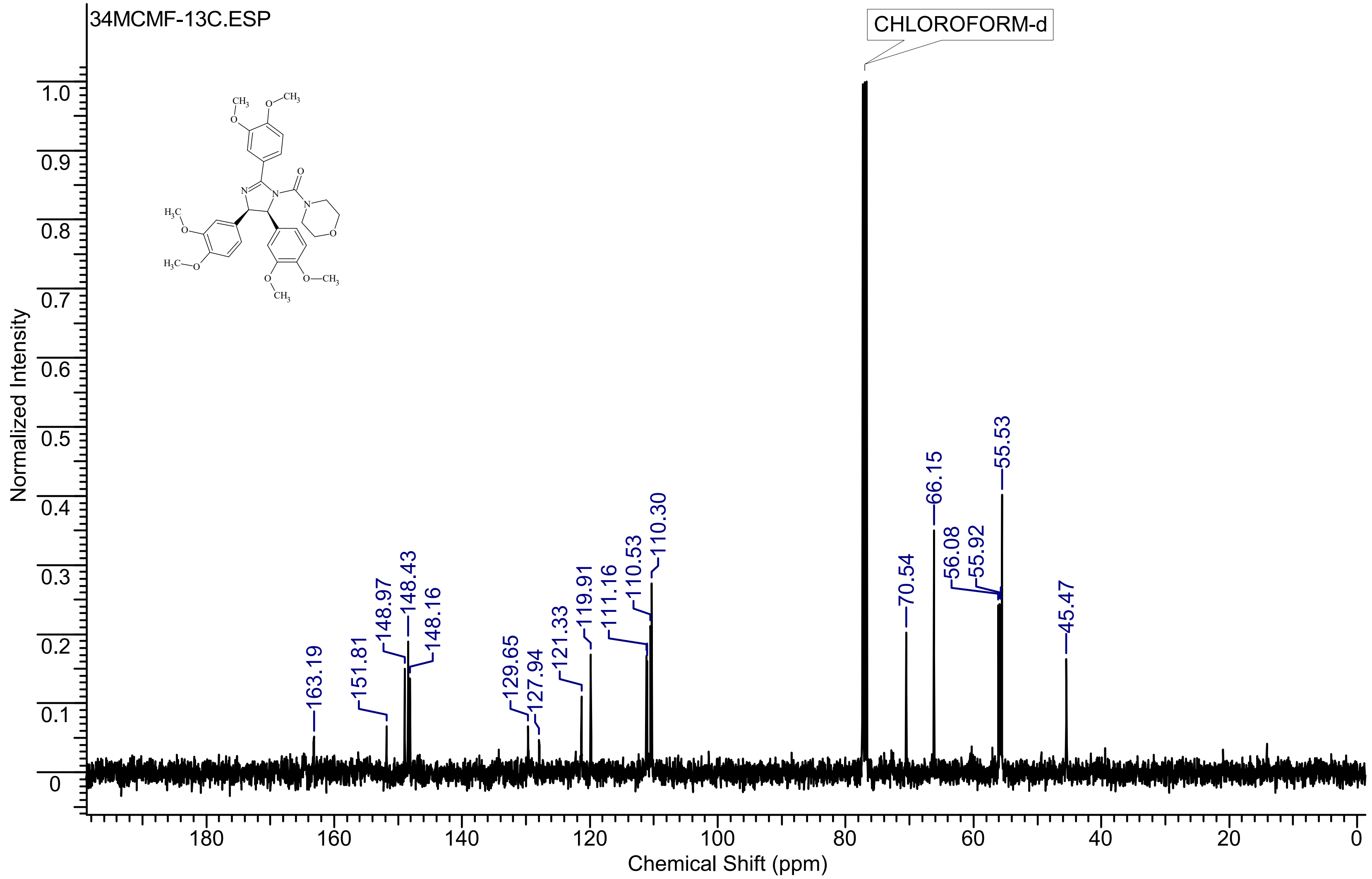

1H NMR Piperidin-1-yl(*cis*-2,4,5-*tris*(3,4-dimethoxyphenyl)-4,5-dihydro-*1H*-imidazol-1-yl)methanone (2p).

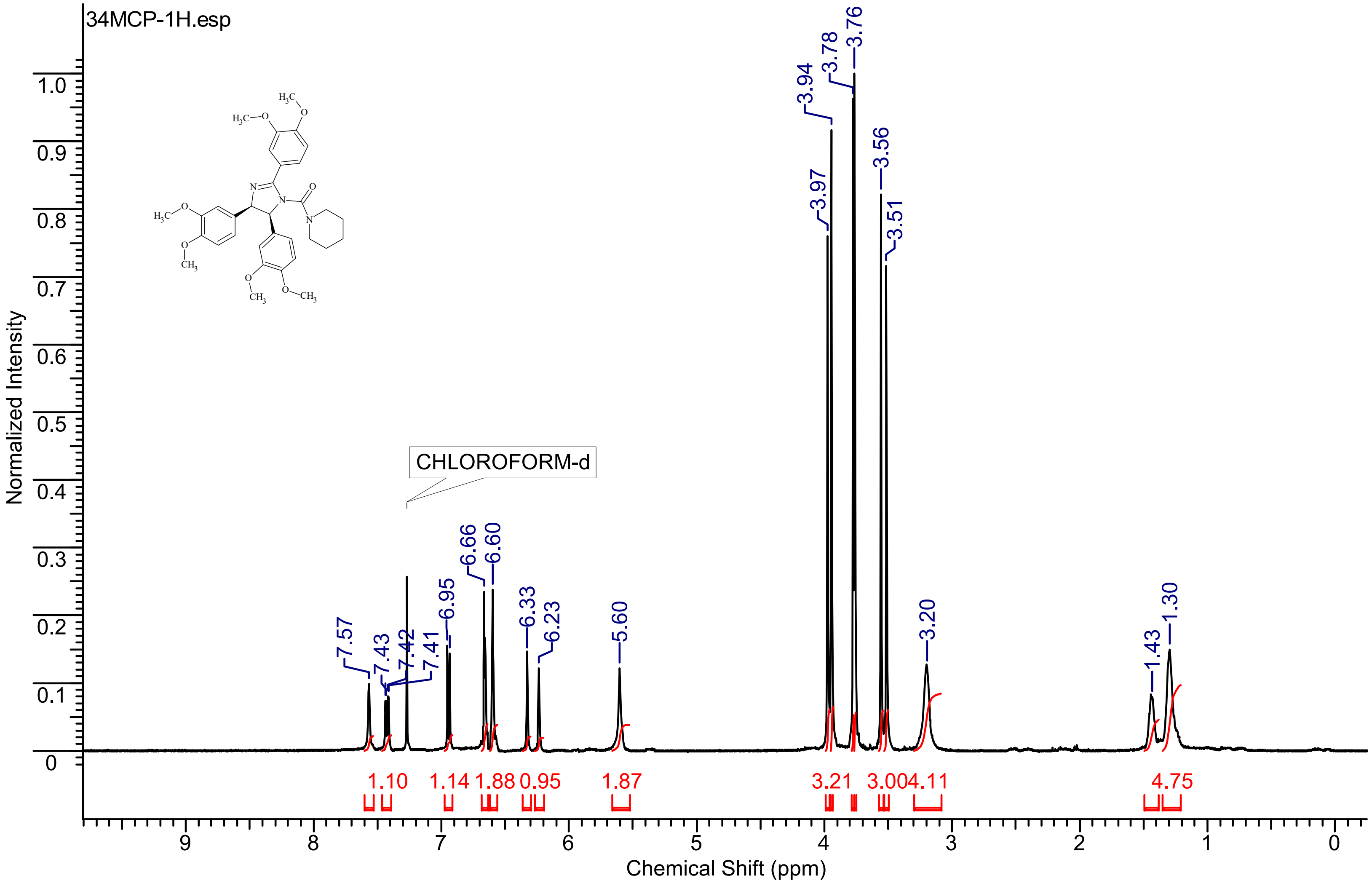

13C NMR Piperidin-1-yl(*cis*-2,4,5-*tris*(3,4-dimethoxyphenyl)-4,5-dihydro-*1H*-imidazol-1-yl)methanone (2p).

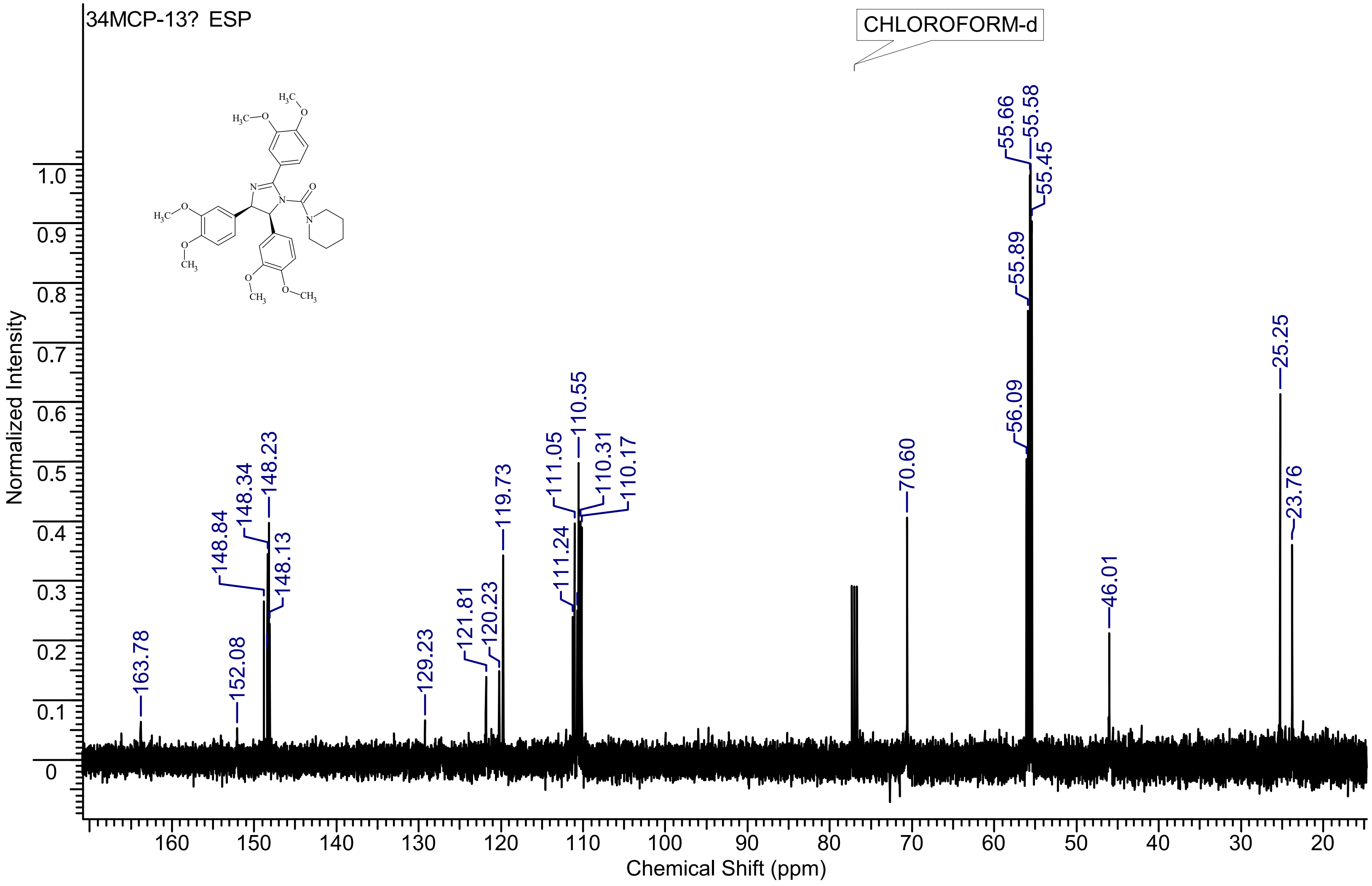

1H NMR (4-Methylpiperidin-1-yl)(*cis*-2,4,5-*tris*(3,4-dimethoxyphenyl)-4,5-dihydro-*1H*-imidazol-1-yl)methanone (2q).

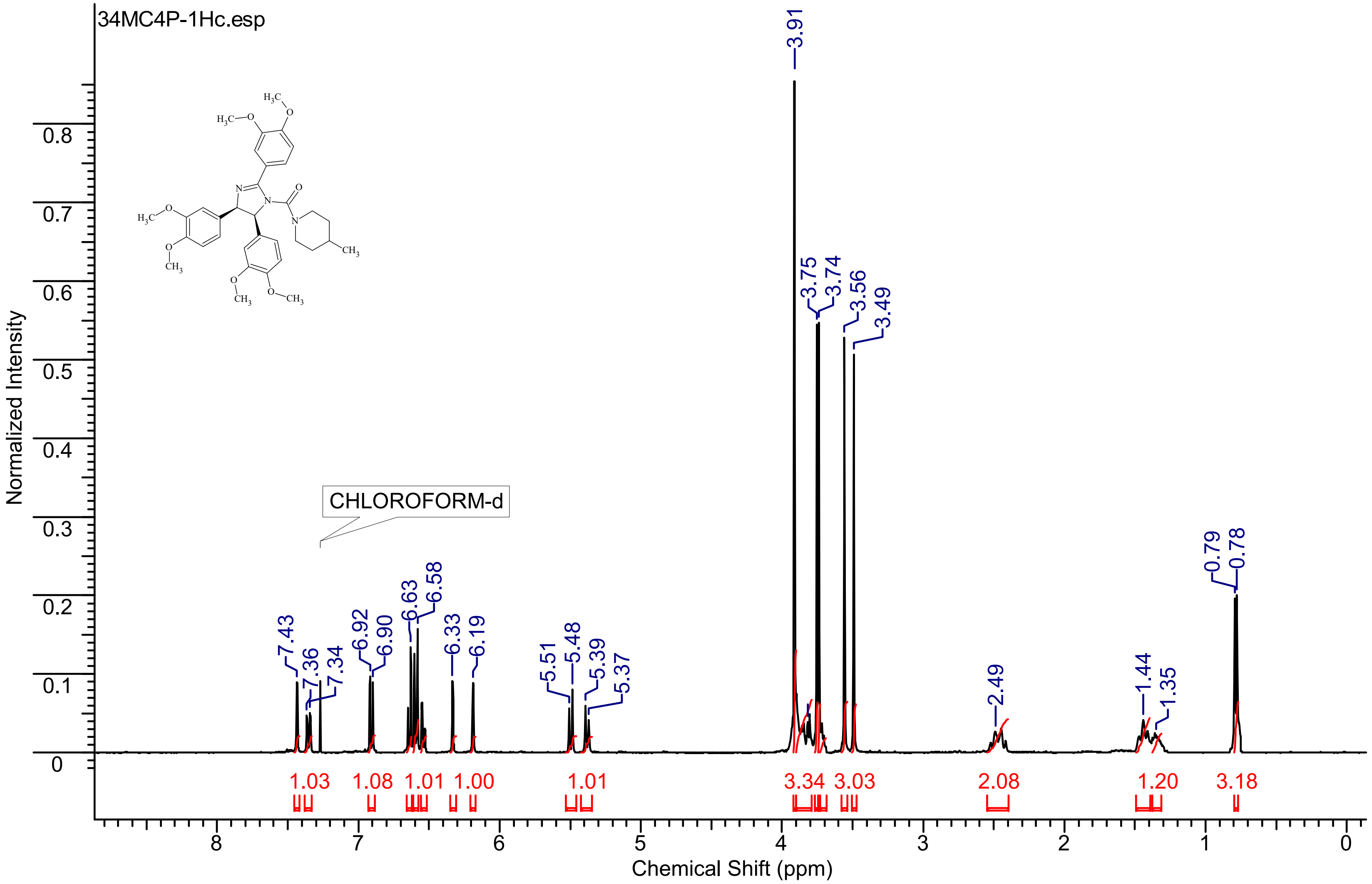

13C NMR (4-Methylpiperidin-1-yl)(*cis*-2,4,5-*tris*(3,4-dimethoxyphenyl)-4,5-dihydro-*1H*-imidazol-1-yl)methanone (2q).

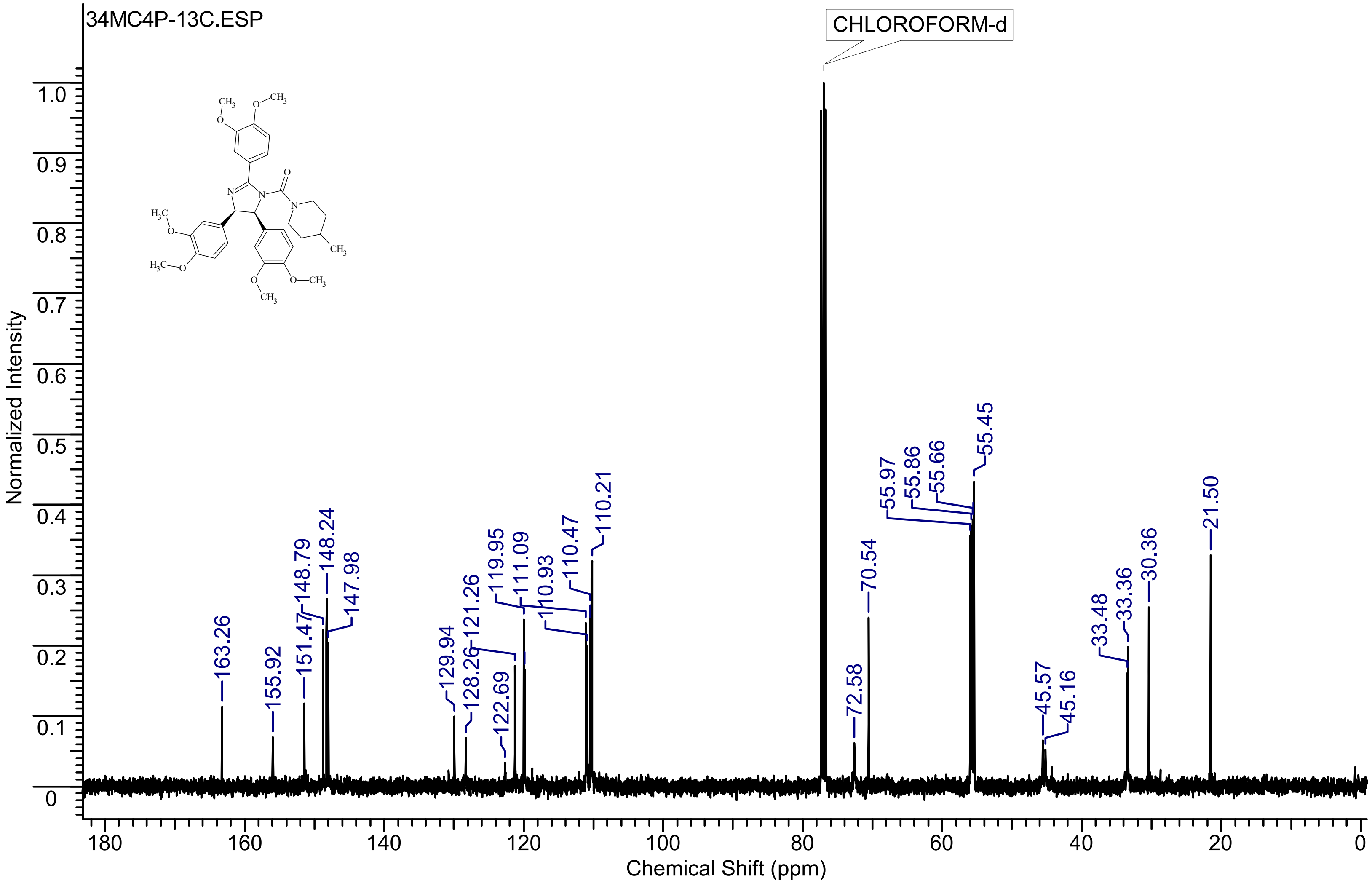

1H NMR Pyrrolidin-1-yl(*cis*-2,4,5-*tris*(3,4-dimethoxyphenyl)-4,5-dihydro-*1H*-imidazol-1-yl)methanone (2r).

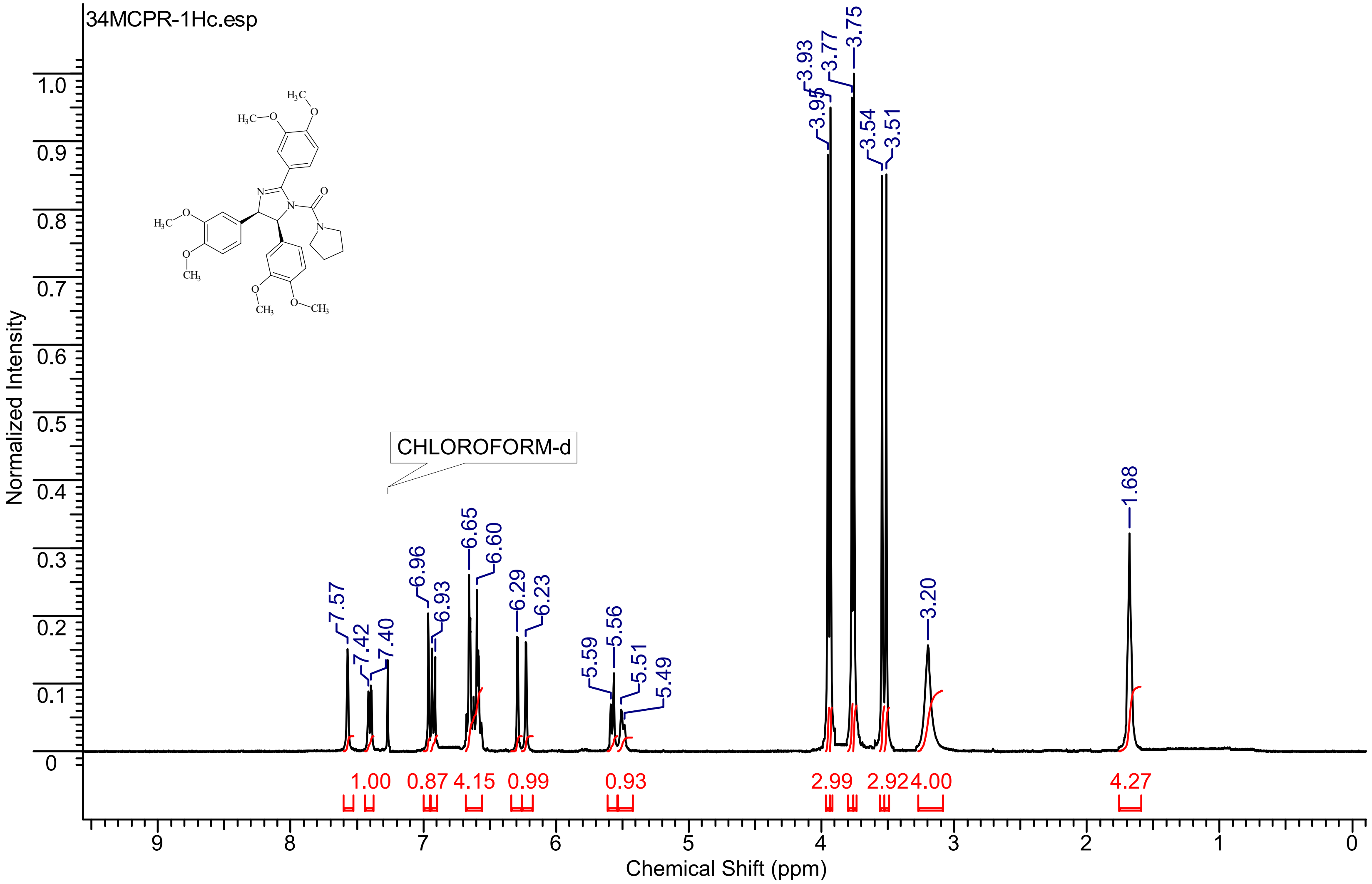

13C NMR Pyrrolidin-1-yl(*cis*-2,4,5-*tris*(3,4-dimethoxyphenyl)-4,5-dihydro-*1H*-imidazol-1-yl)methanone (2r).

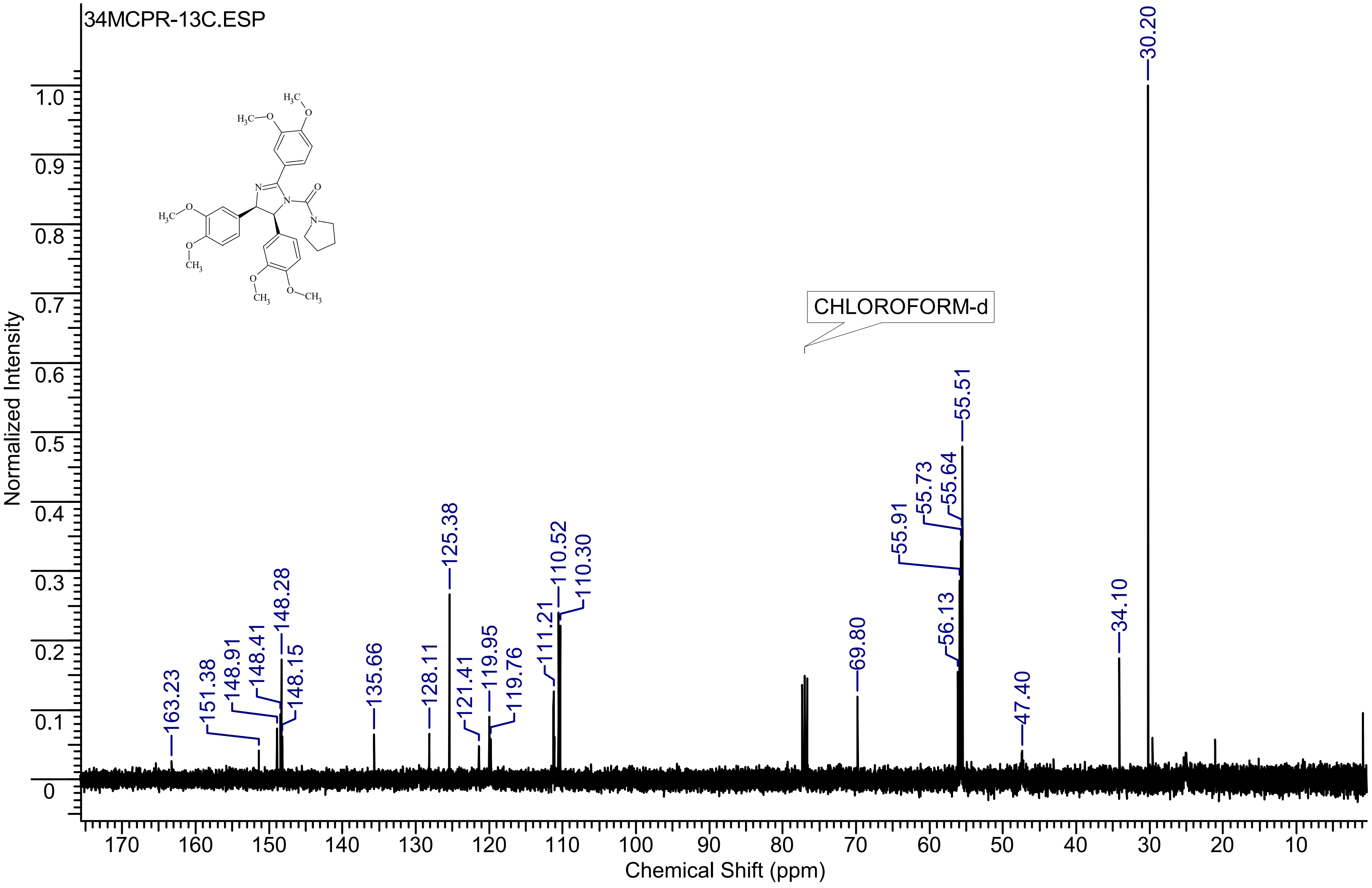

1H NMR Azepan-1-yl(*cis*-2,4,5-*tris*(3,4-dimethoxyphenyl)-4,5-dihydro-*1H*-imidazol-1-yl)methanone (2s).

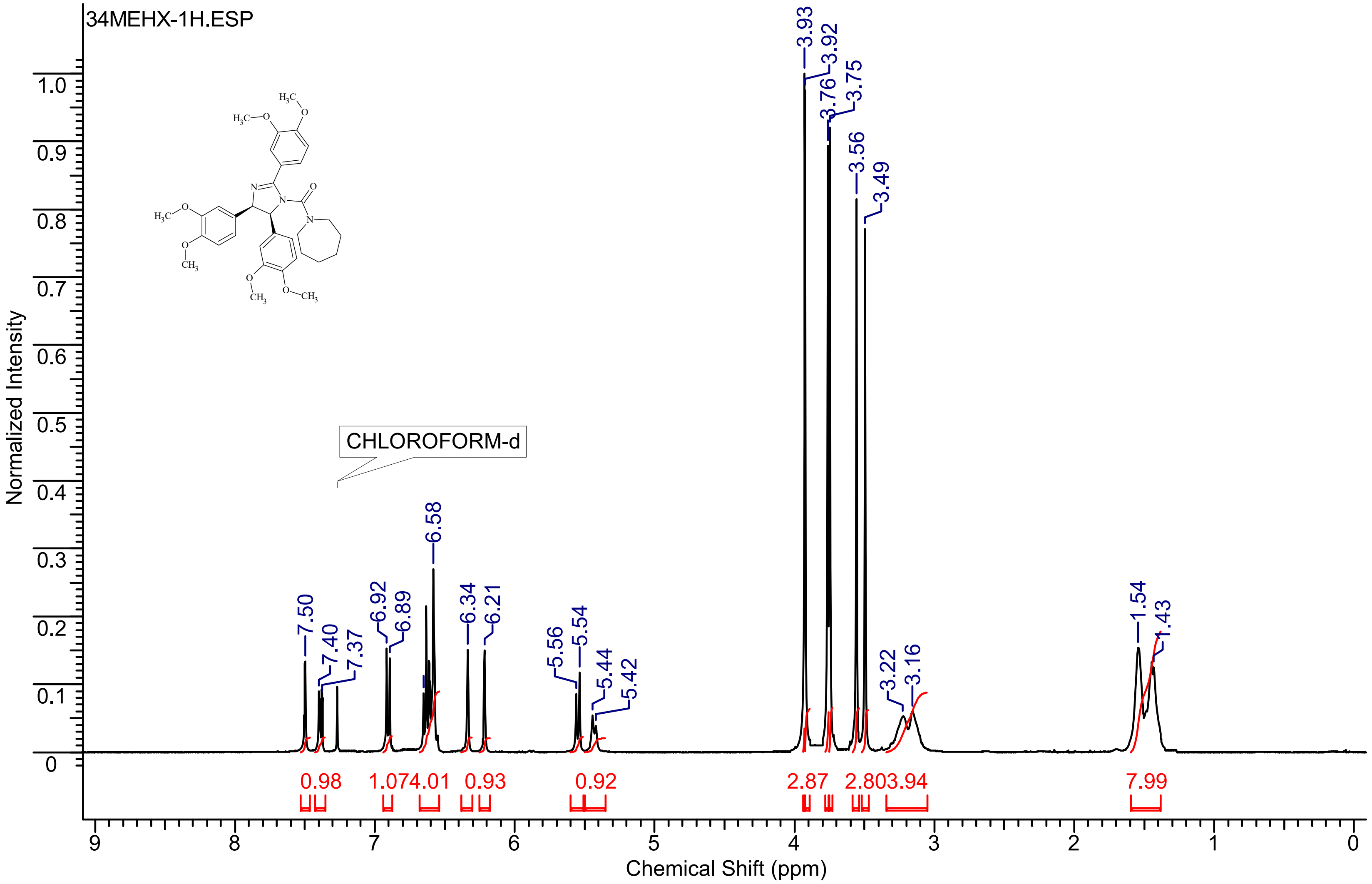

13C NMR Azepan-1-yl(*cis*-2,4,5-*tris*(3,4-dimethoxyphenyl)-4,5-dihydro-*1H*-imidazol-1-yl)methanone (2s).

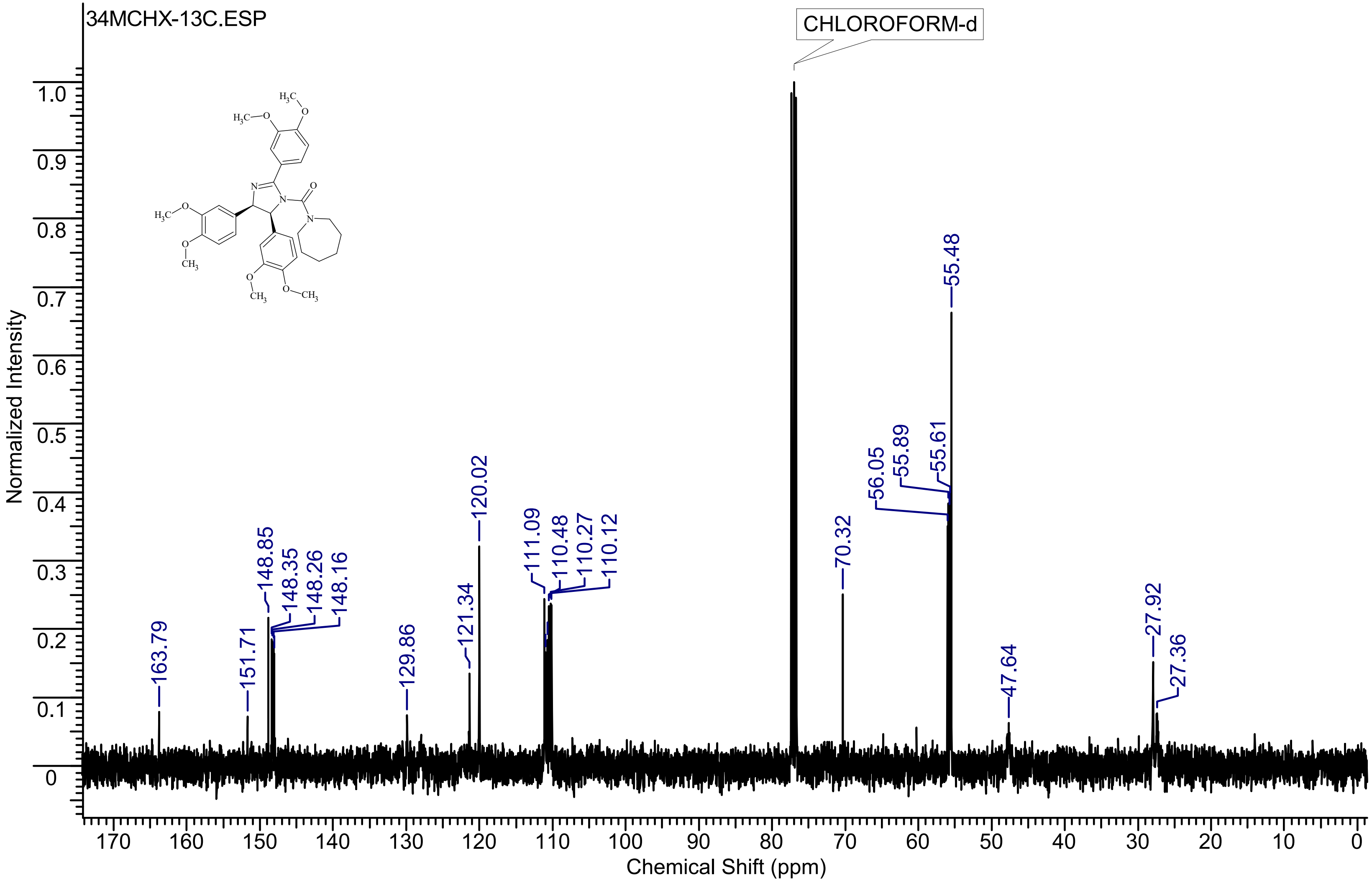

1H NMR (4-Methylpiperazin-1-yl)(*cis*-2,4,5-*tris*(3,4-dimethoxyphenyl)-4,5-dihydro-*1H*-imidazol-1-yl)methanone (2t).

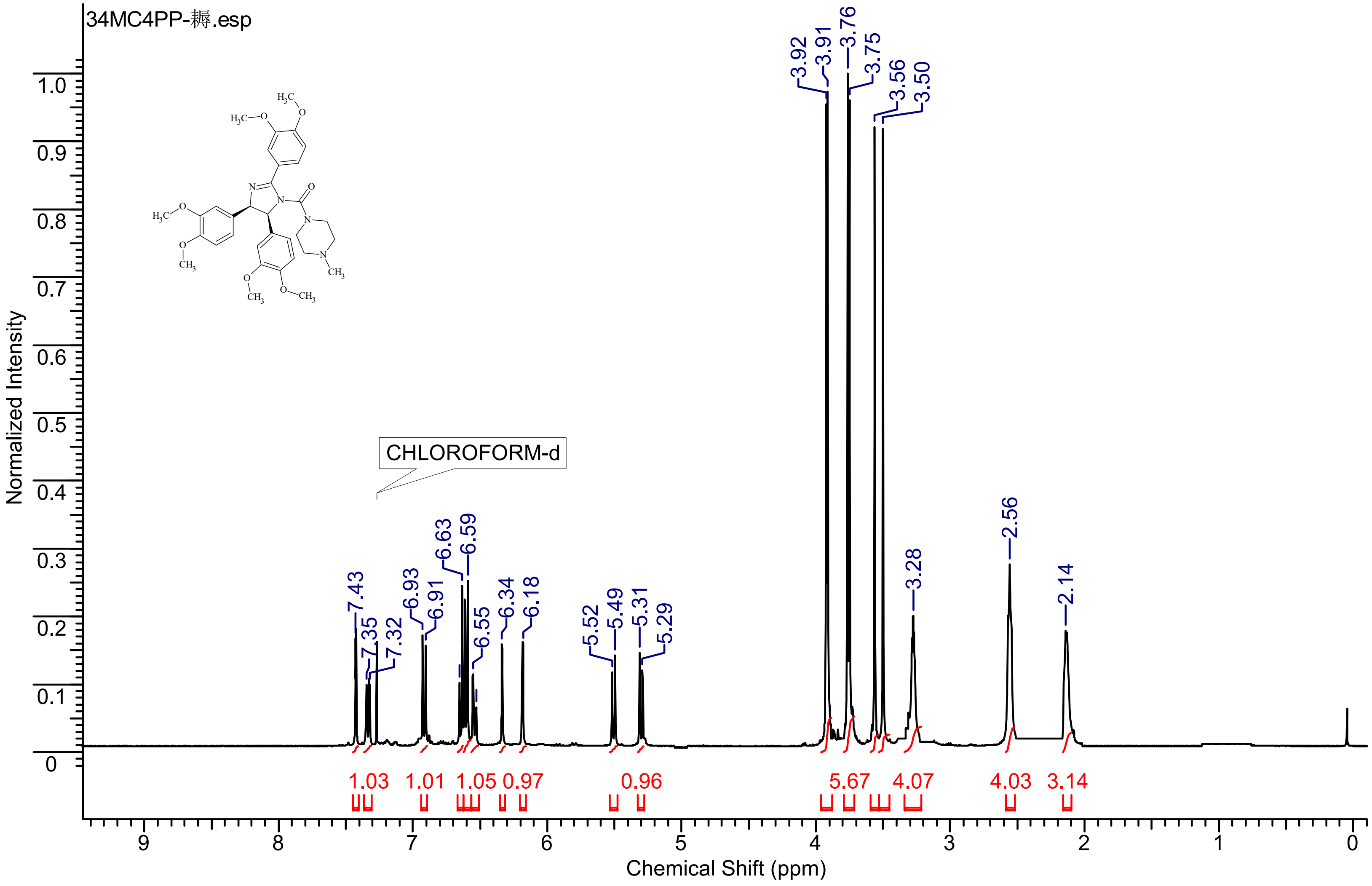

13C NMR (4-Methylpiperazin-1-yl)(*cis*-2,4,5-*tris*(3,4-dimethoxyphenyl)-4,5-dihydro-*1H*-imidazol-1-yl)methanone (2t).

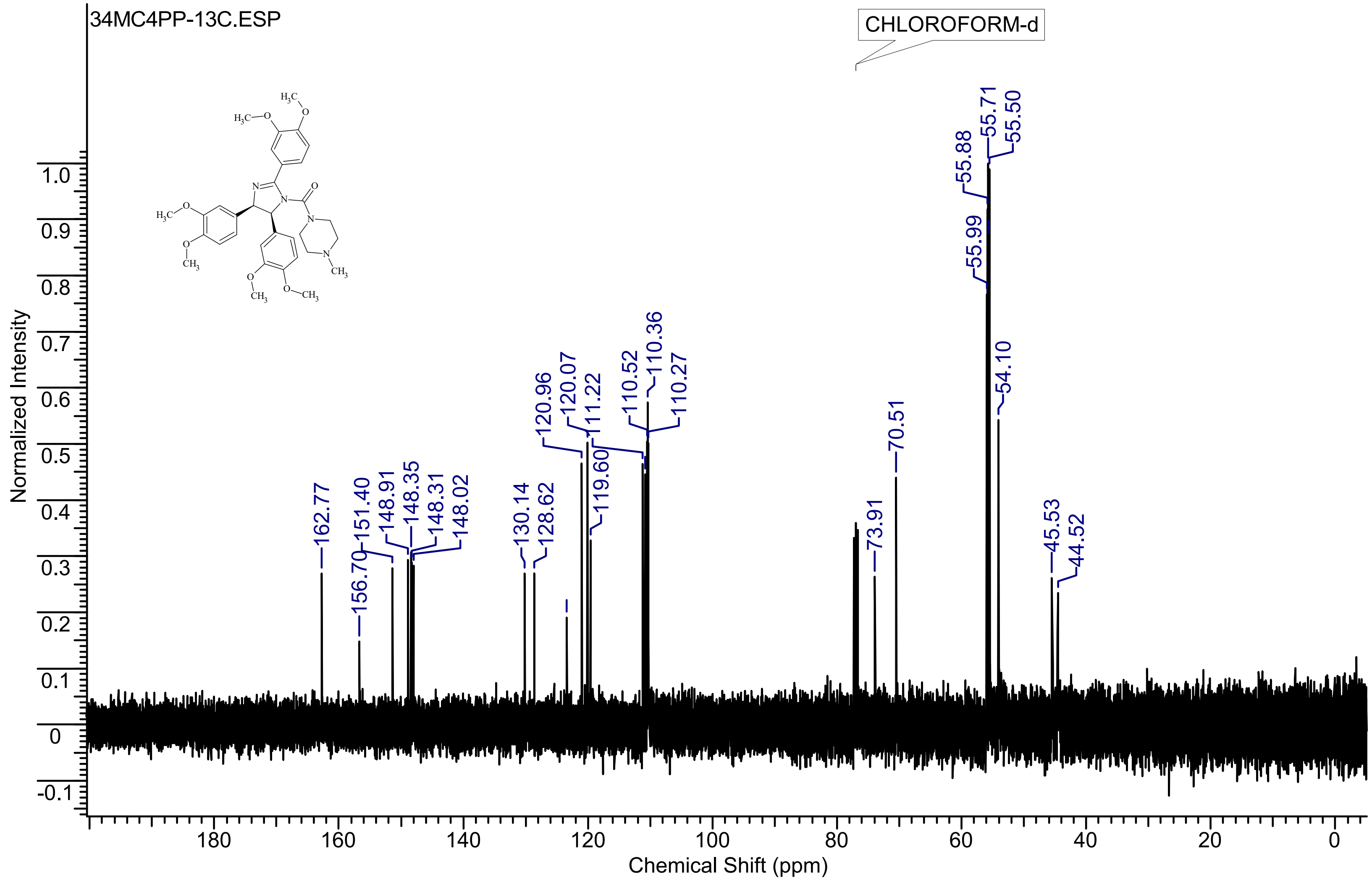

***1H NMR Cis-N,N*-diethyl-2,4,5-*tris*(3,5-dimethoxyphenyl)-4,5-dihydro-*1H*-imidazole-1-carboxamide (2u).**

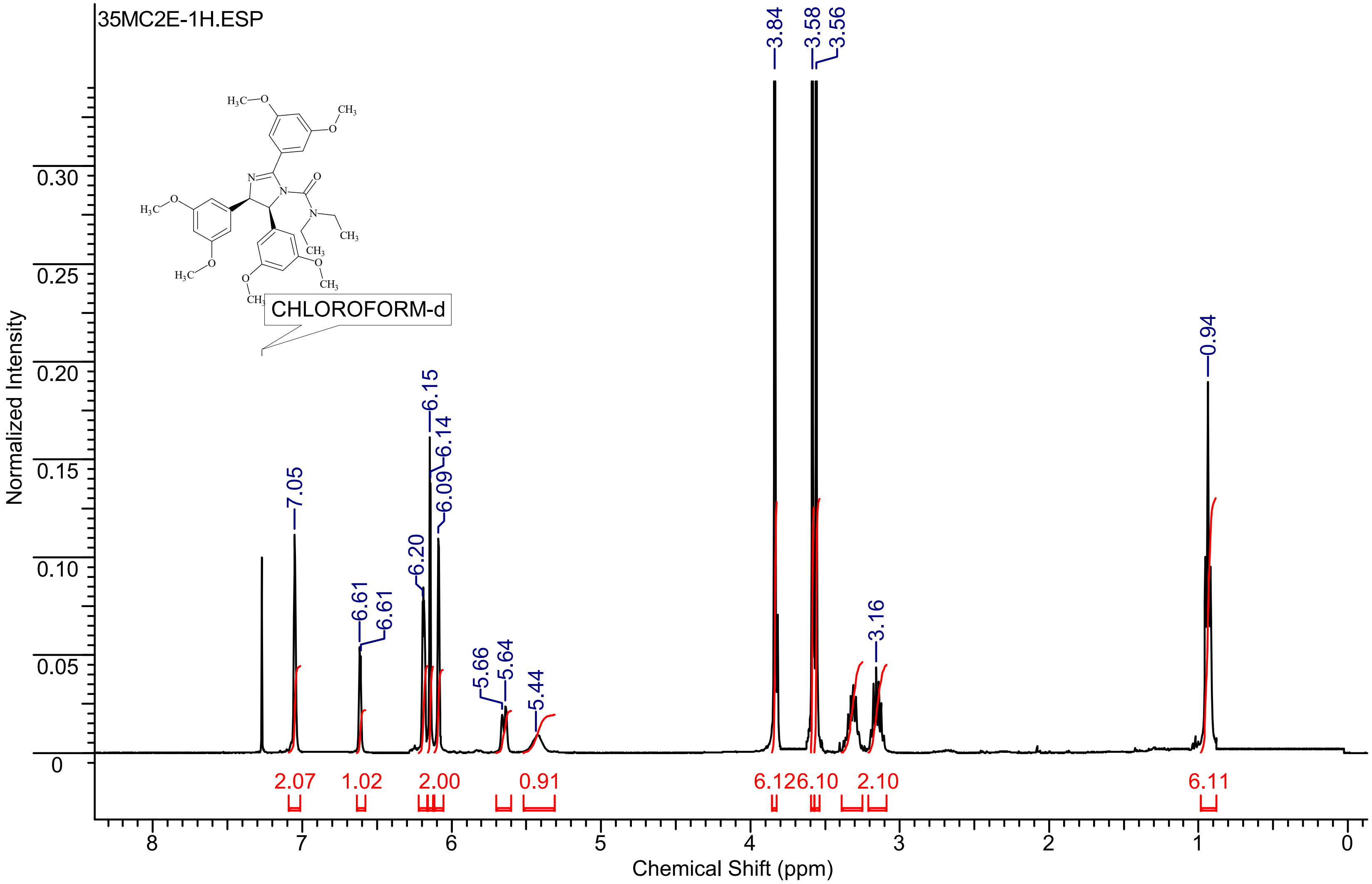

***13C NMR Cis-N,N*-diethyl-2,4,5-*tris*(3,5-dimethoxyphenyl)-4,5-dihydro-*1H*-imidazole-1-carboxamide (2u).**

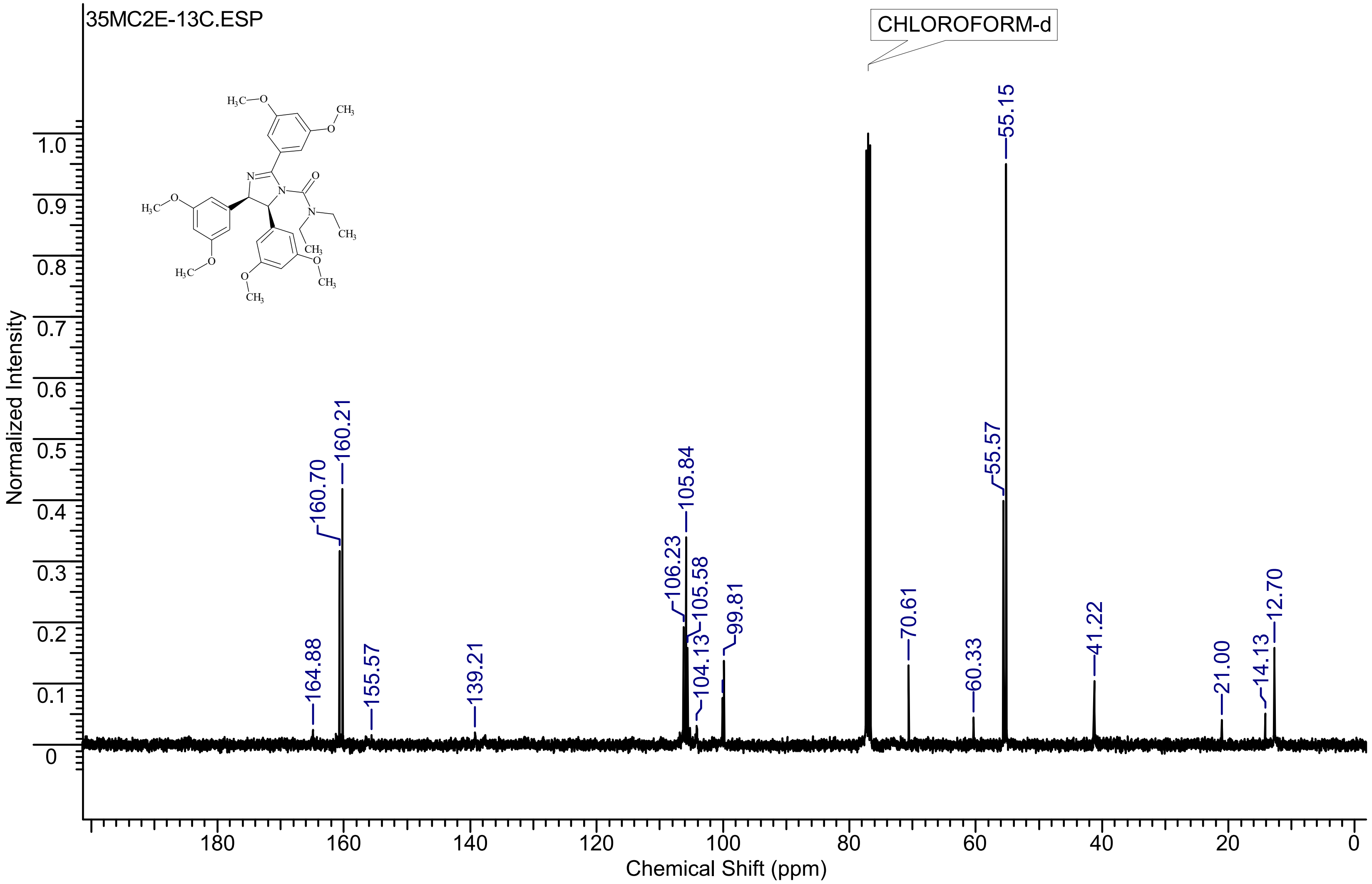

1H NMR 4-{[*Cis*-2,4,5-*tris*(3,5-dimethoxyphenyl)-4,5-dihydro-*1H*-imidazol-1-yl]carbonyl}morpholine (2v).

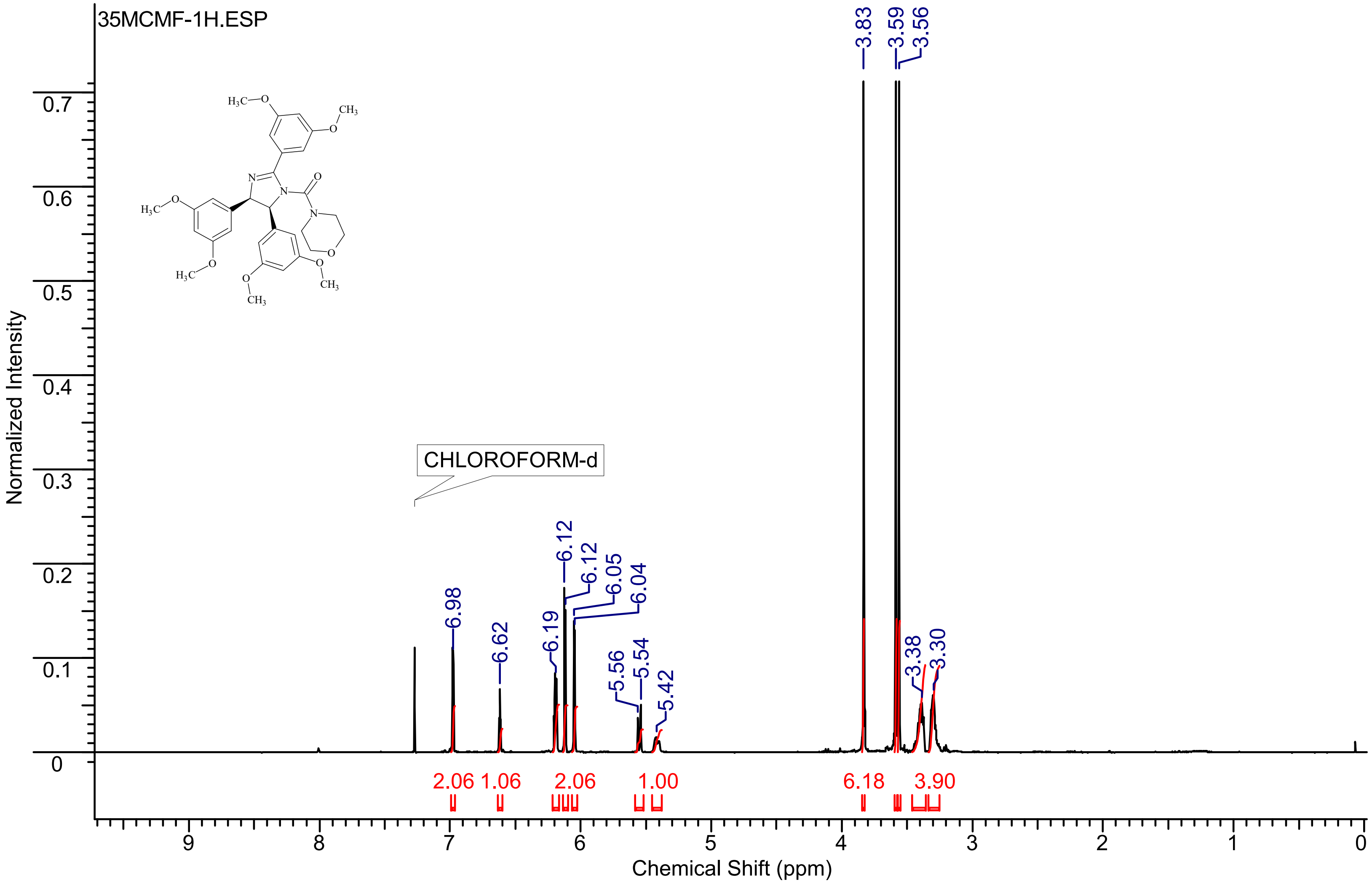

13C NMR 4-{[*Cis*-2,4,5-*tris*(3,5-dimethoxyphenyl)-4,5-dihydro-*1H*-imidazol-1-yl]carbonyl}morpholine (2v).

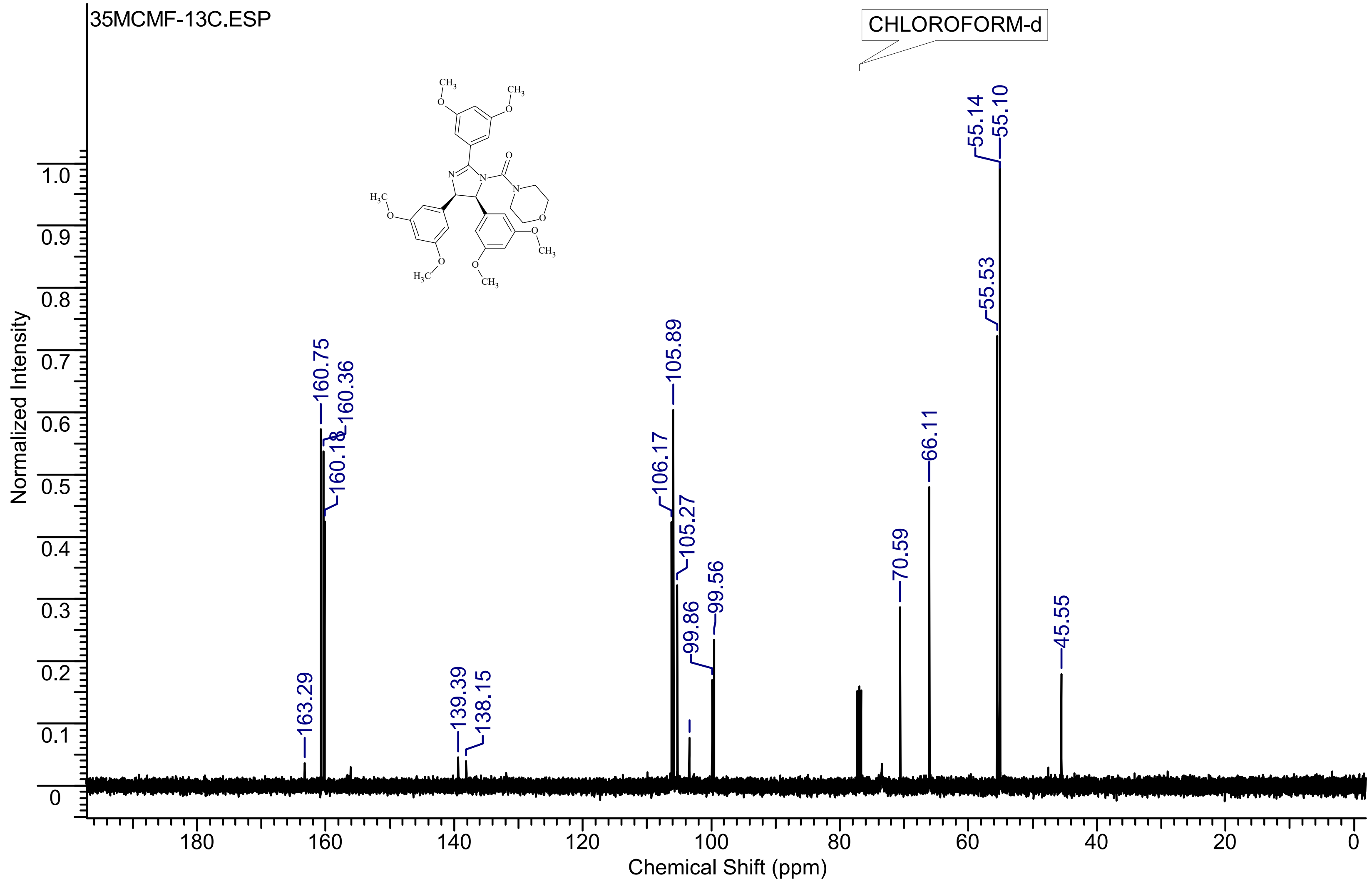

1H NMR (4-Methylpiperazin-1-yl)(*cis*-2,4,5-*tris*(3,5-dimethoxyphenyl)-4,5-dihydro-*1H*-imidazol-1-yl)methanone (2w).

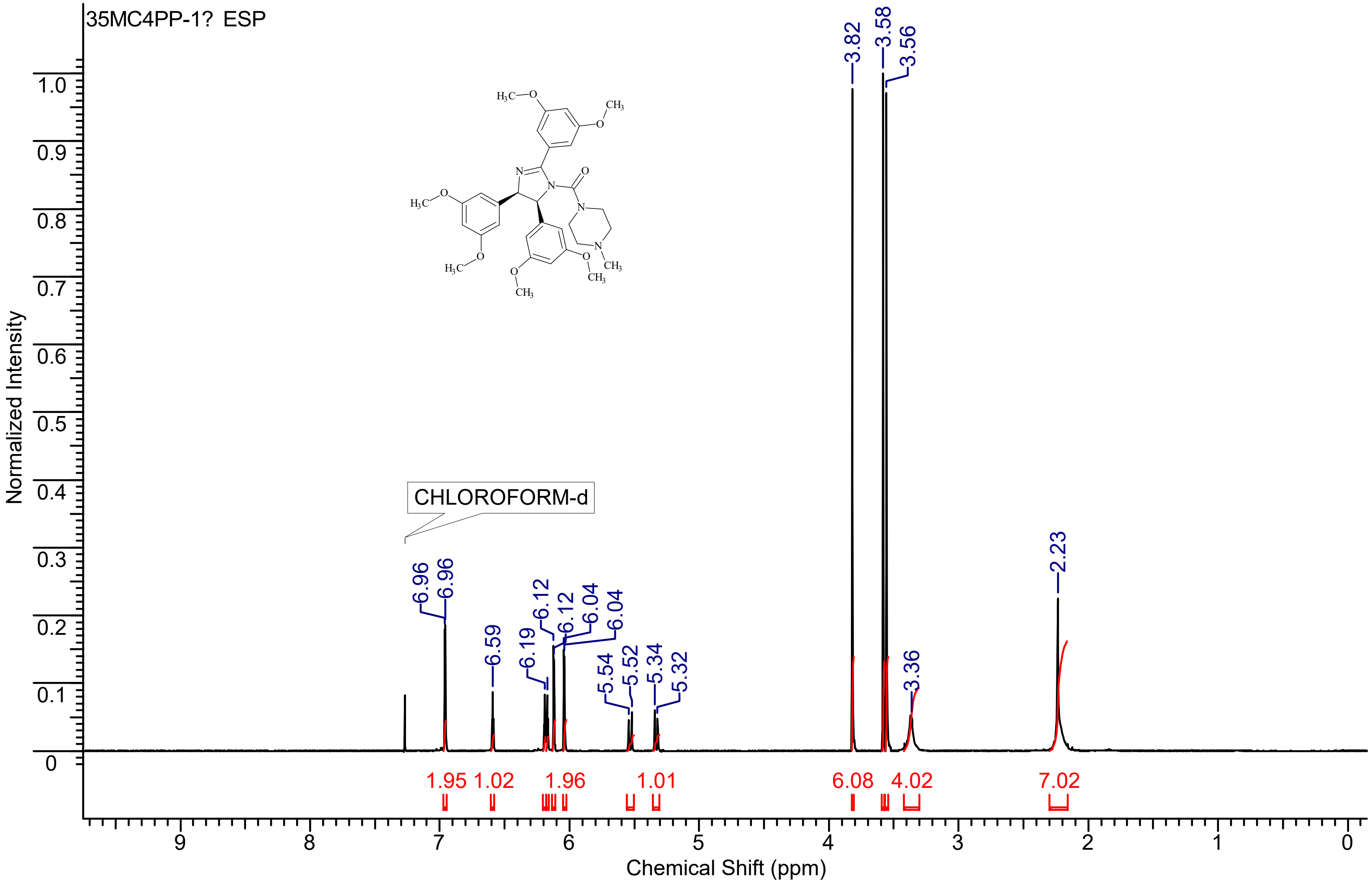

13C NMR (4-Methylpiperazin-1-yl)(*cis*-2,4,5-*tris*(3,5-dimethoxyphenyl)-4,5-dihydro-*1H*-imidazol-1-yl)methanone (2w).

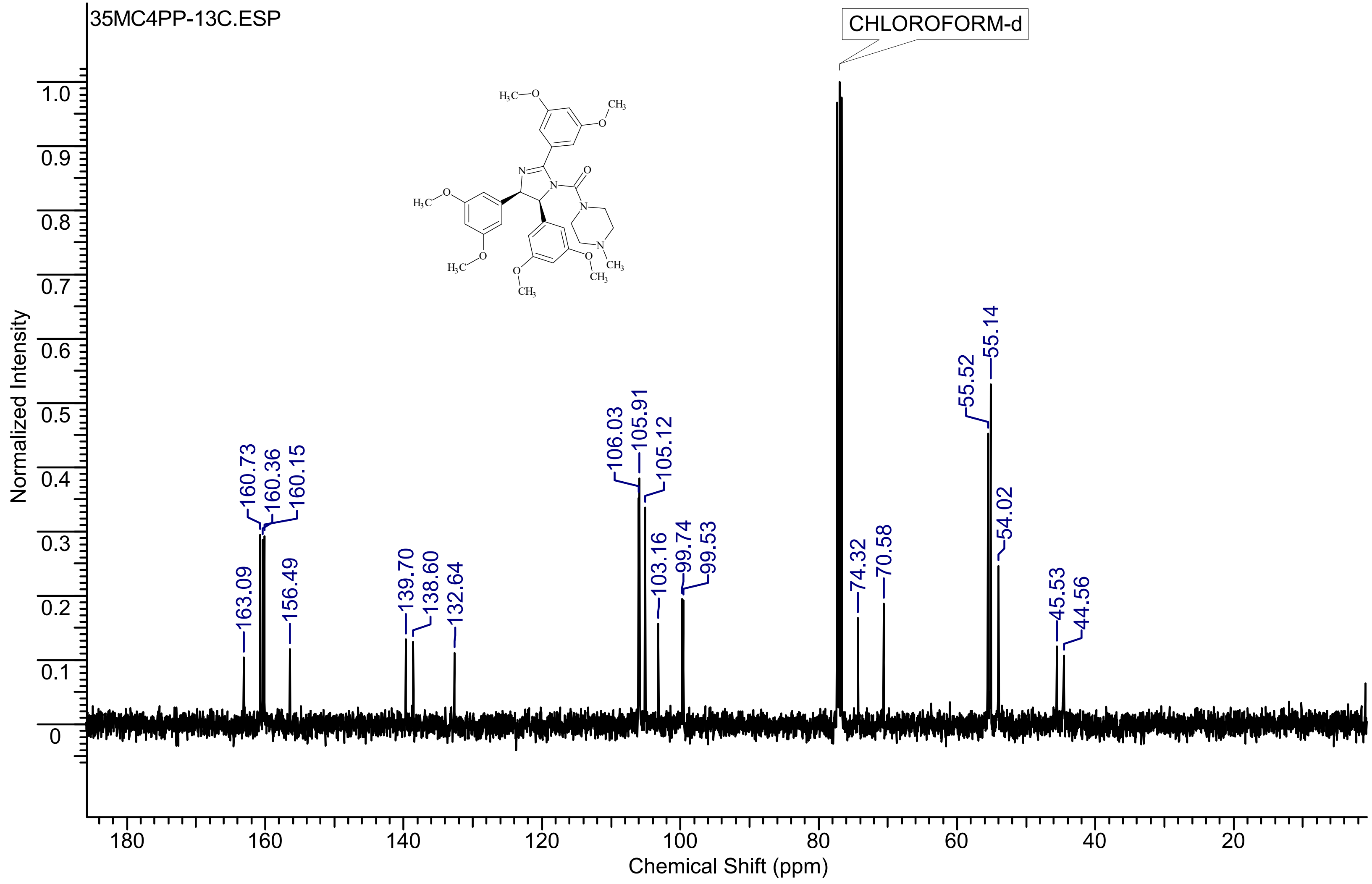

1H NMR Piperidin-1-yl(*cis*-2,4,5-*tris*(4-chlorophenyl)-4,5-dihydro-*1H*-imidazol-1-yl)methanone (2x).

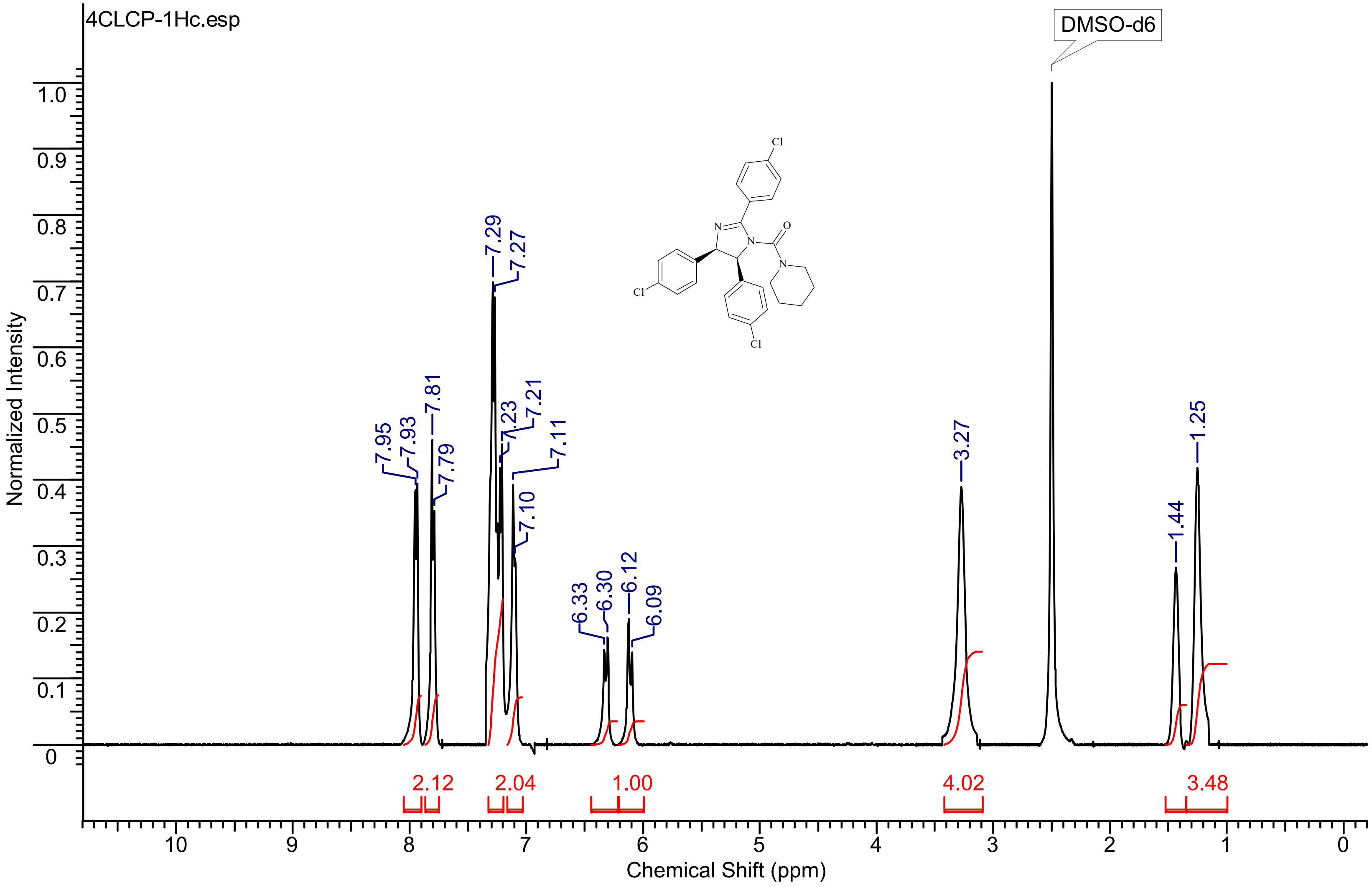

13C NMR Piperidin-1-yl(*cis*-2,4,5-*tris*(4-chlorophenyl)-4,5-dihydro-*1H*-imidazol-1-yl)methanone (2x).

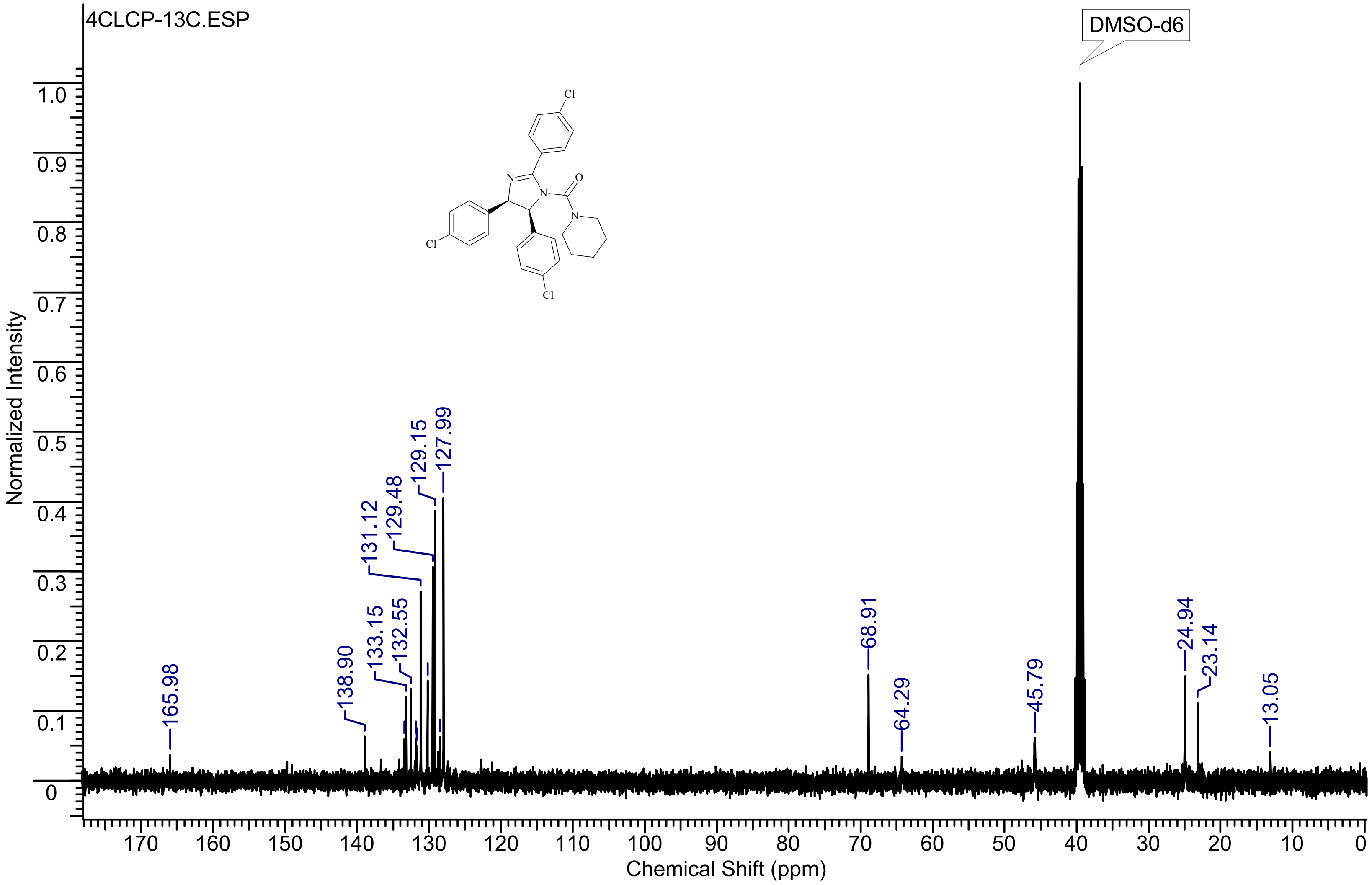

1H NMR *Cis-N,N*-diethyl-2,4,5-*tris*(2,4-dichlorophenyl)-4,5-dihydro-*1H*-imidazole-1-carboxamide (2y).

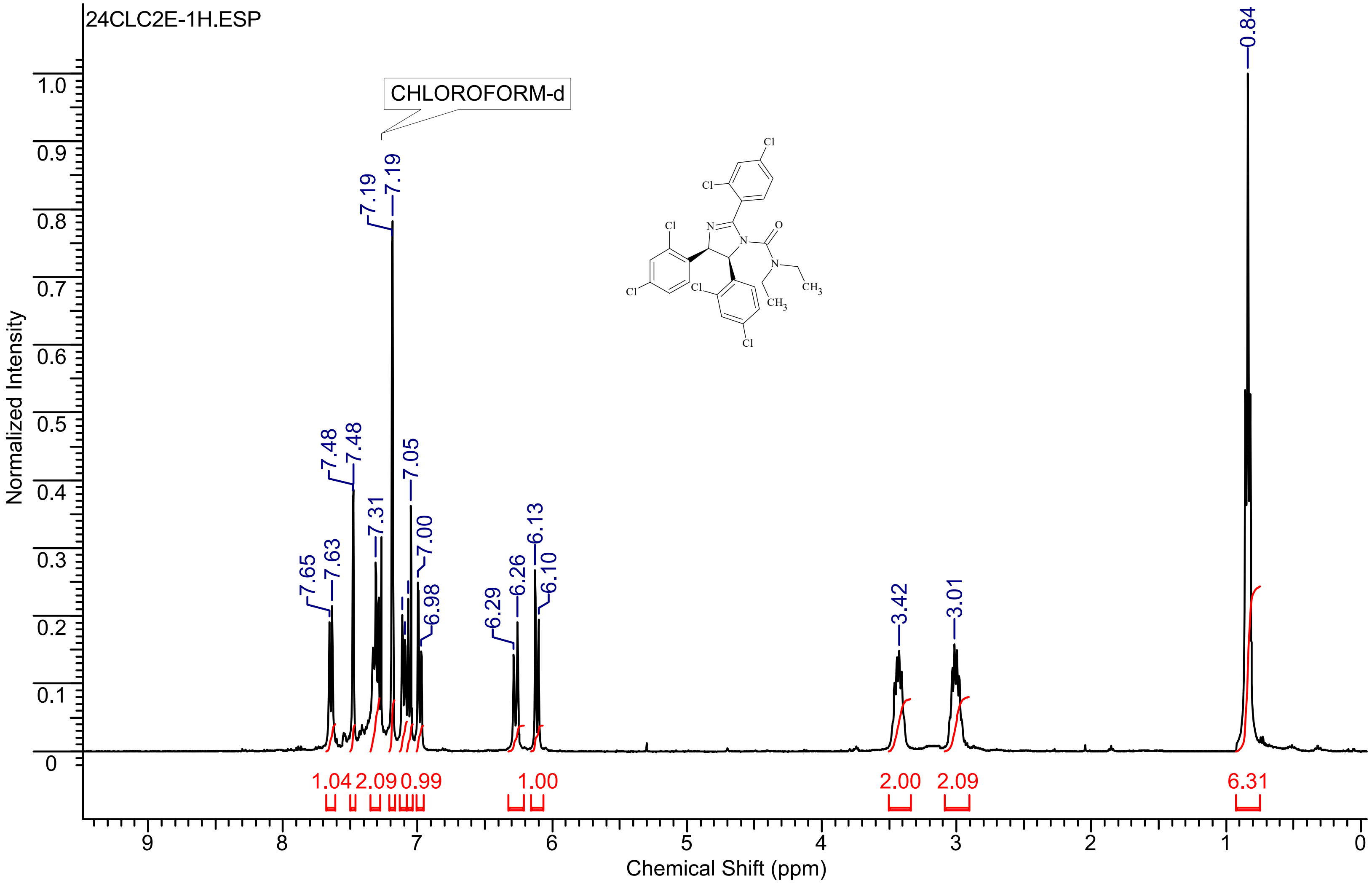

13C NMR *Cis-N,N*-diethyl-2,4,5-*tris*(2,4-dichlorophenyl)-4,5-dihydro-*1H*-imidazole-1-carboxamide (2y).

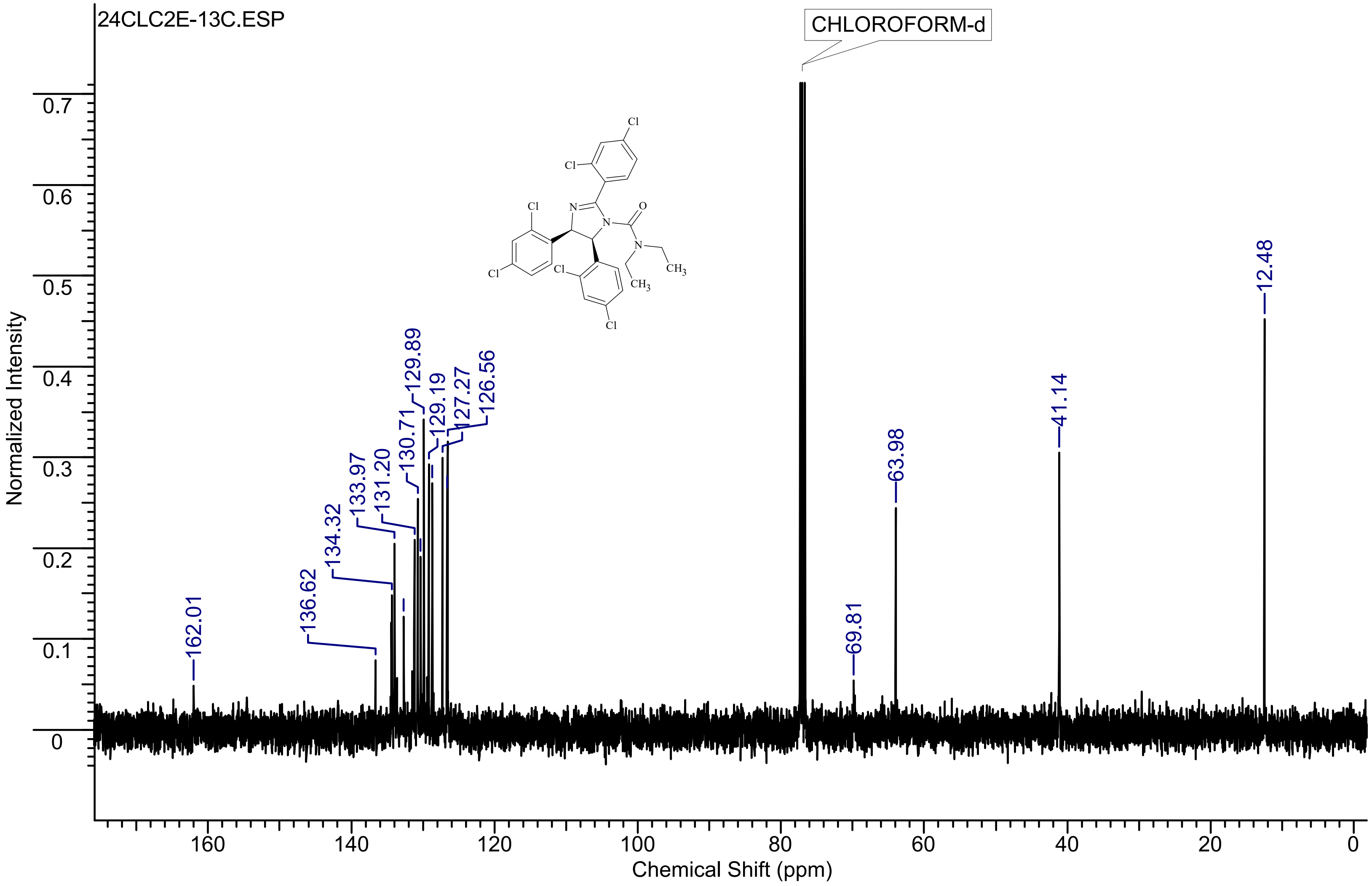

1H NMR 4-{[*Cis*-2,4,5-*tris*(2,4-dichlorophenyl)-4,5-dihydro-*1H*-imidazol-1-yl]carbonyl}morpholine (2z).

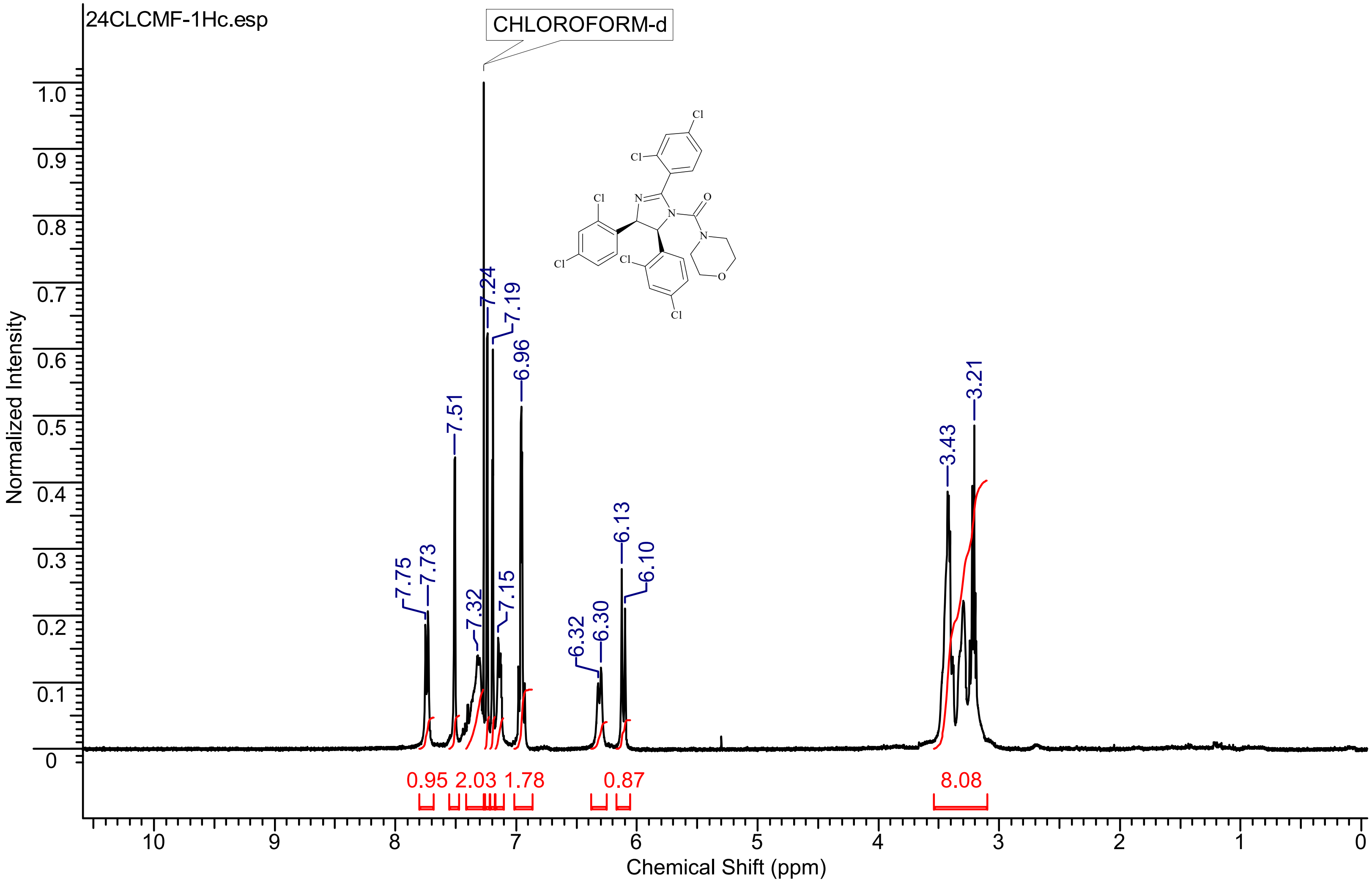

13C NMR 4-{[*Cis*-2,4,5-*tris*(2,4-dichlorophenyl)-4,5-dihydro-*1H*-imidazol-1-yl]carbonyl}morpholine (2z).

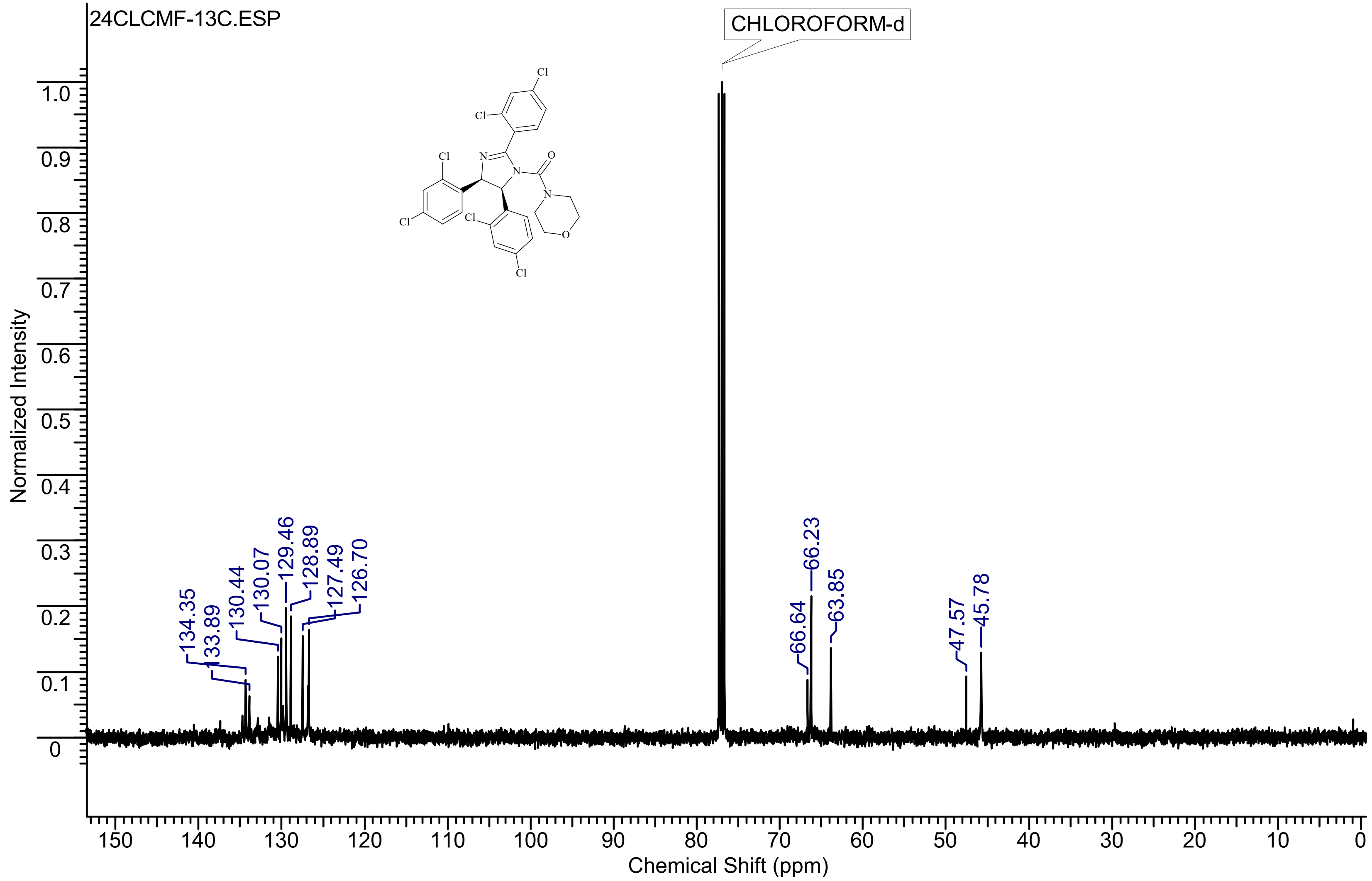

1H NMR *tert*-Butyl 4-(*cis*-2,4,5-*tris*(2,4-dichlorophenyl)-4,5-dihydro-*1H*-imidazole-1-carbonyl)piperazine-1-carboxylate (2aa).

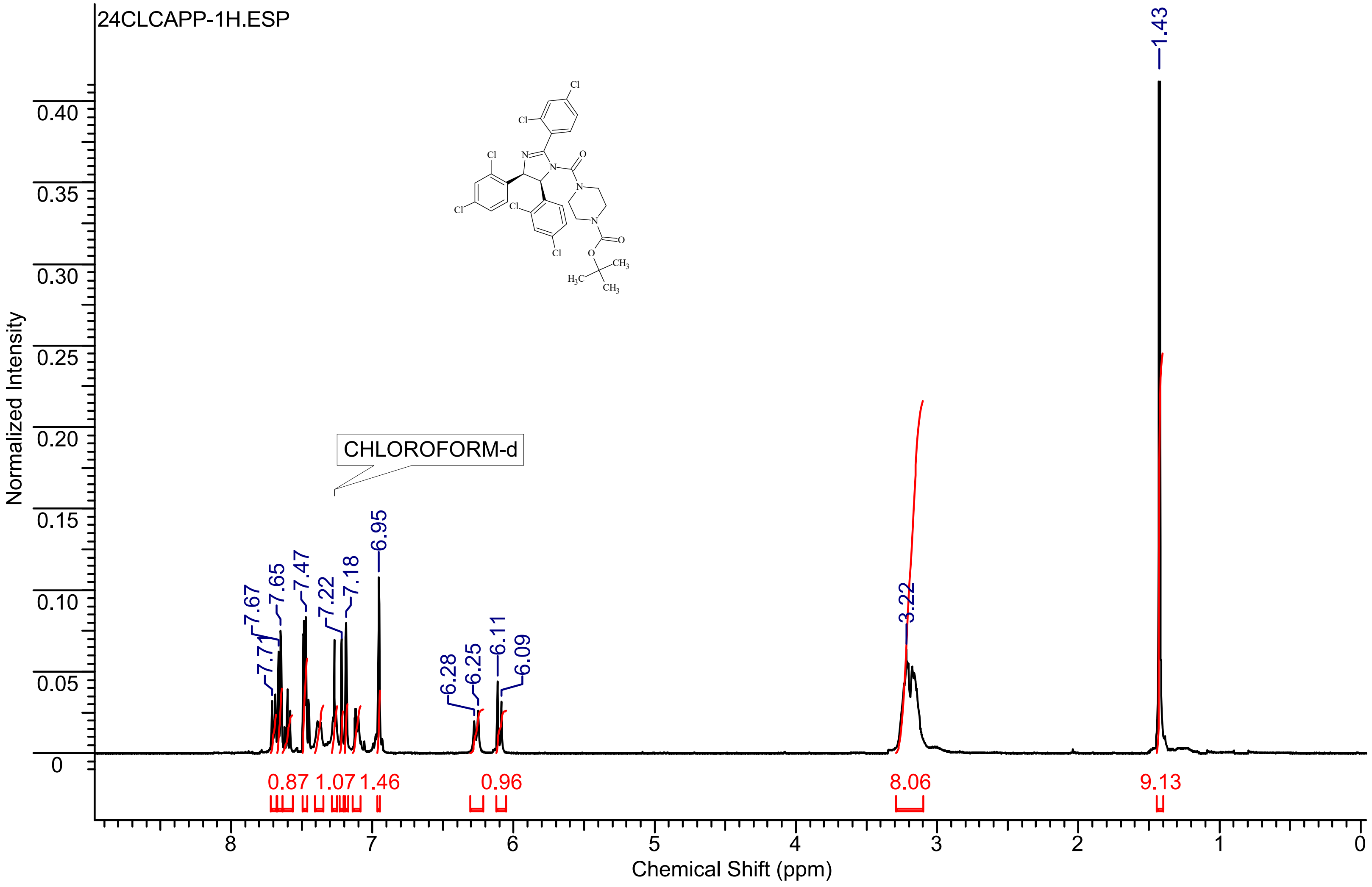

13C NMR *tert*-Butyl 4-(*cis*-2,4,5-*tris*(2,4-dichlorophenyl)-4,5-dihydro-*1H*-imidazole-1-carbonyl)piperazine-1-carboxylate (2aa).

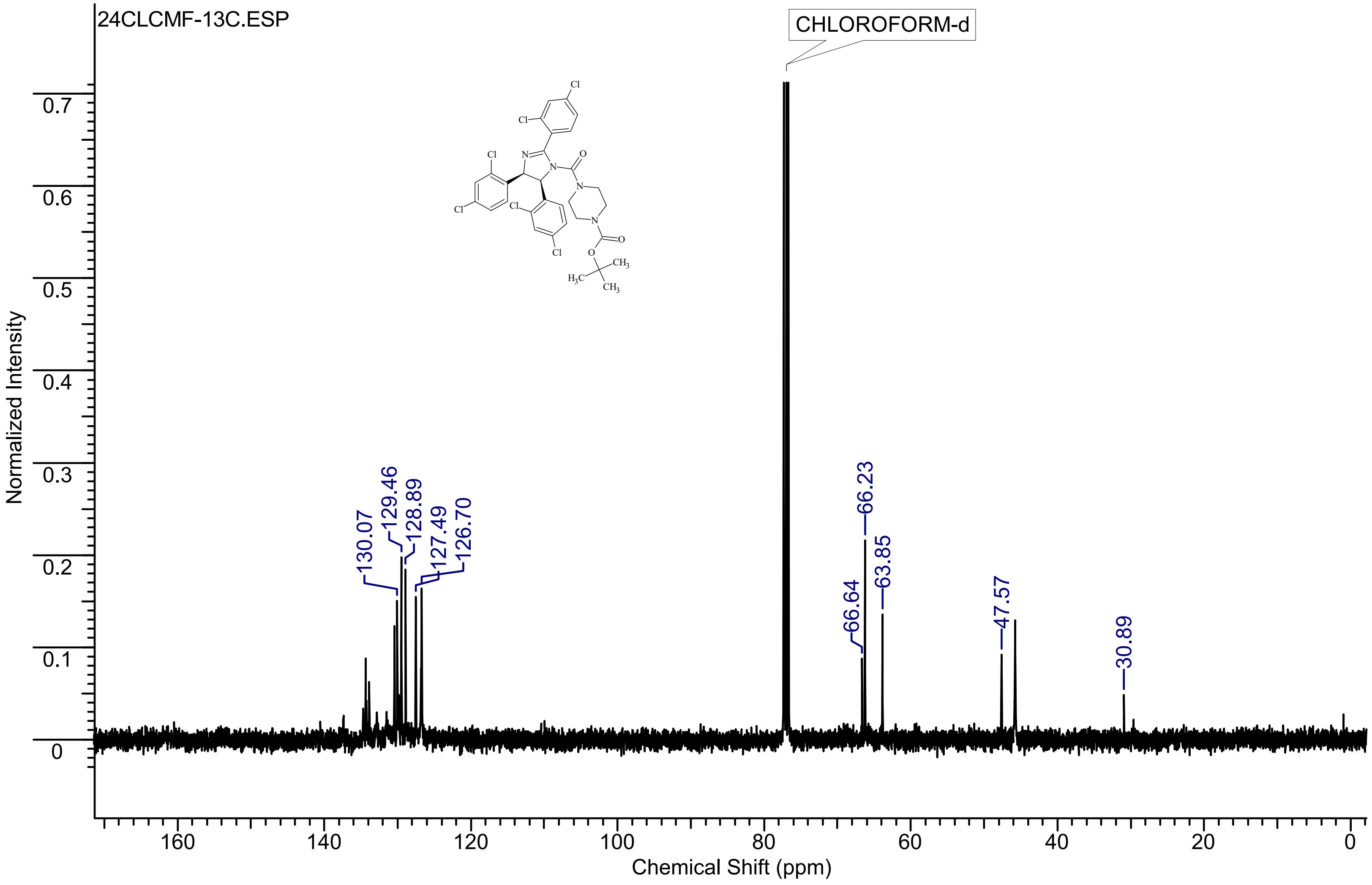

Supplement: Supplementary file 1 [file pharmaceuticals-15-00444-s001.zip › pharmaceuticals-1635037-supplementary.pdf]
